# Overcoming Automatic Behavioral Tendencies in Approach‐Avoidance Conflict Decisions

**DOI:** 10.1111/psyp.70101

**Published:** 2025-07-04

**Authors:** Menghuan Chen, Mario Reutter, Paul Pauli, Matthias Gamer, Andre Pittig

**Affiliations:** ^1^ Department of Psychology University of Würzburg Würzburg Germany; ^2^ Translational Psychotherapy Institute of Psychology, University of Göttingen Göttingen Germany

**Keywords:** approach‐avoidance conflict, gaze dispersion, inhibitory control, motivated behavior, response time

## Abstract

Adequate control over automatic responses to affective stimuli is crucial for adaptive goal‐oriented behavior. However, it remains unclear how individuals overcome automatic approach‐avoidance tendencies to appetitive and aversive stimuli. Here we examined free versus forced approach‐avoidance decisions to four conditioned stimuli (CSs), which were previously paired with either a single aversive (avCS+) or appetitive outcome (appCS+), both (i.e., conflicting) outcomes (confCS+), or no outcome (neuCS−). These CSs were presented in an anticipation phase before participants could use a joystick to either approach and obtain CS‐specific outcomes or avoid without getting anything. Response times, subjective ratings, heart rate, and eye‐tracking data were recorded in *N* = 75 participants. Results revealed that for single outcomes, concordant responses (e.g., avoidance to the avCS+) were faster than forced discordant responses (e.g., approach to the avCS+). During anticipation, gaze fixations shifted towards the spatial location associated with the concordant response for single‐outcome stimuli (e.g., upward for avoidance of avCS+). Conflicting stimuli elicited intermediate behavioral and gaze patterns at the group level, while exploratory analyses revealed substantial individual differences: High avoiders (i.e., participants showing an overall high proportion of avoidance) exhibited slower approach responses and greater threat‐focused visual attention compared to low avoiders. Decreased heart rate in response to all CSs suggests a general preparation of behavioral responses, while increased pupil dilation during the anticipation of aversive stimuli indicates threat‐related processing. These findings suggest that competing outcomes can amplify individual differences in motivational salience and therefore might inspire clinical interventions focused on inhibiting disorder‐specific behavioral tendencies.

## Impact Statement

This study advances our understanding of how individuals control automatic approach and avoidance tendencies to appetitive and aversive stimuli. Merging behavioral, physiological, and oculomotor responses, it reveals that competing outcomes facilitate behavioral inhibition, which may inspire the development of clinical interventions targeting at enhancing inhibitory control and promoting adaptive behaviors.

## 1 Introduction

Automatic behavioral tendencies for approaching appetitive stimuli (e.g., rewards) and avoiding aversive stimuli (e.g., threats) enable individuals to respond quickly and efficiently in dynamic environments, which promotes survival. In real‐life scenarios, however, people oftentimes need to adequately control and overcome these automatic responses to affective stimuli. Sometimes individuals need to avoid what they like or approach what they fear. For example, individuals might need to get an injection to stay healthy despite their own anxiety. Importantly, excessive automatic avoidance or approach responses may become impulsive and maladaptive, as for example observed in anxiety or addiction disorders (Loijen et al. 2020). A major component of behavioral treatments for these disorders is to overcome such impulsive responses, for example, in fear and anxiety disorders to approach an aversive stimulus (Hedman‐Lagerlöf et al. 2019; Pittig, Heinig, et al. 2021; Pittig et al. 2020) and in substance use disorder to avoid highly rewarding drugs (Daigre et al. 2021; Volkow 2020). Thus, it is crucial to investigate how individuals manage to avoid rewarding stimuli and approach threatening stimuli, as results may provide valuable insights for improving the treatment of psychopathologies characterized by automatic approach or avoidance behaviors.

Overcoming automatic behavioral tendencies requires inhibitory control (Bari and Robbins 2013), a core executive function that involves physiological, attentional, cognitive, emotional, and behavioral regulatory processes to implement adaptive goal‐oriented behaviors (Diamond 2013). Several well‐known tasks have been used to measure inhibitory control, including Go/No‐Go (Simmonds et al. 2008), stop‐signal (Congdon et al. 2012), emotional Stroop (Williams et al. 1996), and Simon tasks (Simon and Rudell 1967; also see Kang et al. 2022 for a review). In these tasks, participants are required to respond to target (“go”) stimuli using a motor response (e.g., button press) while inhibiting responses to relevant (“no‐go”) stimuli or to overcome reaction conflicts caused by relevant but incompatible stimulus attributes that need to be inhibited to prevent incorrect responses. Error rate is a commonly used dependent variable in such tasks to evaluate the efficiency of inhibitory control. Some studies have employed a more intuitive approach‐avoidance task to examine inhibitory control. For instance, Volman et al. used an approach‐avoidance task (Volman et al. 2011), in which participants were instructed to rapidly approach or avoid social emotional stimuli (i.e., happy and angry faces) by pulling or pushing a joystick towards or away from themselves, to assess the ability to overcome automatic emotional responses. The findings indicated that participants exhibited increased response times and a higher error rate when the required response was incongruent with the emotional stimulus (i.e., approach angry faces and avoid happy faces).

Although such paradigms have shed light on the inhibition of automatic behavioral tendencies at the end of decision processes, they only provide limited insights into the inhibitory control mechanisms operating during the anticipation and thus the selection of behavioral tendencies. This gap in understanding becomes more pronounced when considering the nature of real‐world contexts, where behaviors and their outcomes are usually interconnected in a complex manner, and behavioral tendencies typically emerge based on anticipated outcomes (Knutson and Greer 2008). Once an individual learns the association between a stimulus and a particular outcome, the presence of the stimulus can trigger similar response tendencies, as the learned association activates cognitive and physiological processes that guide behavior in anticipation of the expected outcome (Baumeister et al. 2007; Inzlicht et al. 2015). Furthermore, there is another critical clinical aspect that suggests a strong relevance of the anticipatory phase. The aberrant control of approach and avoidance and the excessive occurrence of specific behaviors in different psychopathologies (e.g., addiction and anxiety disorders; Loijen et al. 2020) extend beyond responses to the outcome (i.e., unconditioned stimulus, US), but also involve responses to substance‐ and threat‐related cues (i.e., conditioned stimuli, CSs; Boschet et al. 2022; Perry et al. 2014; Pittig and Scherbaum 2020). Anticipation is thus crucial in determining which behavioral tendencies are activated and how they can be controlled.

Moreover, naturalistic situations often include stimuli signaling mixed outcomes; thus, a single behavior can lead to both positive and negative consequences simultaneously. This duality of outcomes constitutes approach–avoidance conflicts, which may be based on diverging decision‐making processes (Corr 2013; Zorowitz et al. 2019). On the one hand, the positive outcome of an action serves as a strong incentive, propelling the individual toward taking an approach action. On the other hand, the negative outcome acts as a deterrent, urging the individual to avoid the same action. Such conflict can lead to heightened stress and indecision, as the individual struggles to reconcile these competing motivations (Carver and White 1994; Gray 1990). The resolution of such conflicts requires sophisticated cognitive processing, in which inhibitory control plays a crucial role (Barker et al. 2019).

Anticipation of approach‐avoidance responses is associated with specific cognitive processes and characterized by specific physiological responses. Studies have illustrated that bradycardia (a decrease in heart rate), induced by imminent threat, characterizes a protective state known as the defensive freezing response (Roelofs 2017; Tovote et al. 2005). Importantly, recent studies have consistently demonstrated that the transient bradycardia is accompanied by decreased gaze dispersion in anticipation of avoidable threat but not achievable reward (Merscher et al. 2022; Rösler and Gamer 2019). This pattern of reduced visual exploration was shown to be an adaptive response to prepare for defensive actions. Additionally, changes in pupil diameter were observed, with pupil dilation occurring not only during anticipation of threats (Bradley et al. 2008; Merscher et al. 2022) but also during rewards (Merscher et al. 2022; Schneider et al. 2018).

**FIGURE 1:**
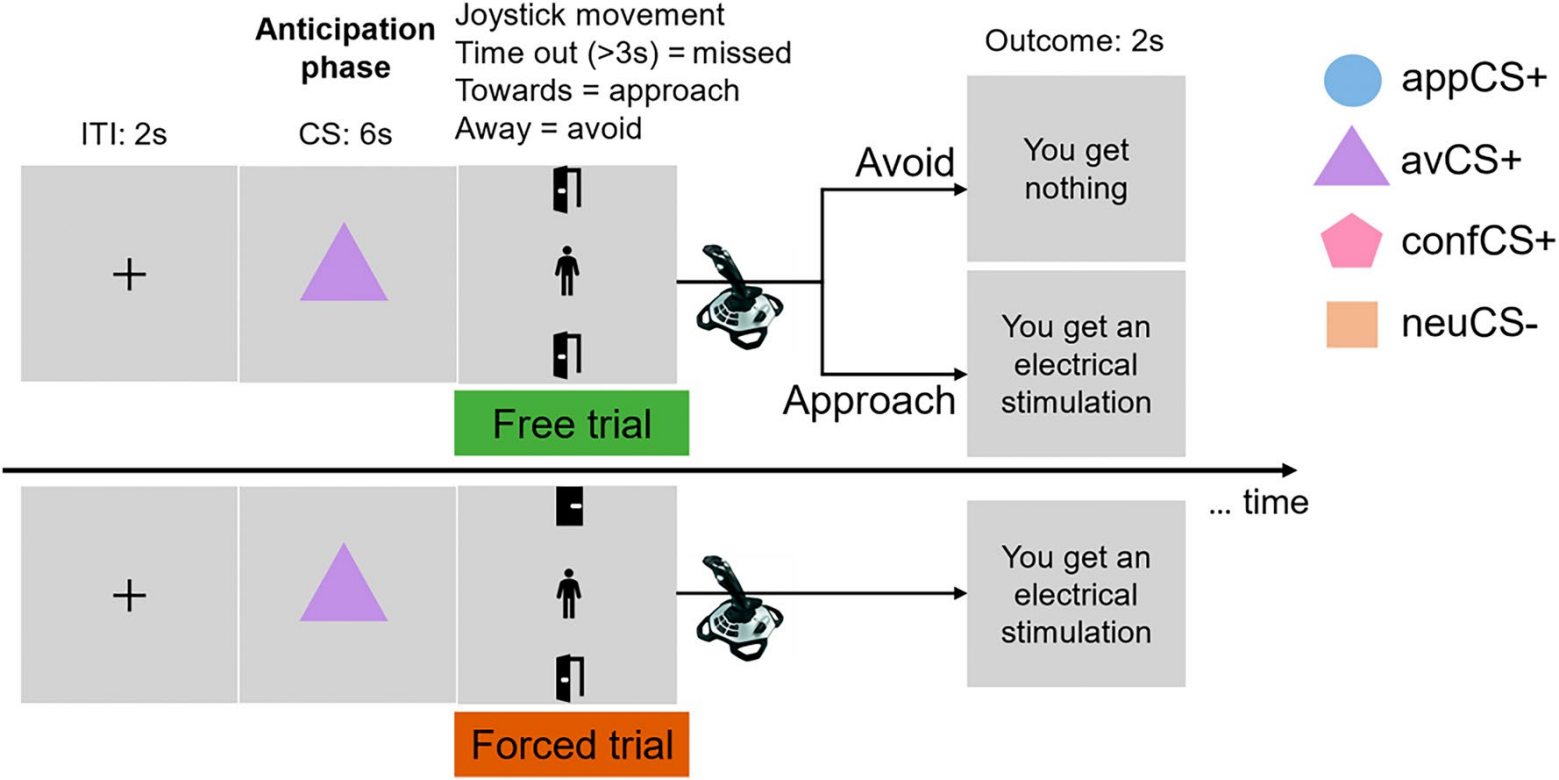
Timeline of an example trial of the free versus forced approach‐avoidance conflict task. In each trial, participants were presented with the black fixation cross for 2 s, followed by one of four possible CS for 6 s as anticipation phase. A manikin then appeared in the center of the screen, with an open and/or closed door at the top and bottom of the screen for a maximum of 3 s, where participants selected their choice by moving the manikin into an open door using a joystick. In free trials, both doors were open, and participants were free to decide whether to approach or avoid. In forced trials, however, only one door was open that participants were forced to approach (i.e., only the bottom door was open) or avoid (i.e., only the top door was open). During anticipation, participants had no information about whether the trial was free or forced until the response phase began.

Merging the different lines of evidence, the current study examined how individuals overcome automatic behavioral tendencies triggered by affective stimuli (i.e., approach threatening stimuli and avoid rewarding stimuli) and how they solve situations signaling competing outcomes (i.e., conflicting stimuli). To address these questions, we developed a free versus forced approach‐avoidance conflict (AAC) task, comprising three distinct phases: anticipation, response, and outcome delivery (Figure 1). Importantly, before implementing the AAC task and after a few habituation trials, participants completed a short acquisition training to acquire the specific contingencies between distinct geometric shapes (CSs) and outcomes (USs). Specifically, one CS (appCS+) was paired with an appetitive US (i.e., monetary reward, appUS), a second CS (avCS+) was associated with an aversive US (i.e., electrical stimulation, avUS), a third CS (confCS+) was associated with both the aversive and appetitive USs (conflicting US, confUS), and the fourth CS (neuCS−) served as a neutral CS and was never paired with any outcomes (no outcome, noUS). In the AAC task, participants selected a response by controlling the position of a manikin on the screen. Pulling the joystick towards themselves signaled approach, and pushing it away indicated avoidance. Free versus forced action trials were created by manipulating the availability of approach and avoidance options during the response phase. That is, both options were available in free trials and participants could freely decide whether to approach or avoid. In forced trials, only approach or avoidance was available, and participants were forced to perform the respective response. Depending on the CS type presented in the anticipation phase, approach behavior would lead to the corresponding outcome acquired in the previous acquisition training, and avoidance behavior would yield no outcome regardless of the CS type. We recorded physiological and oculomotor responses (i.e., heart rate, pupil diameter, and eye movements) throughout the experiment, as well as response times and continuous movement trajectories of the joystick in the AAC task. Valence and arousal ratings were reported after habituation, acquisition training, and the AAC task. We hypothesized that individuals would take longer to overcome previously established automatic behavioral tendencies, and we anticipated that competing outcomes would reduce this response time cost. We also explored gaze dynamics and physiological changes during the anticipation of the approach‐avoidance responses.

## 2 Methods and Materials

### 2.1 Participants

A total of 75 participants (aged between 18 to 40 years [*M* ± SD = 24.15 ± 4.45]; 42 females) were included in the study. An additional 17 participants were recruited but had to be discarded from all analyses due to technical issues (*n* = 12), insufficient quality of eye‐tracking data (*n* = 4), or withdrawal from the experiment (*n* = 1). For the main hypotheses, power analysis (power = 0.95, *α* = 0.05, *d* = 0.5, two‐sided) indicated a total of 54 participants for the most critical paired *t*‐test (forced approach vs. forced avoidance) (Faul et al. 2007). Given the large individual differences in physiological responses, we decided to acquire additional data from 21 participants to be sensitive to smaller effects and to obtain more robust results. Participants were recruited from the university and the local community. Each participant provided written informed consent prior to the experiment and received partial course credit or at least 13.75€ for participation. Additionally, participants were informed that they could earn a maximum bonus of 5€ based on their performance. This bonus was awarded after the experiment according to their choices in the experimental task. Inclusion criteria comprised an age between 18 and 40 years, right‐handedness, and no prescription glasses that could be problematic concerning eye‐tracking. Exclusion criteria were current or history of psychosis, bipolar disorder, or pain‐related disorders, traumatic brain injury, intellectual disability, substance dependence or abuse, current use of psychotropic medication, any serious medical conditions, or pregnancy. Participants were free to withdraw from the study at any time. The study was approved by the ethics committee of the Department of Psychology at the University of Würzburg (GZEK 2022‐77). Table 1 shows the socio‐demographic and questionnaire data.

**TABLE 1:** Demographic and psychometric data of participants.

Variables	*N* (%)
Education	
Technical college entrance qualification	2 (2.7%)
General university entrance qualification	46 (61.3%)
Completed vocational training	1 (1.3%)
Bachelor's degree	20 (26.7%)
Master's degree/diploma	5 (6.7%)
Doctorate	1 (1.3%)

Variables	*M* (SD)	*Range*
General habits		
Cups of coffee per day	1.25 (1.17)	0–5
Glasses of alcohol per week	2.32 (2.76)	0–12
Cigarettes per day	0.47 (1.38)	0–7
Hours of physical activity per week	4.67 (2.80)	0–15
Behavioral Inhibition (BIS)/Approach System Scale (BAS)		
BIS	19.83 (3.36)	10–26
BAS Drive	12.09 (2.12)	6–16
BAS Fun Seeking	12.24 (2.05)	7–16
BAS Reward Responsiveness	16.72 (2.17)	11–20
Depression, Anxiety, and Stress Scale (DASS‐21)		
Depression (DASS‐D)	4.32 (4.41)	0–22
Anxiety (DASS‐A)	3.12 (3.62)	0–16
Stress (DASS‐S)	8.24 (5.56)	0–23
Intolerance of uncertainty (IU‐18)	39.60 (11.47)	19–67

Abbreviations: BIS/BAS = Behavioral Inhibition/Approach System scale (Carver and White 1994). DASS‐21 = Depression Anxiety Stress Scale‐21 (Nilges and Essau 2015). IU‐18 = Intolerance of Uncertainty (Gerlach et al. 2008).

### 2.2 Stimuli and Calibration

In the experimental task, four neutral geometric shapes with equal size and brightness served as conditioned stimuli (CSs), including a blue circle (RGB: 142, 180, 227), a purple triangle (RGB: 180, 142, 227), a yellow square (RGB: 227, 180, 142), and a pink pentagon (RGB: 227, 142, 180). In addition, an individually calibrated aversive electrical stimulation served as the aversive unconditioned stimulus (avUS) and an individually calibrated small monetary reward as the appetitive US (appUS).

The avUS were applied to the medial side of the left forearm via a Wasp Electrode (Specialty Development; Bexley, UK). The electrical stimulation consisted of 125 pulses separated by 5 ms generated by a DS7A Digitimer stimulator. The intensity of the electrical stimulation was calibrated for each participant, starting at a low level of 0.2 mA with a gradual increase until the avUS was perceived twice as a 4 (definitely unpleasant but not painful) on a scale from 1 (not unpleasant) to 5 (too unpleasant) (Boschet et al. 2022). Calibration resulted in a mean final intensity of 0.8 mA (SD = 0.5 mA). For the appUS, a reward calibration procedure was conducted immediately after calibrating the intensity of the avUS for each participant (Wong and Pittig 2022). Specifically, participants were presented with a series of questions “Are you willing to pay__cents in order to avoid the electrical stimulation?” with the amount of reward ranging from 4 to 30 cents in even numbers (i.e., 4, 6, …, 28, 30 cents) in a randomized order. Participants were required to answer either “yes” or “no” to each question. The final amount of the competing reward was determined by taking the average of the lowest amount that received a “No” and the highest amount that received a “Yes”. This resulted in a mean competing reward of 14.21 cents (SD = 9.28 cents). The competing reward was meant to balance the incentive of reward‐seeking and threat‐avoidance in trials with conflicting reinforcement. Participants were instructed that the money gained in each trial was aggregated (up to a maximum of 5€) and paid at the end of the experiment.

### 2.3 Procedure and Paradigm

After providing written informed consent, participants first completed several questionnaires on individual differences which might influence task performance (see Table 1). Sociodemographic data included age, sex, education, and health‐related behaviors (e.g., smoking, physical activity, coffee, and alcohol consumption). Questionnaires assessed intolerance of uncertainty (IU‐18; Dugas et al. 2004; Gerlach et al. 2008), depression, anxiety and stress (German version of DASS‐21; Lovibond and Lovibond 1995; Nilges and Essau 2015), and behavioral inhibition and approach system (BIS/BAS; Carver and White 1994). Afterwards, participants were instructed to sit in a chair inside an acoustically shielded cabin, electrodes for ECG recording and aversive electrical stimulation delivery were attached, and the calibration procedures for the avUS and appUS were accomplished. Next, the experimental task was completed, which consisted of three stages: habituation, acquisition training, and the approach‐avoidance conflict (AAC) task. The eye‐tracker was calibrated prior to each stage. Participants were asked to rate the stimuli in terms of valence and arousal after each stage and contingency between CSs and USs after acquisition training and AAC task (see details below). Information about the contingency between CSs and USs was not disclosed to participants before the acquisition training. Eye‐tracking and ECG data were recorded throughout the whole experiment (see details in “data recording and processing”).

#### 2.3.1 Habituation

The purpose of the habituation was to familiarize participants with the four geometric shapes, which were later used as CSs to minimize any potential novelty or orientation effects. To this end, each stimulus was presented twice in a randomized order for 6 s, followed by an intertrial interval (ITI) with a black fixation cross presented in the center of the screen for 2 s.

#### 2.3.2 Acquisition Training

Acquisition training consisted of 6 trials per CS and aimed to enable participants to learn four specific CS‐US contingencies. For aversive CS+ trials (avCS+), one CS was followed by the aversive US in all trials. For appetitive CS+ trials (appCS+), a second CS was followed by the appetitive US in all trials. For conflicting CS+ trials (confCS+), a third CS was concurrently followed by both the aversive and appetitive US. Finally, for neutral CS‐ trials (neuCS−), the fourth CS was never followed by any US. The association between a specific CS (blue circle, purple triangle, yellow square, pink pentagon; see Figure 1) and the different outcomes was counterbalanced across participants. CSs were presented for 6 s in a randomized order, and the corresponding US was delivered 5 s after CS onset, with an ITI varying randomly between 5, 6, and 7 s.

#### 2.3.3 Approach‐Avoidance Conflict Task (AAC Task)

Following acquisition training, participants completed the free versus forced approach‐avoidance conflict task (see Figure 1). During the task, a CS was presented in the center of the screen for 6 s in the anticipation phase. This was followed by the response phase, where a manikin appeared in the center of the screen with an open and/or closed door at the top and the bottom of the screen, respectively. Participants were instructed to use a joystick to move the manikin into either the bottom‐open or top‐open door. Approach responses were instructed as pulling the joystick towards themselves to move the manikin into the bottom‐open door. Successful approach responses resulted in the outcome associated with the specific CS (avUS, appUS, confUS, or noUS). Avoidance responses were instructed as pushing the joystick away from themselves to move the manikin into the top‐open door. Successful avoidance responses yielded no outcome regardless of the CS type. Participants had a maximum of 3 s to complete the movement. Once the manikin reached the available door area, the outcome was presented for 2 s, followed by an ITI of 2 s. Trials in which no response was made within 3 s, were followed by the feedback “You missed the trial.” These trials were considered as omitted trials and excluded from all further analyses. Prior to administrating the AAC task, participants were familiarized with the joystick movement with four practice trials without USs. Participants were instructed to respond as fast as possible and that missing trials would result in a reduction of the total bonus earned.

Importantly, the doors were either displayed as “open” or “closed,” which was used to establish free versus forced trials. In free trials, two open doors were displayed, indicating that both approach and avoidance responses were available. Participants could freely choose whether to approach or to avoid. In forced trials, only one option was available (i.e., only one door was open), thus participants were forced to perform the only available response. If they tried to perform the unavailable response, the manikin would not move. During the anticipation phase, participants had no information about whether the trial was free or forced. This was only apparent when the response phase started.

The task included 80 trials. Each CS was presented in 20 trials, and half of them were free trials. Among the 10 forced trials, 5 trials were forced approach, while the other 5 trials were forced avoidance. For each trial, the position of the joystick was initialized to its neutral position of (0, 0). Trial conditions were presented in a randomized order for each participant. The task was divided into two blocks, with at least one minute of rest between them.

#### 2.3.4 Ratings

Participants were asked to rate the valence and arousal of each CS and US on a visual analogue scale from 1 (very unpleasant/calm) to 10 (very pleasant/exciting) after each stage. The corresponding CS‐US contingencies were evaluated after completing the acquisition training and the AAC task, respectively. Participants were presented with the CS and a question concerning the US expectancy (i.e., “How likely do you think this shape is followed by the electrical stimulation,” “the money,” “both the electrical stimulation and the money,” or “nothing”? The questions were presented in a pseudo‐randomized order for all CSs.) Participants gave their ratings by clicking on a visual analogue scale ranging from 0% (Certainly not) to 100% (Definitely) with a mouse.

### 2.4 Apparatus

Stimuli were displayed on a 24‐in Asus VG248QE monitor with 1920 × 1080‐pixel resolution and a refresh rate of 144 Hz, with a gray background (RGB: 210, 210, 210). A chin rest of the eye‐tracking device was used to maintain the head position and viewing distance between the eyes and the monitor at 60 cm. A joystick (Extreme 3D Pro Precision, Logitech International S.A., Switzerland) was used to allow for continuous movement of the manikin, and the trajectories were sampled at 60 Hz during the approach‐avoidance conflict task. We only allowed for vertical movements (resulting values ranged between −0.5 and 0.5 for the lower and upper border of the screen, respectively) by setting the *x*‐position to 0. The experiment was programmed using the PsychoPy 2022.2.3 library (Peirce 2008).

### 2.5 Data Recording and Processing

#### 2.5.1 Behavioral Responses

During the approach‐avoidance conflict task, the proportion of avoidance responses and response times were calculated as dependent variables. Response times were measured as the duration between the onset of the manikin and its arrival at the available door area. The continuous movement trajectories of the joystick were also recorded. Omitted trials, in which participants failed to respond within 3 s, were excluded from all further analyses (1.25%). Trials in which the *y*‐coordinate of the joystick was not at 0 at manikin onset were also excluded from all further analyses (4.75%) as well as trials with implausible response times (defined as < 200 ms, 0.03% of trials).

We also inspected whether the continuous joystick movement measures (including response latency, execution time, peak velocity, acceleration time, average movement velocity, and trajectory length, see Table S4) differed as a function of the experimental manipulations and the participants' behaviors (i.e., forced approach and avoidance, see Figure S2 for results).

#### 2.5.2 Eye‐Tracking

Eye movements and pupil diameter of the right eye were measured using an EyeLink 1000 Plus system (SR Research Ltd., Ottawa, Canada) in the desktop mount configuration with a sampling rate of 1000 Hz. The eye tracker was calibrated and validated using a 9‐point grid before starting every stage of the experiment. The recorded eye‐tracking data were converted into MAT files using Edf2Mat Matlab Toolbox (Etter and Biedermann 2016). Gaze position data were then parsed into saccades and fixations. Saccades were defined as fast eye movements with a velocity exceeding 30° s^−1^ or an acceleration exceeding 8000° s^−2^. Time periods between saccades were defined as fixations.

##### 2.5.2.1 Pupil Diameter

We extracted the pupil diameter from the eye‐tracking data. First, we checked the number of missing data points of raw pupil data in each trial, which could be caused by saccades, blinking, or the eye‐tracker accidentally failing to track the eye. Trials with more than 30% of missing data were excluded from all further analyses (habituation: 0.83%; acquisition training: 1.00%; AAC task: 1.13%). Furthermore, if more than half of the trials of a participant's pupil data were invalid, the participant would be excluded from all analyses (which, however, did not occur). Subsequently, we converted the pupil diameter from arbitrary units to millimeter (mm) according to Hayes and Petrov (2016). Missing segments of the resulting pupil signal were linearly interpolated, and the whole tracing was smoothed using a zero‐phase low‐pass filter with a cutoff frequency of 4 Hz (Kret and Sjak‐Shie 2019). We then calculated changes in pupil diameter relative to a baseline period of 1 s preceding CS onset. The resulting values were then averaged into 12 bins of 0.5 s each, spanning the CS duration. We used a smaller bin duration for pupil size than for the other oculomotor and physiological measures as the pupil responds more rapidly to both internal and external events (Merscher et al. 2022).

##### 2.5.2.2 Center Bias and Gaze Position

From the fixation data, we calculated the global center bias, which was defined as the average absolute distance of fixations from the center of the screen in pixels. Moreover, we computed the average horizontal and vertical gaze position to analyze systematic biases in visual exploration. The last 300 ms prior to CS onset were considered as baseline and used for drift correction. To ensure that participants fixated on the center of the screen at trial onset, an iterative drift correction algorithm was applied to the fixation data separately for *x*‐ and *y*‐coordinates. Specifically, the highest and lowest values of baseline coordinates were temporarily removed from the distribution of the baseline data and were marked as invalid when they deviated more than three standard deviations from the mean of the remaining baseline data. This procedure was repeated until no further x‐ and y‐values were marked as invalid. For trials with invalid baseline fixation data, the baseline was interpolated using the mean *x*‐ and *y*‐coordinates of the remaining trials with valid baseline fixation data (habituation: 24.50%; acquisition training: 10.61%; AAC task: 10.57%). Individual gaze drift was finally corrected by subtracting the *x*‐ and *y*‐coordinates during the baseline, respectively, from the gaze coordinates during CS presentation for each trial. A similar procedure was established in previous studies of our group (Merscher et al. 2022; Rösler and Gamer 2019). Subsequently, we calculated the center bias as well as the relative horizontal and vertical gaze position on a second‐by‐second basis.

#### 2.5.3 Electrocardiogram

An electrocardiogram (ECG) was recorded continuously using a BIOPAC MP160 device (BIOPAC Systems Inc.) at a sampling rate of 2000 Hz with disposable Ag/AgCl electrodes placed on the right clavicle and the lower left ribcage. The reference electrode was placed on the right lower ribcage. Data preprocessing was performed in R 4.1.2 (R team 2022). A 2 Hz high‐pass filter was first applied to remove slow signal drifts. Afterwards, R‐peaks were detected semi‐automatically according to their amplitude with an option for manual editing in case of detection errors (Reutter and Gamer 2023). The resulting R‐R intervals were then processed in MATLAB R2022b (Mathworks, Natick, MA, USA). Heart rate in beats per minute (BPM) was calculated based on the adjacent R–R intervals, and a real‐time scaling procedure was implemented to calculate mean heart rate for six one‐second time bins during CS presentation in each trial and each stage of the experiment (i.e., during habituation, acquisition training, and the anticipation phase of the AAC task). To account for individual differences in heart rate, we used the last second prior to CS onset as a baseline and subtracted it from following time bins.

### 2.6 Statistical Analyses

Statistical analyses were carried out using JASP (version 0.18.1), IBM SPSS Statistics for Windows version 26.0 (IBM Corp., Armonk, NY, USA), and Matlab R2022b, with an a priori significance level of *α* = 0.05. We conducted repeated measures analysis of variance (rmANOVA) with Greenhouse–Geisser correction of degrees of freedom for all dependent variables. Significant effects and interactions were followed up with simple main effects and Bonferroni‐corrected pairwise *t*‐tests. Effect size is reported as partial *η* ^2^ (*η*_p_^2^) for rmANOVAs and Cohen's *d* for *t*‐tests.

Concerning ratings, valence and arousal ratings were analyzed with 4 × 3 rmANOVA containing the within‐subject factors CS type (appCS+, avCS+, confCS+, and neuCS−) and stage (habituation, acquisition training, and the AAC task). Contingency ratings after acquisition training and the AAC task were analyzed with comparable 4 × 2 rmANOVA excluding the habituation stage.

In terms of behavioral data, we first compared the proportion of avoidance responses during free trials using a one‐way rmANOVA with CS type as a within‐subject factor. During free trials, we expected very few avoidance responses to the appCS+ and very scarce approach responses to the avCS+. Therefore, free trials would not provide sufficient variance to compare approach versus avoidance response times (RTs) across all different CSs. Therefore, RTs were only compared during forced trials using a 4 × 2 rmANOVA with CS type and response type (forced approach and forced avoidance) as within‐subject factors.

For the physiological and oculomotor responses during the habituation, acquisition training, and anticipation phase in the AAC task, 4 × 6 or 4 × 12 rmANOVAs, respectively, were conducted with CS type and time (6 s divided into 6 or 12 bins) as within‐subject factors. To specifically compare each emotional CS (i.e., appCS+, avCS+, confCS+) to the neutral CS, post hoc *t*‐tests were performed using false discovery rate correction (Benjamini and Hochberg 1995) to adjust for alpha‐error accumulation.

This study was preregistered at the Open Science Framework (https://osf.io/wgvk8), and the primary analyses follow the preregistration. We originally planned to also conduct analyses of the response time data using a drift diffusion model, but we refrained from this approach due to insufficient trial numbers in the AAC task. Furthermore, based on the results of the main analyses, we chose to deviate from the preregistered plan for the originally intended exploratory analyses and instead conducted an alternative set of exploratory analyses. These deviations are clearly indicated below.

## 3 Results

### 3.1 Ratings

**FIGURE 2:**
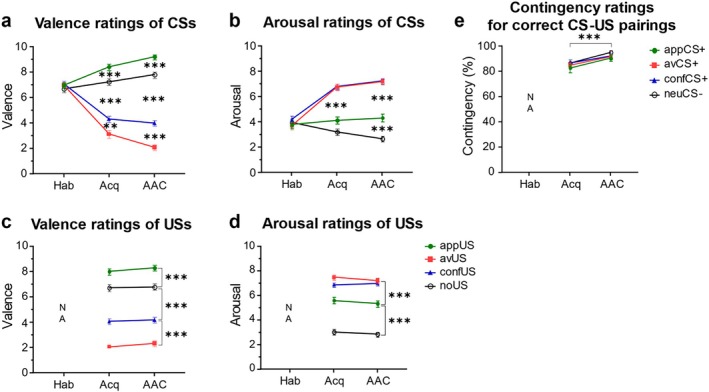
Ratings of valence (a and c), arousal (b and d), and contingency (e) of stimuli after the habituation stage (Hab), the acquisition training (Acq), and the AAC task. Error bars indicate standard errors of the mean. NA indicates that ratings were not acquired after a specific stage; please note that in (e) only the contingency ratings for the actual outcome paired with each CS are shown, that is, the displayed contingencies are as follows: AvCS+ with avUS, appCS+ with appUS, confCS+ with confUS, and neuCS− with noUS. **p < 0.01, ***p < 0.001.

The rating results indicate that participants differentially evaluated the CSs (see Figure 2a,b) and acquired the correct CS‐US contingencies after the acquisition training (Figure 2e). The 4 × 3 rmANOVA with CS type and stage revealed significant interaction effects on valence, *F*(6, 444) = 85.91, GG‐ε = 0.748, *p* < 0.001, *η*_p_^2^ = 0.537, and arousal ratings, *F*(6, 444) = 38.89, GG‐ε = 0.616, *p* < 0.001, *η*_p_^2^ = 0.345 (further results are reported in Table S1). After the acquisition training, the avCS+ was rated significantly more negative, and arousing compared to the appCS+ and neuCS−. The appCS+ also yielded significantly higher valence ratings compared to the other CSs. These differences slightly increased after the AAC task. While the appCS+ only elicited descriptively higher arousal than the neuCS− after the acquisition training, this difference was significant after the AAC task. As expected, the confCS+ was rated significantly more positive than the avCS+ and significantly more negative than the appCS+ and neuCS−. Interestingly, arousal ratings were comparable between the confCS+ and the avCS+. Descriptive statistics of ratings are provided in Table S2, and post hoc comparisons are reported in Table S3.

Regarding 4 × 2 rmANOVA with US type and stage on US ratings, significant main effects of US type were observed for valence, *F*(3, 222) = 171.03, GG‐ε = 0.790, *p* < 0.001, *η*_p_^2^ = 0.698, and arousal ratings, *F*(3, 222) = 133.80, GG‐ε = 0.842, *p* < 0.001, *η*_p_^2^ = 0.644, indicating stable differences between the USs after acquisition training and AAC task (see Figure 2c,d, and Table S1). The appUS was rated significantly more positive and the avUS more negative than the other USs. The confUS yielded slightly but significantly higher valence ratings than the avUS. Arousal ratings were highest for the avUS and confUS, but higher ratings were also observed for the appUS as compared to the condition without any outcomes (noUS).

A comparable 4 × 2 rmANOVA with CS type and stage on contingency ratings only revealed a significant effect of stage, *F*(1, 74) = 11.45, *p* = 0.001, *η*_p_^2^ = 0.134 (for further results see Table S1 and Figure S1). In general, participants were aware of which CS predicted which outcome with no differences between CSs, and contingency ratings even increased after the AAC task (Figure 2e).

Overall, the results suggest that participants successfully learned the relationships between CSs and USs and evaluated the CSs in line with the learned outcomes after the acquisition training. Thus, the acquisition training successfully established the desired differentiation between the specific CSs, which further increased during the AAC task.

### 3.2 Behavioral Responses

**FIGURE 3:**
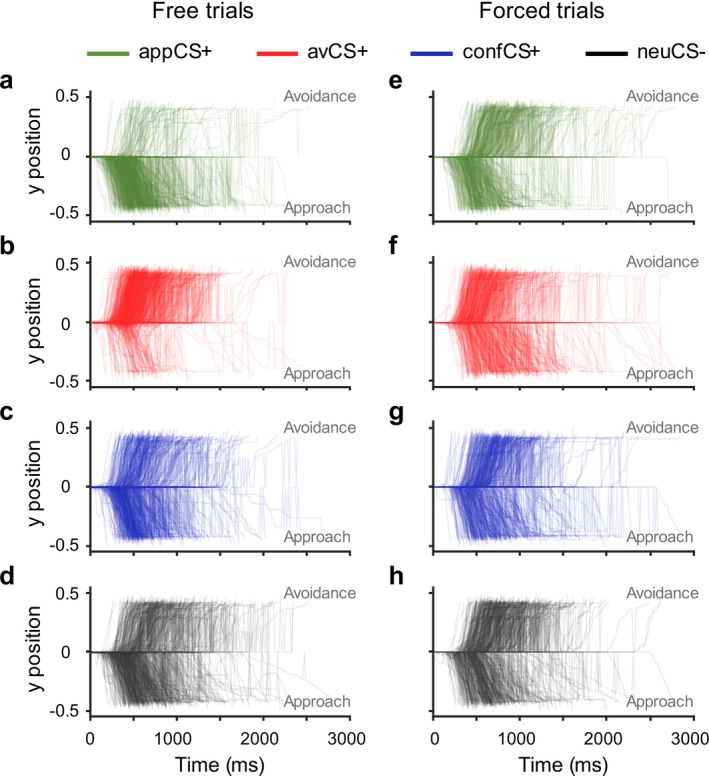
Individual movement trajectories in the free versus forced trials during the AAC task. In free trials, participants displayed the most frequent approach responses to appCS+ (a) and the most frequent avoidance responses to avCS+ (b). At the group level, the requencies of approach and avoidance responses for confCS+ (c) and neuCS‐ (d) were comparable. In forced trials, participants demonstrated evident delays in initiating joystick movement when forced to avoid the appCS+ (e) and approach the avCS+ (f). Delays in initiating movement were also observed in forced avoidance for confCS+ (g) and neuCS‐ (h).

The individual movement trajectories towards the approach or avoidance options in the free versus forced trials for all CSs in the AAC task are depicted in Figure 3. In the forced trials, participants demonstrated evident delays in initiating joystick movement (Figure 3e,f) and reduced velocity throughout its execution when required to perform discordant responses (i.e., avoidance to the appCS+, and approach to the avCS+, see also Figure S2a,e). Further details on movement characteristics (e.g., response latency, movement velocity, and acceleration) are reported in the Supporting Information (see Tables S4 and S5, and Figure S2).

#### 3.2.1 Proportion of Avoidance in Free Trials

**FIGURE 4:**
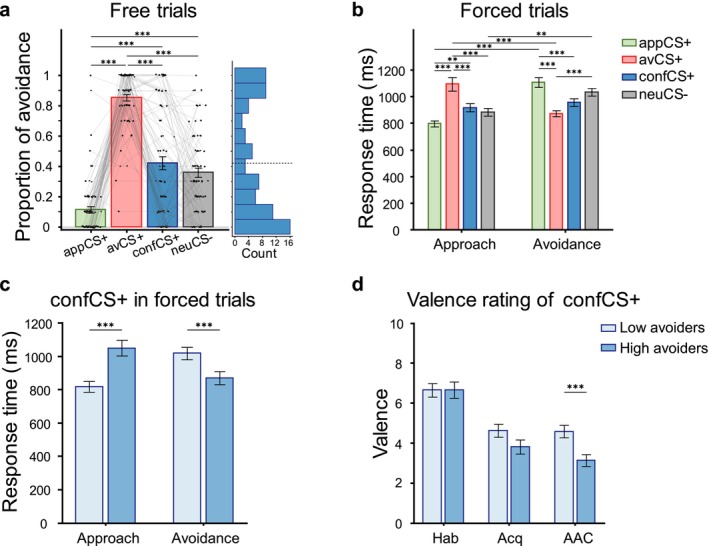
Behavioral responses in the free versus forced AAC task. (a) Proportion of avoidance in free trials. The dashed line in the histogram indicates the cutoff of high versus low avoiders. (b) Response times in forced approach and avoidance. (c) Response times of confCS+ (high versus low avoiders) in forced approach and avoidance. (d) Valence rating of confCS+ at the three stages of habituation (Hab), acquisition training (Acq) and the AAC task, respectively. Error bars indicate the standard errors of the mean. **p < 0.01, ***p < 0.001.

The one‐way rmANOVA on proportion of avoidance revealed a significant effect of CS type, *F*(3, 222) = 122.91, GG‐ε = 0.794, *p* < 0.001, *η*_p_^2^ = 0.624. As shown in Figure 4a, participants, as expected, avoided the appCS+ least frequently (*M* = 0.12, SD = 0.19) and the avCS+ most frequently (*M* = 0.85, SD = 0.18). Importantly, avoidance to the confCS+ (*M* = 0.42, SD = 0.37) was significantly higher than the appCS+ (*t*(73) = −7.21, *p* < 0.001, *d* = −1.16, 95% CI [−1.63, −0.69]), significantly lower than the avCS+ (*t*(73) = 10.00, *p* < 0.001, *d* = 1.64, 95% CI [1.11, 2.17]), and there was no significant difference between confCS+ and neuCS− (*t*(73) = 1.30, *p* = 1.000, *d* = 0.24, 95% CI [−0.16, 0.64]). This result suggests that the confCS+ was more ambivalent in triggering approach or avoidance responses.

#### 3.2.2 Response Times in Forced Trials

Because of very few approach responses to the avCS+ and avoidance responses to the appCS+ in free trials (see Figure 4a), we restricted our analyses of response times (RTs) to forced trials. The 4 × 2 rmANOVA on RTs with CS type and response type as factors revealed a significant main effect of response type, *F*(1, 74) = 18.71, *p* < 0.001, *η*_p_^2^ = 0.202, and a significant interaction of CS type × response type, *F*(3, 222) = 33.86, GG‐ε = 0.680, *p* < 0.001, *η*_p_^2^ = 0.314, but no significant main effect of CS type, *F*(3, 222) = 2.32, GG‐ε = 0.902, *p* = 0.083, *η*_p_^2^ = 0.030 (see Figure 4b).

As expected, participants showed significantly shorter RTs for appCS+ in forced approach (*M* = 795.51 ms, SD = 191.32 ms) compared to forced avoidance (*M* = 1105.16 ms, SD = 324.41 ms; *t*(73) = −8.39, *p* < 0.001, *d* = −1.13, 95% CI [−1.65–0.61]) and significantly shorter RTs for avCS+ in forced avoidance (*M* = 871.67 ms, SD = 184.33 ms) compared to forced approach (*M* = 1092.18 ms, SD = 429.13 ms; *t*(73) = 5.98, *p* < 0.001, *d* = 0.81, 95% CI [0.33, 1.28]). These results indicate that the concordant behavioral responses were executed faster than the discordant responses.

Forced approach to the neuCS− (*M* = 882.22 ms, SD = 233.38 ms) was significantly faster compared to forced avoidance (*M* = 1032.51 ms, SD = 239.77 ms; *t*(73) = −4.07, *p* = 0.002, *d* = −0.55, 95% CI [−1.00–0.10]), which was solely attributed to shorter response latency in initiating joystick movement in forced approach than in forced avoidance (see also Figures 3h and S2a). Interestingly, for the confCS+, response times during forced approach (*M* = 915.93 ms, SD = 259.76 ms) compared to forced avoidance (*M* = 954.91 ms, SD = 242.51 ms) did not differ significantly (*t*(73) = −1.06, *p* = 1.000, *d* = −0.14, 95% CI [−0.57 0.29]). Importantly, comparing RTs to the four CSs separately within the two response types (i.e., forced approach and forced avoidance) showed that the confCS+ was linked to intermediate response times. In forced approach, responses to the confCS+ were significantly slower compared to the appCS+ (*t*(73) = −3.70, *p* = 0.007, *d* = −0.44, 95% CI [−0.83–0.05]) and significantly faster compared to the avCS+ (*t*(73) = 5.42, *p* < 0.001, *d* = 0.65, 95% CI [0.23 1.06]). In forced avoidance trials, the opposite pattern was found.

Taken together, the behavioral data suggest that participants quickly initiated forced concordant responses to stimuli linked to a single outcome (i.e., avoidance following a threat‐associated stimulus, approach following a reward‐associated stimulus). In contrast, forced discordant responses were characterized by prolonged response times. Importantly, competing outcomes seemed to facilitate the forced discordant responses.

Interestingly, individual data for the proportion of avoidance to the confCS+ in free trials revealed an approximately bimodal distribution (Figure 4a). Visual inspection indicates that some participants rarely avoided, while others persistently avoided in most free trials. As this pattern of a bimodal distribution aligns with a previous study in patients with anxiety disorders (Pittig, Boschet, et al. 2021), we aimed to explore potential factors contributing to frequent versus infrequent avoidance. To this end, we conducted additional exploratory analyses with independent samples *t*‐test, comparing participants categorized as high avoiders (with a proportion of avoidance ≥ mean proportion of 0.42, *n* = 32) with low avoiders (with a proportion of avoidance < 0.42, *n* = 43). Interestingly, high versus low avoiders did not differ in either the amplitude of the reward (*t*(73) = −1.77, *p* = 0.082, *d* = −0.41, 95% CI [−0.87 0.05]) or the intensity of the electrical stimulation (*t*(73) = −0.28, *p* = 0.777, *d* = −0.07, 95% CI [−0.524 0.392]). There were also no significant differences between high and low avoiders in other task‐related factors (valence of appCS+, avCS+, and neuCS−, arousal of all CSs, valence ratings of USs), heart rate, and pupil diameter changes during CS presentation, or sociodemographic data (see more details in Table S6).

However, significant differences were observed for response times and valence of the confCS+: high avoiders were significantly faster to avoid in forced‐avoidance trials than low avoiders (*t*(73) = 2.75, *p* = 0.007, *d* = 0.64, 95% CI [0.17, 1.11]), and significantly slower to approach in forced‐approach trials (*t*(73) = −4.26, *p* < 0.001, *d* = −1.00, 95% CI [−1.48, −0.51]; Figure 4c). Importantly, the faster forced avoidance response observed in high avoiders was specific to confCS+ but not to the other three CSs (see Table S7 and Figure S3). Furthermore, high avoiders compared to low avoiders rated the confCS+ more negatively after the AAC task (*t*(73) = 3.28, *p* = 0.002, *d* = 0.77, 95% CI [0.29, 1.24]; Figure 4d). These results indicate that high avoiders did not only show a higher proportion of avoidance to the confCS+, but also avoided it faster and evaluated it more negatively.

To further examine whether the group‐level effects of conflicting outcomes on response times were consistent within clusters of participants, we expanded the confirmatory analysis by conducting a 4 × 2 × 2 rmANOVA on RTs with CS type, response type, and the newly added factor subgroup (i.e., *n* = 32 High avoiders; *n* = 43 Low avoiders). This revealed a significant three‐way interaction (*F*(3, 219) = 6.068, *p* = 0.001, *η*_p_^2^ = 0.077), implying that the pattern of responses to different CS types depends on both the imposed response type by the paradigm but also by the individual response style reflected in the factor subgroup. To qualify this interaction, we conducted follow‐up one‐way ANOVAs to directly test how confCS+ responses (approach/avoidance) differ from avCS+/appCS+ within each subgroup (See Figure S3). The significant effects of CS type on RTs in all conditions (see Table S14) were followed by post hoc *t*‐tests (Table S15). Notably, distinct patterns emerged for the confCS+ between subgroups. For low avoiders, approach RTs to confCS+ were comparable to appCS+ (*t*(42) = −1.78, *p* = 0.499, *d* = −0.19, 95% CI [−0.50, 0.11]; Figure S3a), but significantly faster than avCS+ (*t*(42) = 4.68, *p* < 0.001, *d* = 0.99, 95% CI [0.33, 1.64]). Conversely, avoidance RTs to confCS+ were significantly slower than avCS+ (*t*(42) = −3.83, *p* = 0.003, *d* = −0.55, 95% CI [−0.99, −0.12]; Figure S3c) and marginally faster than appCS+ (*t*(42) = 2.52, *p* = 0.094, *d* = 0.37, 95% CI [−0.05, 0.79]). For high avoiders, however, approach RTs to confCS+ were significantly slower than appCS+ (*t*(31) = −5.19, *p* < 0.001, *d* = −0.76, 95% CI [−1.25, −0.27]; Figure S3b) and indistinguishable from avCS+ (*t*(31) = 0.42, *p* = 1.0, *d* = 0.06, 95% CI [−0.36, 0.48]). On the contrary, avoidance RTs to confCS+ matched avCS+ (*t*(31) = 0.10, *p* = 1.0, *d* = 0.01, 95% CI [−0.38, 0.40]; Figure S3d) and were significantly faster than appCS+ (*t*(31) = 4.80, *p* < 0.001, *d* = 0.96, 95% CI [0.30 1.63]). These results suggest that a competing threat moderately facilitated avoidance of reward in both subgroups, whereas a competing reward facilitated approach towards threat in low avoiders but not in high avoiders.

### 3.3 Oculomotor and Physiological Responses

**FIGURE 5:**
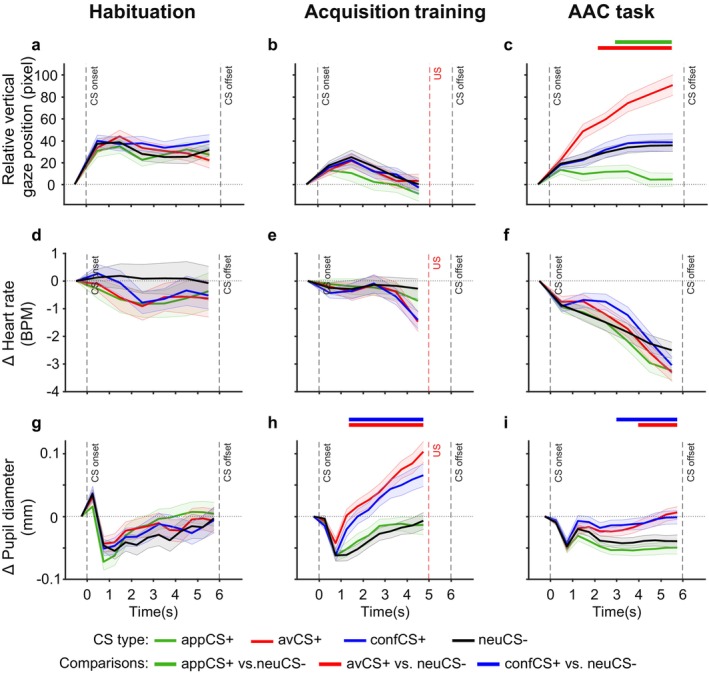
Oculomotor and physiological responses during CS presentation in the habituation stage, the acquisition training, and the anticipation phase in the AAC task. (a–c) Relative vertical gaze position. (d–f) Heart rate changes. (g–i) Pupil diameter changes. Shaded ribbons denote standard errors of the mean. Horizontal lines at the top of the plot indicate significant differences between the appetitive, aversive, and conflicting conditions with the neutral CS, respectively.

During the habituation stage, participants were familiarized with the different CSs but did not yet learn about their associations with the different outcomes. Consequently, we did not observe significant effects of the CS type on the oculomotor (Figure 5a, see also Table S8) and physiological responses (Figure 5d,g, see also Table S10). The following analyses therefore concentrate on the acquisition training and the AAC task.

#### 3.3.1 Center Bias and Gaze Position

From the analyses of the distance of fixations from the center of the screen (i.e., global center bias), we observed an increase of visual exploration after CS onset in the habituation stage, acquisition training, and the AAC task. However, significant differences involving CS type only emerged in the AAC task (see Figure S4 and Table S8). Consistent with the task that mainly required vertical scanning of the screen for the availability of response options, participants did not show substantial horizontal eye movements (Figure S4). Moreover, gaze positions remained relatively stable during the acquisition training (Figure 5b). Therefore, the following analyses focus on the relative vertical gaze positions in the AAC task.

During the anticipation phase in the AAC task (Figure 5c), the rmANOVA on relative vertical gaze position revealed significant main effects of CS type, *F*(3, 222) = 26.08, GG‐ε = 0.729, *p* < 0.001, *η*_p_^2^ = 0.261, and time, *F*(6, 444) = 37.53, GG‐ε = 0.378, *p* < 0.001, *η*_p_^2^ = 0.336, as well as a significant interaction of CS type × time, *F*(18, 1332) = 23.32, GG‐ε = 0.223, *p* < 0.001, *η*_p_^2^ = 0.220. Pairwise comparisons showed that participants shifted their gaze upwards for the avCS+ and downwards for the appCS+ relative to the neuCS− (see Table S9). Interestingly, gaze positions for the confCS+ were located between the avCS+ and the appCS+ with no difference to the neuCS−. This means that fixations during CS presentation were already directed towards the concordant response option (i.e., avoidance for the avCS+ and approach for the appCS+) even though participants did not yet know whether they would be permitted to execute this response or whether the current trial would require a forced discordant response (i.e., approach for the avCS+ or avoidance for the appCS+).

Interestingly, those participants that showed frequent avoidance to the confCS+ in free trials (see Figure 4a) also exhibited a greater vertical bias in their gaze positions for confCS+ compared to low avoiders (see Table S6). Across all participants, the average vertical gaze position during the anticipation phase for the confCS+ correlated with the proportion of avoidance to it in free trials (*r* = 0.49, *p* < 0.001).

#### 3.3.2 Heart Rate

During acquisition training (Figure 5e), the rmANOVA revealed a significant deceleration across time, *F*(5, 370) = 7.95, GG‐ε = 0.521, *p* < 0.001, *η*_p_^2^ = 0.097. However, there was no significant main effect of CS type, *F*(3, 222) = 0.50, *p* = 0.682, *η*_p_^2^ = 0.007, and interaction of CS type × time, *F*(15, 1110) = 1.68, GG‐ε = 0.525, *p* = 0.100, *η*_p_^2^ = 0.022.

During the anticipation phase in the AAC task, heart rate significantly decreased over time across all CS types (significant main effect of time, *F*(6, 444) = 53.61, GG‐ε = 0.429, *p* < 0.001, *η*_p_^2^ = 0.420). Moreover, we observed a significant interaction of CS type × time, *F*(18, 1332) = 5.56, GG‐ε = 0.417, *p* < 0.001, *η*_p_^2^ = 0.07. The main effect of CS type was not significant, *F*(3, 222) = 2.31, *p* < 0.077, *η*_p_^2^ = 0.03. Follow‐up pairwise comparisons revealed that heart rate mainly differed between CSs in the middle of the anticipation period with a less pronounced deceleration for the confCS+ (Figure 5f). More details on post hoc comparisons are provided in Table S11.

#### 3.3.3 Pupil Diameter

Overall, pupil diameter of all CS types transiently decreased within 1 s after CS onset. During acquisition training (Figure 5h), pupil diameter subsequently increased across time, which differed between the CSs (significant main effect of CS type, *F*(3, 222) = 13.51, *p* < 0.001, *η*_p_^2^ = 0.154, and time, *F*(10, 740) = 34.14, GG‐ε = 0.266, *p* < 0.001, *η*_p_^2^ = 0.316, and significant interaction of CS type × time, *F*(30, 2220) = 9.24, GG‐ε = 0.275, *p* < 0.001, *η*_p_^2^ = 0.111). Pupil diameters for the avCS+ and confCS+ increased more strongly, and they were significantly larger than responses to the neuCS− and appCS+ after 2 s of CS onset. Pupil diameters for the appCS+ and neuCS− did not differ significantly over time before the US presentation (see Table S12).

During the anticipation phase in the AAC task (Figure 5i), the pattern was roughly comparable to the acquisition stage, and the rmANOVA revealed significant main effects of CS type, *F*(3, 222) = 14.32, *p* < 0.001, *η*_p_^2^ = 0.162, and time, *F*(12, 888) = 8.79, GG‐ε = 0.184, *p* < 0.001, *η*_p_^2^ = 0.106, as well as a significant interaction of CS type × time, *F*(36, 2664) = 8.08, GG‐ε = 0.243, *p* < 0.001, *η*_p_^2^ = 0.098. Post hoc analyses (see Table S13) revealed that there was neither a significant difference in pupil diameter between appCS+ and neuCS− across the anticipation phase nor between avCS+ and confCS+. After the initial pupil constriction following CS onset, pupil diameter showed a stronger increase for the avCS+ and confCS+ compared to the neuCS− and appCS+. In sum, these results indicate that stimuli predicting potential aversive electrical stimulation induced higher physiological arousal indicated by pupil responses than stimuli predicting no aversive stimulation.

## 4 Discussion

The main goal of this study was to investigate how individuals overcome automatic behavioral tendencies to aversive and appetitive stimuli (i.e., approach threatening stimuli and avoid rewarding stimuli) and how they solve situations signaling competing outcomes. To this end, we developed an approach–avoidance conflict task that included free and forced behavioral choices. Thus, in some trials, participants could freely decide whether to approach and obtain a specific outcome or avoid and receive nothing instead. In forced trials, only one behavioral option was available, and participants had to either approach or avoid the outcome. The main findings demonstrate that, on a behavioral level, participants exhibited rapid concordant behavioral responses triggered by conditioned stimuli associated with a single outcome (reward or threat). Thus, we observed fast avoidance behavior in response to a threat‐associated stimulus and approach behavior in response to a reward‐associated stimulus. However, when forced to perform discordant responses, for example, approach behavior in response to a threat‐associated stimulus, response times increased significantly. This finding is consistent with previous studies where participants took longer to overcome automatic behavioral tendencies (Railo et al. 2023; Schützwohl 2018; Volman et al. 2011).

Importantly, a conflicting stimulus associated with competing outcomes, that is, both reward and threat, facilitated discordant responses. At the group level, we observed faster approach responses after a conflicting stimulus compared to approach responses after an aversive stimulus, and faster avoidance responses after a conflicting stimulus compared to avoidance responses after an appetitive stimulus. This response facilitation due to a stimulus signaling conflicting outcomes was also reflected in valence ratings and anticipatory eye movements. The valence of the conflicting stimulus was rated significantly more positive than that of the stimulus predicting an aversive outcome and significantly more negative than that of the stimulus signaling an appetitive outcome. Moreover, anticipatory eye movements triggered by the conflicting stimulus were more balanced and showed no difference from the neutral stimulus, which were located between those elicited by aversive and appetitive stimuli. Superficially, these results suggest that participants dynamically integrated the threat and reward information during approach–avoidance conflict, which facilitated the discordant behaviors.

Interestingly, however, we observed substantial individual differences in avoidance responses to conflicting stimuli in free trials. While some individuals showed persistent avoidance, others showed little to no avoidance. As intended by the individual calibration of the electrical stimulation and the reward amount, this result was not related to objective levels of the aversive or appetitive outcome. Moreover, no significant differences were observed between individuals with high versus low avoidance in other task‐related factors (valence ratings of stimuli), sociodemographic variables (age, biological sex), clinical characteristics (stress, anxiety, depression), and threat/reward sensitivity assessed by the Behavioral Inhibition/Approach System scale. However, high avoiders showed slower approach responses to conflicting stimuli compared to low avoiders and rated the conflicting stimuli more negatively after the approach‐avoidance conflict task. These results imply that high avoiders may have focused disproportionately on the adverse aspect of conflicting stimuli, and their impulsive avoidance behavior reinforced this perceptual bias (Pittig et al. 2020; Yamamori et al. 2023). Other aspects might have contributed as well, such as cognitive demand (Kool et al. 2010), decision‐making style (Fricke and Vogel 2020), and neural recruitment of the prefrontal cortex (Aupperle et al. 2015; Sánchez‐Bellot et al. 2022). As our findings were based on post hoc analyses, further research on larger samples is needed to pinpoint the origins of these individual differences.

Exploratory subgroup analyses on response times revealed a critical divergence in dealing with competing outcomes. Low avoiders exhibited similar approach responses to both the conflicting and appetitive stimuli, responding more quickly than to the aversive stimulus. Additionally, their avoidance responses to the conflicting stimulus were slower than those to the aversive stimulus but slightly faster than those to the appetitive stimulus. These results indicate that, in low avoiders, a competing reward facilitated approach towards threat and impaired avoidance of threat, while a competing threat only weakly facilitated avoidance of reward without disrupting approach towards reward. In contrast, high avoiders demonstrated slower approach responses to the conflicting stimulus compared to the appetitive stimulus, with response times similar to those for the aversive stimulus. Their avoidance responses to the conflicting stimulus were faster than those to the appetitive stimulus but comparable to those for the aversive stimulus. These results suggest that, in high avoiders, a competing reward neither enhanced approach towards threat nor impaired avoidance of threat, whereas a competing threat facilitated avoidance of reward and disrupted approach towards reward.

Taken together, these findings clarify the observed group‐level facilitation effect of conflicting outcomes on discordant responses. While a competing threat moderately facilitated avoidance of reward in both subgroups, a competing reward facilitated approach towards threat in low avoiders but not in high avoiders. This result aligns with clinical research underscoring that competing rewards have a weaker impact on approach‐avoidance behaviors in individuals with anxiety disorders compared to healthy controls (Boschet‐Lange et al. 2025). Importantly, the divergent effects observed in the current study—strong facilitation of a competing reward on approach towards threat in low avoiders but limited effect in high avoiders—may be due to preexisting biases. High avoiders rated the conflicting stimuli more aversive than low avoiders even before the approach‐avoidance conflict task (i.e., after the acquisition training). These results imply that individuals predisposed to threat vigilance disproportionately weight negative salience during conflict resolution. However, these subgroup findings remain exploratory, as this study was not designed to specifically address individual differences in reward‐threat attribution or avoidance propensity. Future studies are warranted to systematically investigate the mechanisms underlying these individual differences.

The current results have implications for behavior in real‐life situations, such as developing interventions or strategies to help individuals manage and modify their emotional responses in various contexts, from overcoming clinical anxiety to improving impulse control in addictive behaviors. Approach‐avoidance conflicts represent a highly shared characteristic of several mental disorders, and their disbalance in both directions can cause detrimental effects on emotional regulation and decision‐making (Letkiewicz et al. 2023; Smith et al. 2021). For example, this can result in compulsive approach to problematic substances along with irrational drug craving in addiction and elevated avoidance of social stimuli in social anxiety disorders even when patients explicitly express the desire to stop the respective maladaptive behaviors (Hogarth 2020; Loijen et al. 2020). Our experimental design allowed for a direct comparison of behavioral responses in scenarios of reflective approach but impulsive avoidance (i.e., when participants were forced to perform approach behavior after a stimulus signaling threat), and reflective avoidance but impulsive approach (i.e., when participants were forced to perform avoidance behavior after a stimulus signaling reward), as well as approach and avoidance responses following a conflicting stimulus predicting competing outcomes. The current finding that conflicting outcomes elicited different strategies tied to individual avoidance propensity might provide important insights for clinical applications, particularly in personalized interventions. For example, approach‐avoidance trainings, which have been used in clinical research and practice, in alcohol dependence (Eberl et al. 2013; Kakoschke et al. 2017), and social anxiety disorder (Asnaani et al. 2014; Yamamori et al. 2023), could be optimized by stratifying patients based on motivational predispositions rather than assuming uniform effects.

Gaze behaviors observed during the anticipation phase of the current experiment might provide further insights into how competing outcomes shape behavioral responses. Despite participants being unaware of the subsequent availability of approach and avoidance options, distinct fixation patterns emerged. Notably, a pronounced inclination of fixations towards the avoidance option (up) for the aversive stimulus and a relative tendency of fixations towards the approach option (down) for the appetitive stimulus were evident (compared to neutral stimuli). This is consistent with previous findings that decision makers exhibit a slight tendency to fixate more frequently on the concordant option and are very likely to have their last fixation on this option before a behavioral decision is executed (Glaholt and Reingold 2011; Krajbich and Rangel 2011). Thus, the respective fixation tendencies seem to prime the subsequent concordant behaviors. Concurrently, however, they impede the execution of the discordant behaviors (i.e., when being forced to approach the threat or avoid the reward). At the group level, participants showed the same pattern of visual orienting towards conflicting and neutral stimuli, with fixations positioned at an intermediate level between aversive and appetitive stimuli. This finding aligns with the behavioral results, wherein response times demonstrated no discernible difference between the conflicting stimulus and the neutral condition, in either forced approach or avoidance behavior. However, exploratory subgroup analyses revealed this apparent “balance” masked individual differences. High avoiders displayed stronger avoidance‐oriented gaze bias towards the conflicting stimulus compared to low avoiders, with fixation patterns resembling those observed for the aversive stimulus. This heightened avoidance‐oriented gaze bias in high avoiders directly paralleled their faster avoidance responses to the conflicting stimulus, suggesting a tight coupling between threat‐focused visual exploration and avoidance behavior. These findings imply that competing outcomes did not universally promote neutral visual exploration but instead expose latent attentional biases: high avoiders' threat hypervigilance was reflected in their gaze patterns, which in turn corresponded to their behavioral strategies. While the observed gaze behavior aligns with theories linking visual exploration to action preparation (Orquin and Mueller Loose 2013), further studies are warranted to explore the causal relationship between visual exploration and behavioral selection in more detail.

Concerning autonomic responses, we observed a significant decrease in heart rate during the anticipation phase of the AAC task, which was comparable between all conditioned stimuli. While previous studies partly linked such heart rate deceleration (bradycardia) to defensive, freezing‐like states in both rodents (Swiercz et al. 2018) and humans (Garfinkel et al. 2014; Klaassen et al. 2021), the current findings are more in line with research showing that comparable responses also occur in the context of reward (Löw et al. 2008), as well as conflicting outcomes and thus probably reflect a general action‐preparatory mechanism independent of contextual valence, which may support processes of attentional orienting and motor control (Jennings et al. 2009; Merscher et al. 2022). In contrast, pupil diameter specifically increased for stimuli that predicted possible aversive outcomes. Consistently elevated arousal ratings were also observed for aversive and conflicting stimuli. These results suggest that the anticipation of a potential aversive stimulation elicited stronger arousal than the possibility of obtaining a reward (Merscher et al. 2022).

While the current study has many strengths, including a well‐controlled task and a multimodal characterization of approach and avoidance behaviors, several limitations should be considered when interpreting our findings. First, the results concerning heart rate and pupil diameter should be interpreted with caution, as the observed changes may be partly related to fluctuations in baseline values. As illustrated in previous studies (Lonsdorf et al. 2017), it takes a few seconds for heart rate and pupil diameter to return to baseline levels after the onset of an aversive US. However, in the approach‐avoidance conflict task, the short intertrial interval (2 s) and outcome duration (2 s) constrained the time available for complete recovery of heart rate and pupil diameter before the next trial started, especially in conditions where electrical stimulation was delivered. Since trial order was randomized, this would not induce a systematic bias, but it might attenuate differences between conditions. Second, although a positive relationship between the gaze distribution during the anticipation phase for the conflicting stimulus and the subsequent free avoidance choice was observed at the group level, the exact link between these two observations at the individual level remains elusive. The conflicting stimulus was associated with both the appetitive and aversive outcomes from the beginning of the experiment, which may cause an individual to have an approach tendency or an avoidance tendency before the approach‐avoidance conflict task, depending on which component is weighted more strongly. Finally, because any interpretations of differences between high avoiders and low avoiders are based on exploratory analyses, they should be viewed as hypothesis‐generating rather than hypothesis‐testing. Further research is needed to clarify the underlying mechanisms of these individual differences.

In conclusion, the present study showed that stimuli predicting a single aversive or appetitive outcome triggered rapid concordant behavioral responses, which were significantly faster than discordant responses. Stimuli associated with both reward and threat facilitated discordant responses at the group level, suggesting that competing outcomes can mitigate the challenge of overcoming automatic approach–avoidance tendencies. However, individual differences emerged in responses to conflicting stimuli, indicating that high avoiders exhibited stronger avoidance tendencies, slower approach responses, and greater threat‐focused visual attention compared to low avoiders. Thus, our results support the relevance of considering individual differences in the examination of approach and avoidance behaviors alongside the subjective value of the outcomes. These findings may provide new insights for the development of personalized clinical interventions for anxiety and addiction disorders. For instance, integrating competing outcomes into exposure therapy, such as associating a threat‐associated stimulus with a reward, may emerge as a promising strategy.

## Author Contributions

**Menghuan Chen:** conceptualization, data curation, formal analysis, investigation, methodology, project administration, software, validation, visualization, writing – original draft, writing – review and editing. **Mario Reutter:** methodology, supervision, writing – review and editing. **Paul Pauli:** conceptualization, funding acquisition, supervision, writing – review and editing. **Matthias Gamer:** conceptualization, funding acquisition, investigation, methodology, supervision, writing – review and editing. **Andre Pittig:** conceptualization, funding acquisition, investigation, methodology, supervision, writing – review and editing.

## Conflicts of Interest

The authors declare no conflicts of interest.

## Supporting information


Data S1.

## Data Availability

The experiment code and data analyzed in this study are openly available on the Open Science Framework (https://osf.io/49tdw). The code used to analyze the data is available on request from the corresponding author.
