# Supplementary material for: Overcoming Automatic Behavioral Tendencies in Approach‐Avoidance Conflict Decisions
Source: Psychophysiology. 2025 Jul 4;62(7):e70101. doi: 10.1111/psyp.70101 (PMC12232122; doi:10.1111/psyp.70101)
Supplement: Supplementary file 1 — Data S1. [file PSYP-62-e70101-s001.pdf]

Supplementary Information

Overcoming Automatic Behavioral Tendencies in Approach-Avoidance Conflict Decisions

Ratings

Results of ratings suggest that participants successfully acquired the relationships between CSs and USs and evaluated the CSs in line with the learned outcomes after the acquisition training. This learning was enhanced after the AAC task. Participants demonstrated significantly higher contingency ratings of the correct CS-US associations (i.e., contingency ratings for the correct outcome) than those of unpaired CS-US associations (i.e., contingency ratings for the incorrect outcome) after acquisition (Figure S1a) and after the AAC task (Figure S1b). Statistical details of the ANOVA analyses are provided in Supplementary Table S1, while the mean and standard deviation for each condition can be found in Supplementary Table S2. Post-hoc comparisons are reported in Supplementary Table S3.

Supplementary table S1

Repeated Measures ANOVA on subjective ratings

Items	Greenhouse-Geisser ε	df of factor	df of residuals	Mean Square	*F*	*p*	η_p^2
**Valence of CSs**							
Stage	0.843[a]	2	148	132.253	33.87	< .001	0.314
CS type	0.798[a]	3	222	808.751	134.5	< .001	0.645
Stage × CS type	0.748[a]	6	444	233.249	85.91	< .001	0.537
**Arousal of CSs**							
Stage	0.707[a]	2	148	189.938	36.87	< .001	0.333
CS type	0.721[a]	3	222	417.378	71.68	< .001	0.492
Stage × CS type	0.616[a]	6	444	105.284	38.89	< .001	0.345
**Valence of USs**							
Stage	1	1	74	5.042	2.368	0.128	0.031
US type	0.790[a]	3	222	1055.188	171.1	< .001	0.698
Stage × CS type	0.917	3	222	0.482	0.267	0.849	0.004
**Arousal of USs**							
Stage	1	1	74	3.375	2.186	0.144	0.029
CS type	0.842[a]	3	222	595.779	133.8	< .001	0.644
Stage × CS type	0.795[a]	3	222	1.144	0.595	0.581	0.008
**Contingency rating of correctly paired CS-US after acquisition training and AAC**							
Stage	1	1	74	7565.761	11.45	0.001	0.134
paired US type	0.850[a]	3	3	444.833	1.166	0.321	0.016
Stage × paired US type	0.907	3	222	51.88	0.235	0.872	0.003
**Contingency rating after acquisition training**							
appCS+: US type	1	3	222	88425.05	133.1	< .001	0.643
avCS+: US type	1	3	222	96203.61	170.2	< .001	0.697
confCS+: US type	1	3	222	97775.45	196.9	< .001	0.727
neuCS-: US type	1	3	222	106906.7	231.8	< .001	0.758
**Contingency rating after AAC task**							
appCS+: US type	1	3	222	106906.7	231.8	< .001	0.758
avCS+: US type	1	3	222	132591.7	431	< .001	0.853
confCS+: US type	1	3	222	126585.8	308.2	< .001	0.806
neuCS-: US type	1	3	222	151433.9	821.3	< .001	0.917

Note. Type III Sum of Squares. [a] Mauchly's test of sphericity indicates that the assumption of sphericity is violated (p < .05). US type included appUS, avUS, confUS, and noUS.

Supplementary table S2

Descriptive statistics of subjective ratings

Variable/stage	CS or US type			
	appCS+	avCS+	confCS+	neuCS-
	Mean (SD)	Mean (SD)	Mean (SD)	Mean (SD)
**Valence of CSs**				
Hab	6.93 (1.93)	6.93 (1.99)	7.05 (2.03)	6.65 (2.26)
Acq	8.39 (2.26)	3.11 (2.56)	4.28 (2.11)	7.21 (2.35)
AAC	9.17 (1.61)	2.05 (1.90)	3.96 (2.02)	7.77 (2.17)
**Arousal of CSs**				
Hab	3.77 (2.17)	3.69 (2.46)	4.20 (2.43)	3.96 (2.28)
Acq	4.12 (2.48)	6.75 (2.28)	6.77 (1.98)	3.21 (2.21)
AAC	4.32 (2.74)	7.19 (2.28)	7.20 (1.68)	2.68 (1.85)
**Valence of USs**				
Acq	7.95 (2.14)	2.03 (1.01)	4.03 (1.78)	6.67 (2.33)
AAC	8.24 (1.97)	2.29 (1.89)	4.13 (2.12)	6.73 (2.26)
**Arousal of USs**				
Acq	5.61 (2.36)	7.48 (1.90)	6.89 (1.54)	3.04 (2.12)
AAC	5.35 (2.26)	7.23 (1.96)	7.00 (1.57)	2.85 (1.78)
**Contingency ratings of correctly paired CS-US**				
Acq	82.77 (29.66)	84.97 (26.04)	86.54 (24.40)	86.55 (24.74)
AAC	90.60 (21.88)	91.42 (19.70)	92.34 (19.22)	94.87 (14.68)

Note. For ratings of US, US type included appUS, avUS, confUS, and noUS. Abbreviation: Hab = Habituation, Acq = Acquisition, AAC = Approach-avoidance conflict.

Supplementary table S3

Post Hoc Comparisons - CS (US) type × Stage of subjective ratings

		Mean Difference	SE	*t*	Cohen's *d*	95% CI for Cohen's *d* Lower	Upper	p_{bonf}
**Valence of CSs**								
Hab, appCS+	Acq, appCS+	-1.453	0.283	-5.128	-0.688	-1.181	-0.195	< .001
	AAC, appCS+	-2.24	0.283	-7.903	-1.061	-1.602	-0.519	< .001
	Hab, avCS+	-49.31	0.319	-24.07	-31.76	-0.511	0.511	1
	Hab, confCS+	-0.12	0.319	-0.376	-0.057	-0.568	0.454	1
	Hab, neuCS-	0.28	0.319	0.878	0.133	-0.38	0.645	1
Acq, appCS+	AAC, appCS+	-0.787	0.283	-2.776	-0.373	-0.838	0.093	0.376
	Acq, avCS+	5.28	0.319	16.556	2.501	1.638	3.363	< .001
	Acq, confCS+	4.107	0.319	12.877	1.945	1.201	2.689	< .001
	Acq, neuCS-	1.173	0.319	3.679	0.556	0.022	1.09	0.017
AAC, appCS+	AAC, avCS+	7.12	0.319	22.325	3.372	2.304	4.44	< .001
	AAC, confCS+	5.213	0.319	16.347	2.469	1.613	3.325	< .001
	AAC, neuCS-	1.4	0.319	4.39	0.663	0.12	1.206	< .001
Hab, avCS+	Acq, avCS+	3.827	0.283	13.501	1.812	1.134	2.491	< .001
	AAC, avCS+	4.88	0.283	17.218	2.311	1.524	3.098	< .001
	Hab, confCS+	-0.12	0.319	-0.376	-0.057	-0.568	0.454	1
	Hab, neuCS-	0.28	0.319	0.878	0.133	-0.38	0.645	1
Acq, avCS+	AAC, avCS+	1.053	0.283	3.716	0.499	0.024	0.974	0.015
	Acq, confCS+	-1.173	0.319	-3.679	-0.556	-1.09	-0.022	0.017
	Acq, neuCS-	-4.107	0.319	-12.88	-1.945	-2.689	-1.201	< .001
AAC, avCS+	AAC, confCS+	-1.907	0.319	-5.978	-0.903	-1.472	-0.334	< .001
	AAC, neuCS-	-5.72	0.319	-17.94	-2.709	-3.619	-1.799	< .001
Hab, confCS+	Acq, confCS+	2.773	0.283	9.785	1.313	0.731	1.896	< .001
	AAC, confCS+	3.093	0.283	10.914	1.465	0.855	2.075	< .001
	Hab, neuCS-	0.4	0.319	1.254	0.189	-0.324	0.703	1
Acq, confCS+	AAC, confCS+	0.32	0.283	1.129	0.152	-0.305	0.608	1

	Acq, neuCS-	-2.933	0.319	-9.198	-1.389	-2.03	-0.749	< .001
AAC, confCS+	AAC, neuCS-	-3.813	0.319	-11.96	-1.806	-2.522	-1.089	< .001
Hab, neuCS-	Acq, neuCS-	-0.56	0.283	-1.976	-0.265	-0.725	0.195	1
	AAC, neuCS-	-1.12	0.283	-3.952	-0.53	-1.008	-0.053	0.006
Acq, neuCS-	AAC, neuCS-	-0.56	0.283	-1.976	-0.265	-0.725	0.195	1
**Arousal of CSs**								
Hab, appCS+	Acq, appCS+	-0.347	0.297	-1.165	-0.154	-0.603	0.295	1
	AAC, appCS+	-0.547	0.297	-1.838	-0.243	-0.695	0.21	1
	Hab, avCS+	0.08	0.316	0.253	0.036	-0.44	0.511	1
	Hab, confCS+	-0.427	0.316	-1.35	-0.189	-0.667	0.289	1
	Hab, neuCS-	-0.187	0.316	-0.591	-0.083	-0.559	0.393	1
Acq, appCS+	AAC, appCS+	-0.2	0.297	-0.672	-0.089	-0.537	0.359	1
	Acq, avCS+	-2.627	0.316	-8.311	-1.166	-1.741	-0.591	< .001
	Acq, confCS+	-2.653	0.316	-8.395	-1.178	-1.755	-0.601	< .001
	Acq, neuCS-	0.907	0.316	2.869	0.403	-0.086	0.891	0.282
AAC, appCS+	AAC, avCS+	-2.867	0.316	-9.07	-1.273	-1.865	-0.68	< .001
	AAC, confCS+	-2.88	0.316	-9.113	-1.279	-1.872	-0.685	< .001
	AAC, neuCS-	1.64	0.316	5.189	0.728	0.212	1.245	< .001
Hab, avCS+	Acq, avCS+	-3.053	0.297	-10.26	-1.355	-1.941	-0.77	< .001
	AAC, avCS+	-3.493	0.297	-11.74	-1.551	-2.172	-0.929	< .001
	Hab, confCS+	-0.507	0.316	-1.603	-0.225	-0.704	0.254	1
	Hab, neuCS-	-0.267	0.316	-0.844	-0.118	-0.595	0.358	1
Acq, avCS+	AAC, avCS+	-0.44	0.297	-1.479	-0.195	-0.646	0.255	1
	Acq, confCS+	-0.027	0.316	-0.084	-0.012	-0.487	0.463	1
	Acq, neuCS-	3.533	0.316	11.18	1.569	0.923	2.214	< .001
AAC, avCS+	AAC, confCS+	-0.013	0.316	-0.042	-0.006	-0.481	0.469	1
	AAC, neuCS-	4.507	0.316	14.259	2.001	1.269	2.733	< .001
Hab, confCS+	Acq, confCS+	-2.573	0.297	-8.651	-1.142	-1.691	-0.594	< .001
	AAC, confCS+	-3	0.297	-10.09	-1.332	-1.913	-0.751	< .001
	Hab, neuCS-	0.24	0.316	0.759	0.107	-0.369	0.583	1

Acq, confCS+	AAC, confCS+	-0.427	0.297	-1.434	-0.189	-0.64	0.261	1
	Acq, neuCS-	3.56	0.316	11.264	1.58	0.933	2.228	< .001
AAC, confCS+	AAC, neuCS-	4.52	0.316	14.302	2.007	1.273	2.74	< .001
Hab, neuCS-	Acq, neuCS-	0.747	0.297	2.51	0.331	-0.125	0.788	0.816
	AAC, neuCS-	1.28	0.297	4.303	0.568	0.094	1.043	0.001
Acq, neuCS-	AAC, neuCS-	0.533	0.297	1.793	0.237	-0.215	0.689	1
**Valence of USs**								
Acq, appCS+	AAC, appCS+	-0.293	0.224	-1.308	-0.148	-0.507	0.21	1
	Acq, avCS+	5.92	0.326	18.158	2.996	2.065	3.928	< .001
	Acq, confCS+	3.92	0.326	12.024	1.984	1.255	2.713	< .001
	Acq, neuCS-	1.28	0.326	3.926	0.648	0.103	1.193	0.003
AAC, appCS+	AAC, avCS+	5.947	0.326	18.24	3.01	2.075	3.944	< .001
	AAC, confCS+	4.107	0.326	12.596	2.079	1.332	2.825	< .001
	AAC, neuCS-	1.507	0.326	4.621	0.763	0.208	1.317	< .001
Acq, avCS+	AAC, avCS+	-0.267	0.224	-1.189	-0.135	-0.493	0.223	1
	Acq, confCS+	-2	0.326	-6.135	-1.012	-1.593	-0.432	< .001
	Acq, neuCS-	-4.64	0.326	-14.23	-2.348	-3.146	-1.551	< .001
AAC, avCS+	AAC, confCS+	-1.84	0.326	-5.644	-0.931	-1.503	-0.36	< .001
	AAC, neuCS-	-4.44	0.326	-13.62	-2.247	-3.025	-1.469	< .001
Acq, confCS+	AAC, confCS+	-0.107	0.224	-0.476	-0.054	-0.411	0.303	1
	Acq, neuCS-	-2.64	0.326	-8.098	-1.336	-1.959	-0.713	< .001
AAC, confCS+	AAC, neuCS-	-2.6	0.326	-7.975	-1.316	-1.936	-0.696	< .001
Acq, neuCS-	AAC, neuCS-	-0.067	0.224	-0.297	-0.034	-0.39	0.323	1
**Arousal of USs**								
Acq, appCS+	AAC, appCS+	0.267	0.221	1.208	0.136	-0.22	0.493	1
	Acq, avCS+	-1.867	0.292	-6.403	-0.955	-1.484	-0.425	< .001
	Acq, confCS+	-1.28	0.292	-4.391	-0.655	-1.153	-0.156	< .001
	Acq, neuCS-	2.573	0.292	8.827	1.316	0.737	1.895	< .001
AAC, appCS+	AAC, avCS+	-1.88	0.292	-6.449	-0.961	-1.492	-0.431	< .001
	AAC, confCS+	-1.653	0.292	-5.671	-0.845	-1.363	-0.328	< .001

	AAC, neuCS-	2.493	0.292	8.553	1.275	0.702	1.848	< .001
Acq, avCS+	AAC, avCS+	0.253	0.221	1.148	0.13	-0.227	0.486	1
	Acq, confCS+	0.587	0.292	2.012	0.3	-0.175	0.775	1
	Acq, neuCS-	4.44	0.292	15.231	2.27	1.519	3.021	< .001
AAC, avCS+	AAC, confCS+	0.227	0.292	0.778	0.116	-0.354	0.586	1
	AAC, neuCS-	4.373	0.292	15.002	2.236	1.492	2.981	< .001
Acq, confCS+	AAC, confCS+	-0.107	0.221	-0.483	-0.055	-0.41	0.301	1
	Acq, neuCS-	3.853	0.292	13.218	1.97	1.278	2.663	< .001
AAC, confCS+	AAC, neuCS-	4.147	0.292	14.224	2.12	1.399	2.842	< .001
Acq, neuCS-	AAC, neuCS-	0.187	0.221	0.846	0.095	-0.26	0.451	1

Note. Computation of Cohen’s d based on pooled error. For valence and arousal of CSs, p-value and confidence intervals adjusted for comparing a family of 66 estimates (confidence intervals corrected using the Bonferroni method). For valence and arousal of USs, p-value and confidence intervals adjusted for comparing a family of 28 estimates (confidence intervals corrected using the Bonferroni method). Abbreviation: Hab = Habituation, Acq = Acquisition, AAC = Approach-avoidance conflict.

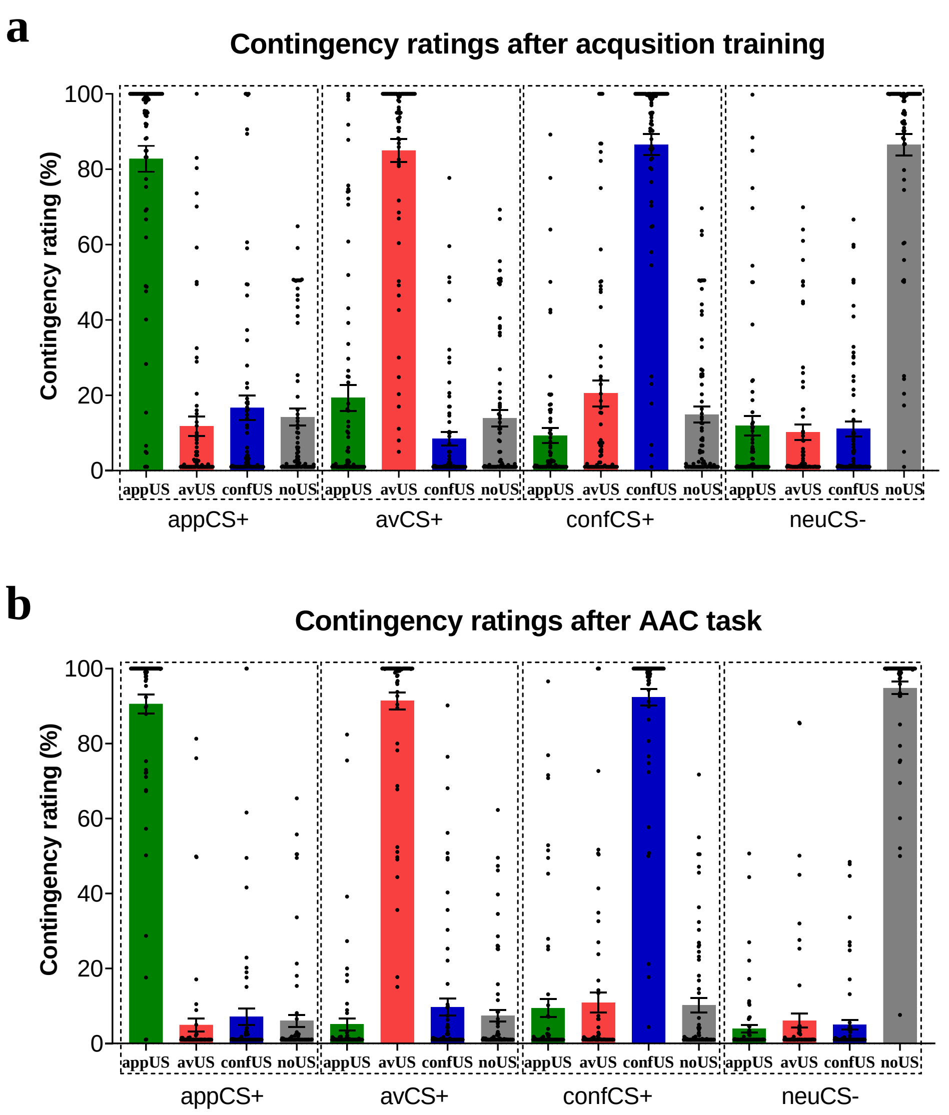

FIGURE S1. Subjective contingency ratings after acquisition training and the AAC task. Each CS was asked with the question, i.e., how likely do you think this shape is followed by the US (i.e., the money (appUS), electrical stimulation (avUS), both the electrical stimulation and the money (conflicting US, confUS), or nothing (noUS)) The four questions were presented in a pseudo-randomized order for all CSs.

Behavioral responses

Additional variables from continuous joystick movements

In addition to the response times, we also extracted additional variables based on the continuous joystick movements, including response latency, execution time, peak velocity, acceleration time, average movement velocity, and trajectory length. Definitions of these variables are summarized in Supplementary Table S4. We conducted Repeated Measures ANOVA on each of these variables to examine whether these movement measures differed as a function of the experimental manipulations and the participants' behaviors (Figure S2). Statistical details are provided in Supplementary Table S5.

The results showed that participants exhibited a significant delay in initiating joystick movement (Figure S2a) and demonstrated reduced speed throughout the execution of the joystick movement (Figure S2e) when required to overcome automatic behavioral tendencies (i.e., avoidance to the reward, approach to the threat) compared to perform concordant responses (i.e., avoidance to the

avCS+ and approach to the appCS+). These two elements collectively contributed to the differences observed in response times, as detailed in the main text (refer to Figure 4b). Moreover, participants exhibited higher peak velocity during the joystick movement for avoidance responses regardless of CS type, than approach responses ($t(73) = -2.741$, $p = 0.008$, $d = -0.276$, 95% CI [-0.479 -0.072]). There were no significant differences in either CS type or response type (forced approach vs. forced avoidance), in terms of execution time, acceleration time and trajectory length. These findings suggest that the observed behavioral modifications are specifically linked to the motivational direction of the responses rather than to the physical dynamics of the movement.

Supplementary Table S4

Definitions of the variables extracted based on the continuous joystick movements

Variable	Definition
Response latency (ms)	Time between the onset of target (i.e., manikin) and first initiation of joystick movement
Execution time (ms)	The time between the first initiation of joystick's movement and the arrival of the manikin at the target area (i.e., available open-door area)
Peak velocity (cm/ms)	The maximum action velocity, which was calculated as the rate of change of joystick position. There was a single peak of action velocity in each trial, consistent with the ballistic nature of the movement.
Acceleration time (ms)	The latency between the time of reaching peak velocity and response time.
Average movement velocity (cm/ms)	Mean of the rate of change of joystick position.
Trajectory length (cm)	The sum of the Euclidean distances between adjacent joystick positions in each trial.

Supplementary Table S5

Repeated Measures ANOVA on additional variables of joystick movements

Items	Greenhouse-Geisser ε	df of factor	df of residuals	Mean Square	*F*	*p*	η_p^2
**Response latency**							
Response type	1	1	74	689570.417	32.692	< .001	0.306
CS type	0.809[a]	2	222	39122.934	2.2	0.103	0.029
CS type × Response type	0.609[a]	3	222	1.317×10^{6}	35.005	< .001	0.321
**Execution time**							
Response type	1	1	74	710.052	0.033	0.856	4.487×10^{-4}
CS type	0.961	3	222	21297.243	1.896	0.131	0.025
CS type × Response type	0.985	3	222	51483.925	3.801	0.011	0.049
**Peak velocity**							
Response type	1	1	74	0.112	7.511	0.008	0.092
CS type	0.959	3	222	0.009	1.232	0.299	0.016
CS type × Response type	0.929	3	222	0.003	0.374	0.772	0.005
**Acceleration time**							
Response type	1	1	74	4590.688	0.638	0.427	0.009
CS type	0.986	3	222	4972.952	1.13	0.338	0.015
CS type × Response type	0.926	3	222	2473.195	0.527	0.664	0.007
**Average movement velocity**							
Response type	1	1	74	2.256×10^{-4}	2.283	0.135	0.03
CS type	0.896[a]	3	222	3.658×10^{-6}	0.055	0.976	7.492×10^{-4}
CS type × Response type	0.961	3	222	0.001	14.696	< .001	0.166
**Trajectory length**							
Response type	1	1	74	0.222	1.055	0.308	0.014
CS type	0.884[a]	3	222	0.041	0.241	0.845	0.003
CS type × Response type	0.972	3	222	0.175	0.996	0.396	0.013

Note. Type III Sum of Squares. [a] Mauchly's test of sphericity indicates that the assumption of sphericity is violated ($p < .05$).

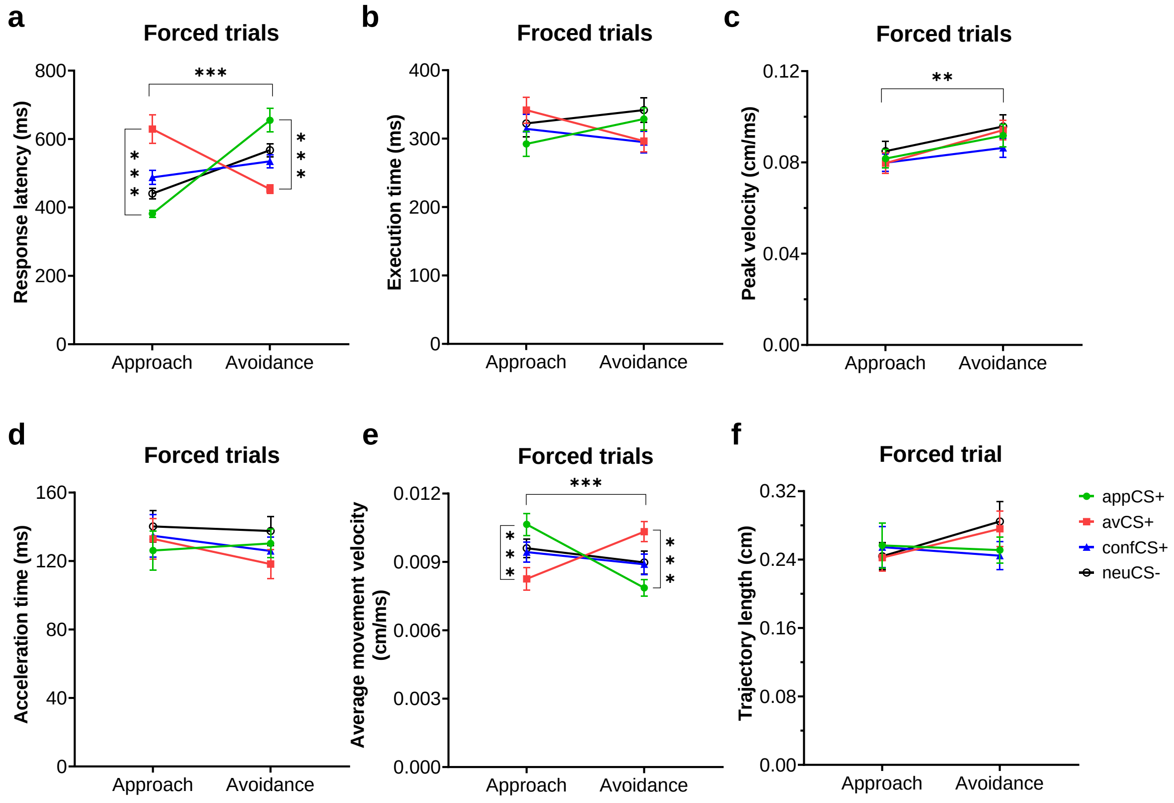

FIGURE S2. Additional variables from joystick movements in the AAC task. *(a) Response latency (b) Execution time, (c) Peak velocity during the movements, (d) Acceleration time, (e) Average movement velocity, (f) Trajectory length.*

High versus low avoiders

To investigate potential factors contributing to frequent versus infrequent avoidance to the confCS+ in free trials, we conducted additional exploratory analyses with independent Samples t-test. Results are demonstrated in Supplementary Table S6. High avoiders showed significantly faster avoidance to confCS+ in forced trials, and significantly slower approach to it in forced-approach trials compared to low avoiders (see in main text Figure 4c). Interestingly, the faster forced avoidance response observed in high avoiders was specific to confCS+ but not to the other three CSs (Supplementary Table S7 and Figure S3). Furthermore, high avoiders compared to low avoiders rated the confCS+ more negatively after acquisition training. This difference reached a significant level after the AAC task (see main text Figure 4d).

To account for multiple comparisons, we applied Bonferroni correction to all 79 comparisons in Table S6 (the application of the Bonferroni-Holm method as a less conservative approach yielded comparable results) and observed significant effects for valence ratings of confUS after the AAC task, response time of forced approach for confCS+, and average vertical gaze during anticipation for confCS+ (all corrected *ps* ≤ 0.0047). The observed large effect sizes (e.g., Cohen's |*d*| > 0.8), underscore the robustness of these effects despite the stringent corrections. For variables showing non-significance but moderate effect sizes (valence ratings of confCS+ (AAC task): Cohen's |*d*| = 0.766, response time of forced approach for confCS+: Cohen's |*d*| = 0.642, may reflect underpowered trends.

Supplementary Table S6

Independent Samples t-test of High avoiders and Low avoiders

Variables	Low avoiders (N = 43) Mean (SD)	High avoiders (N = 32) Mean (SD)	*t*	df	Cohen's *d*	95% CI for Cohen's *d*		*p* (two-tailed)	*p*bonf
						Lower	Upper		
**Sociodemographic data**									
Gender (F)	26	16							
Age (years)	24.81 (4.55)	23.25 (4.22)	1.517	73	0.354	-0.108	0.814	0.133	1.000
Intensity of electrical stimulation (mA)	0.80 (0.54)	0.83 (0.49)	-0.284	73	-0.066	-0.524	0.392	0.777	1.000
Amount of competing reward (cents)	12.60 (8.45)	16.38 (9.97)	-1.765	73	-0.412	-0.873	0.052	0.082	1.000
DASS-Depression	4.09 (4.45)	4.63 (4.41)	-0.514	73	-0.12	-0.578	0.338	0.609	1.000
DASS-Anxiety	2.74 (3.52)	3.63 (3.75)	-1.043	73	-0.243	-0.702	0.217	0.301	1.000
DASS-Stress	8.09 (5.07）	8.44 (6.24)	0.126	73	-0.062	-0.519	0.396	0.793	1.000
Intolerance of Uncertainty	37.74 (12.59)	42.09 (9.33)	-1.645	73	-0.384	-0.845	0.079	0.104	1.000
BIS	19.26 (3.36)	20.59 (3.25)	-1.729	73	-0.404	-0.865	0.06	0.088	1.000
BAS Drive	11.93 (1.76)	12.31 (2.53)	-0.77	73	-0.18	-0.638	0.279	0.443	1.000
BAS Fun Seeking	12.21 (2.09)	12.28 (2.02)	-0.15	73	-0.035	-0.492	0.423	0.881	1.000
BAS Reward Responsiveness	16.56 (2.16)	16.94 (2.18）	-0.7948	73	-0.175	-0.632	0.284	0.457	1.000
**Valence ratings of CSs**									
Valence of appCS+ (Hab)	7.00 (1.77)	6.84 (2.16)	0.344	73	0.08	-0.378	0.538	0.732	1.000
Valence of avCS+ (Hab)	6.91 (1.72)	6.97 (2.34)	-0.132	73	-0.031	-0.488	0.427	0.895	1.000
Valence of confCS+ (Hab)	6.98 (1.95)	7.16 (2.16)	-0.377	73	-0.088	-0.546	0.37	0.707	1.000
Valence of neuCS- (Hab)	6.65 (2.24)	6.66 (2.34)	-0.01	73	-0.002	-0.46	0.455	0.992	1.000
Valence of appCS+ (Acq)	8.51 (2.18)	8.22 (2.39)	0.553	73	0.129	-0.329	0.587	0.582	1.000
Valence of avCS+ (Acq)	2.81 (2.05)	3.50 (3.10)	-1.153	73	-0.269	-0.728	0.191	0.253	1.000
Valence of confCS+ (Acq)	4.63 (2.15)	3.81 (1.99)	1.676	73	0.391	-0.072	0.852	0.098	1.000
Valence of neuCS- (Acq)	7.33 (2.09)	7.06 (2.69)	0.477	73	0.111	-0.347	0.569	0.635	1.000
Valence of appCS+ (AAC)	9.35 (1.17)	8.94 (2.06)	1.093	73	0.255	-0.205	0.714	0.278	1.000
Valence of avCS+ (AAC)	2.16 (1.84)	1.90 (1.99)	0.577	73	0.135	-0.324	0.592	0.566	1.000

Valence of confCS+ (AAC)	4.58 (2.04)	3.13 (1.70)	3.281	73	0.766	0.289	1.238	0.0016	0.1264
Valence of neuCS- (AAC)	7.74 (2.01)	7.81 (2.39)	-0.134	73	-0.031	-0.489	0.426	0.894	1.000
**Arousal ratings of CSs**									
Arousal of appCS+ (Hab)	3.61 (2.22)	4.00 (2.11)	-0.78	73	-0.182	-0.64	0.277	0.438	1.000
Arousal of avCS+ (Hab)	3.63 (2.53)	3.78 (2.52)	-0.266	73	-0.062	-0.52	0.396	0.791	1.000
Arousal of confCS+ (Hab)	4.26 (2.63)	4.13 (2.17)	0.229	73	0.054	-0.404	0.511	0.819	1.000
Arousal of neuCS- (Hab)	3.93 (2.50)	4.00 (1.97)	-0.131	73	-0.03	-0.488	0.427	0.897	1.000
Arousal of appCS+ (Acq)	4.16 (2.54)	4.06 (2.54)	0.172	73	0.04	-0.418	0.498	0.864	1.000
Arousal of avCS+ (Acq)	6.58 (2.39)	6.97 (2.15)	-0.724	73	-0.169	-0.627	0.29	0.471	1.000
Arousal of confCS+ (Acq)	6.74 (2.12)	6.81 (1.80)	-0.147	73	-0.034	-0.492	0.423	0.884	1.000
Arousal of neuCS- (Acq)	3.02 (2.15)	3.47 (2.30)	-0.861	73	-0.201	-0.659	0.259	0.392	1.000
Arousal of appCS+ (AAC)	4.49 (2.86)	4.09 (2.58)	0.615	73	0.144	-0.315	0.601	0.54	1.000
Arousal of avCS+ (AAC)	6.95 (2.40)	7.50 (2.10)	-1.029	73	-0.24	-0.699	0.22	0.307	1.000
Arousal of confCS+ (AAC)	7.14 (1.78)	7.28 (1.55)	-0.36	73	-0.084	-0.542	0.374	0.72	1.000
Arousal of neuCS- (AAC)	2.65(2.01)	2.72 (1.65)	-0.155	73	-0.036	-0.494	0.422	0.877	1.000
**Valence ratings of USs**									
Valence of appUS (Acq)	8.33 (2.24)	7.44 (1.92)	1.802	73	0.421	-0.043	0.882	0.076	1.000
Valence of avUS (Acq)	2.09 (1.07)	1.94 (0.95)	0.655	73	0.153	-0.306	0.611	0.515	1.000
Valence of confUS (Acq)	4.30 (1.91)	3.66 (1.52)	1.579	73	0.369	-0.094	0.829	0.119	1.000
Valence of neuUS (Acq)	6.35 (2.45)	7.09 (2.12)	-1.38	73	-0.322	-0.782	0.14	0.172	1.000
Valence of appUS (AAC)	8.44 (1.93)	7.97 (2.02)	1.028	73	0.24	-0.22	0.698	0.307	1.000
Valence of avUS (AAC)	2.40 (1.93)	2.16 (1.87)	0.538	73	0.126	-0.333	0.583	0.592	1.000
**Valence of confUS (AAC)**	**4.98 (1.99)**	**3.00 (1.74)**	**4.478**	**73**	**1.045**	**0.554**	**1.53**	**2.7E-05**	**0.0022**
Valence of noUS (AAC)	6.09 (2.31)	7.60 (1.90)	-2.999	73	-0.7	-1.169	-0.227	0.004	0.316
**Arousal ratings of USs**									
Arousal of appUS (Acq)	5.88 (2.35)	5.25 (2.36)	1.153	73	0.269	-0.191	0.728	0.253	1.000
Arousal of avUS (Acq)	7.23 (2.11)	7.81 (1.53)	-1.315	73	-0.307	-0.766	0.154	0.193	1.000
Arousal of confUS (Acq)	6.93 (1.64)	6.84 (1.42)	0.239	73	0.056	-0.402	0.513	0.812	1.000
Arousal of noUS (Acq)	2.70 (2.26)	3.50 (1.85)	-1.639	73	-0.383	-0.843	0.08	0.106	1.000
Arousal of appUS (AAC)	5.84 (2.34)	4.69 (2.01)	2.234	73	0.522	0.055	0.985	0.029	1.000

Arousal of avUS (AAC)	6.98 (2.09)	7.56 (1.74)	-1.288	73	-0.301	-0.76	0.16	0.202	1.000
Arousal of confUS (AAC)	7.05 (1.45)	6.94 (1.74)	0.296	73	0.069	-0.389	0.527	0.768	1.000
Arousal of noUS (AAC)	2.44 (1.58)	3.41 (1.90)	-2.4	73	-0.56	-1.025	-0.092	0.019	1.000
**Response times in forced trials**									
Approach (appCS+)	759.36 (174.52)	844.09 (204.61)	-1.932	73	-0.451	-0.913	0.014	0.057	1.000
Approach (avCS+)	1111.33 (478.81)	1066.45 (357.47)	0.446	73	0.104	-0.354	0.562	0.657	1.000
**Approach (confCS+)**	**816.60 (213.66)**	**1049.41 (259.11)**	**-4.261**	**73**	**-0.995**	**-1.477**	**-0.507**	**6E-05**	**0.0047**
Approach (neuCS-)	852.19 (227.94)	922.58 (238.10)6	-1.298	73	-0.303	-0.762	0.158	0.198	1.000
Avoidance (appCS+)	1116.58 (365.21)	1089.81 (193.58)	0.351	73	0.082	-0.376	0.54	0.726	1.000
Avoidance (avCS+)	871.18 (193.58)	872.34 (174.18)	-0.027	73	-0.006	-0.464	0.451	0.979	1.000
Avoidance (confCS+)	1018.61 (245.16)	869.32 (213.94)	2.751	73	0.642	0.171	1.11	0.0075	0.5925
Avoidance (neuCS-)	1020.62 (233.38)	1048.48 (250.96)	-0.495	73	-0.116	-0.573	0.343	0.622	1.000
**Physiological changes during CS presentation in the AAC task**									
Heart rate changes (appCS+)	-1.79 (2.05)	-2.28 (2.45)	0.949	73	0.221	-0.238	0.68	0.346	1.000
Heart rate changes (avCS+)	-1.90 (1.99)	-1.50 (1.97)	-0.844	73	-0.197	-0.655	0.262	0.402	1.000
Heart rate changes (confCS+)	-1.42 (2.09)	-1.55 (2.09)	0.276	73	0.064	-0.393	0.522	0.783	1.000
Heart rate changes (neuCS-)	-1.64 (2.10)	-1.77 (2.10)	0.252	73	0.059	-0.399	0.516	0.802	1.000
Pupil diameter changes (appCS+)	-0.04 (0.05)	-0.06 (0.06)	1.292	73	0.302	-0.16	0.761	0.2	1.000
Pupil diameter changes (avCS+)	-0.01 (0.05)	-0.02 (0.05)	1.118	73	0.261	-0.199	0.72	0.267	1.000
Pupil diameter changes (confCS+)	-0.01 (0.07)	-0.02 (0.05)	1.023	73	0.239	-0.221	0.697	0.31	1.000
Pupil diameter changes (neuCS-)	-0.03 (0.05)	-0.05 (0.06)	1.897	73	0.443	-0.022	0.905	0.062	1.000
**Oculomotor changes during CS presentation in the AAC task**									
Horizontal gaze position (appCS+)	2.57 (27.82)	-0.38 (30.44)	0.437	73	0.102	-0.356	0.56	0.663	1.000
Horizontal gaze position (avCS+)	1.35 (24.07)	-4.70(44.19)	0.761	73	0.178	-0.281	0.635	0.449	1.000
Horizontal gaze position (confCS+)	-0.28 (25.69)	0.55 (4.71)	-0.141	73	-0.033	-0.49	0.425	0.889	1.000
Horizontal gaze position (neuCS-)	2.47 (28.42)	3.92 (27.25)	-0.222	73	-0.052	-0.509	0.406	0.825	1.000
Vertical gaze position (appCS+)	0.23 (42.58)	21.30 (40.22)	-2.169	73	-0.506	-0.97	-0.04	0.033	1.000
Vertical gaze position (avCS+)	57.08 (57.90)	70.69 (51.22)	-1.057	73	-0.247	-0.705	0.213	0.294	1.000
**Vertical gaze position (confCS+)**	**7.02 (46.68)**	**63.02 (47.71)**	**-5.073**	**73**	**-1.184**	**-1.677**	**-0.685**	**2.9E-06**	**0.0002**
Vertical gaze position (neuCS-)	20.48 (32.88)	41.47 (40.38)	-2.48	73	-0.579	-1.044	-0.11	0.015	1.000
Global center bias (appCS+)	87.58 (44.18)	100.44 (46.92)	-1.215	73	-0.284	-0.742	0.177	0.228	1.000

Global center bias (avCS+)	101.54 (59.71)	119.54 (57.45)	-1.303	73	-0.304	-0.763	0.157	0.197	1.000
Global center bias (confCS+)	86.49 (45.42)	108.13 (49.12)	-1.971	73	-0.46	-0.922	0.005	0.053	1.000
Global center bias (neuCS-)	91.75 (47.29)	97.59 (39.64)	-0.566	73	-0.132	-0.59	0.326	0.573	1.000

Supplementary Table S7

2 × 2 Mixed ANOVA on response times in forced trials

**Items**	**Mean Square**	***F*(1, 73)**	***p***	**η_p2**
**appCS+**				
Response type	$3.335\times10^{+6}$	70.935	< .001	0.493
Group	30817.633	0.325	0.57	0.004
Response type × Group	114035.451	2.426	0.124	0.032
**avCS+**				
Response type	$1.730\times10^{+6}$	21.015	< .001	0.224
Group	17530.049	0.127	0.723	0.002
Response type × Group	19446.611	0.236	0.628	0.003
**confCS+:**				
Response type	4408.815	0.15	0.699	0.002
Group	63987.794	0.805	0.372	0.011
Response type × Group	$1.339\times10^{+6}$	45.668	< .001	0.385
**neuCS-**				
Response type	794695.16	28.522	< .001	0.281
Group	88537.215	1.052	0.309	0.014
Response type × Group	16594.854	0.596	0.443	0.008

Note. Type III Sum of Squares. Within-subject factor: Response type (forced approach vs. forced avoidance). Between-subject factor: Group (high avoiders vs. low avoiders).

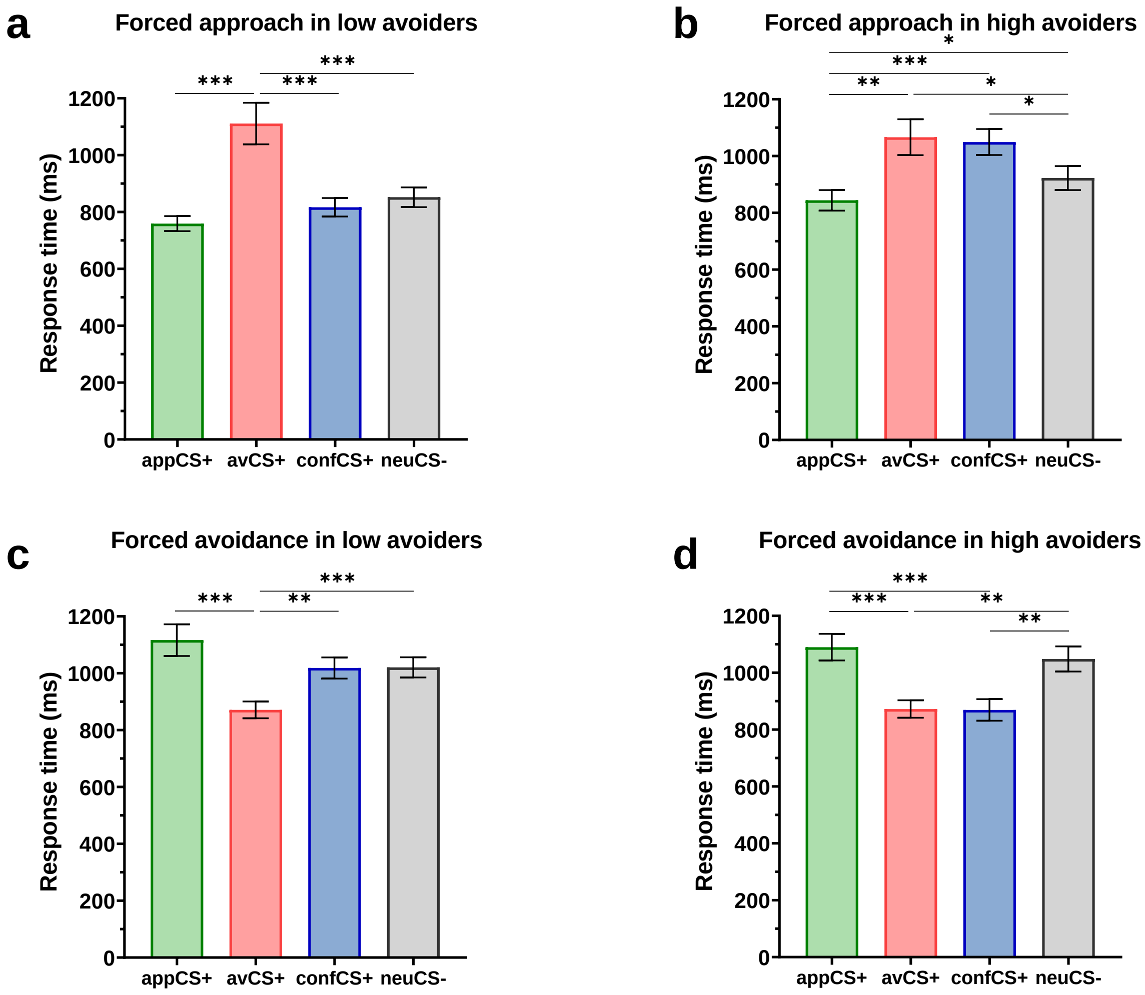

FIGURE S3. Response times of forced approach and avoidance for high versus low avoiders. *$p < 0.05$, **$p < 0.01$, ***$p < 0.001$.

Oculomotor responses

There were no significant effects of the CS type on the oculomotor responses in the habituation stage, and the acquisition training. Significant differences involving CS type only emerged in the relative vertical gaze position (Figure S4f) and global center bias (Figure S4i) during anticipation phase in the AAC task. Statistical details are provided in Supplementary Table S8.

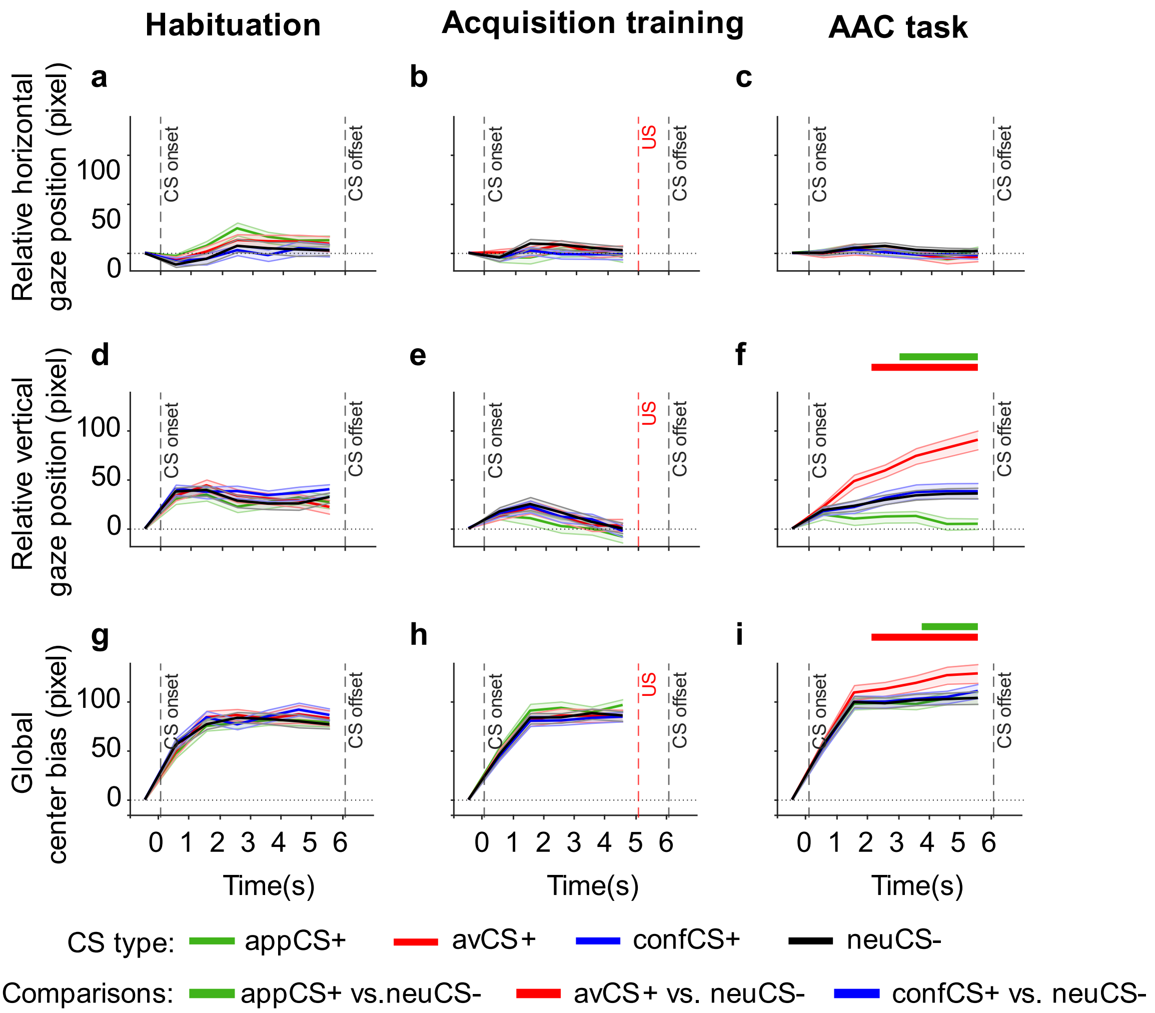

FIGURE S4. Relative horizontal, and vertical gaze position, as well as fixations' distance from center of the screen (i.e., global center bias) in the habituation stage, the acquisition training and the AAC task.

Supplementary Table S8

Repeated Measures ANOVA on oculomotor responses

Items	Greenhouse-Geisser ε	df of factor	df of residuals	Mean Square	*F*	*p*	η_p^2
**Global center bias in habituation stage**							
CS type	0.953	3	219	4592.537	0.983	0.402	0.013
Time	0.579[a]	6	438	288974	168.2	< .001	0.697
CS type × Time	0.526[a]	18	1314	1275.903	1.439	0.104	0.019
**Global center bias in acquisition training**							
CS type	0.857[a]	3	222	4750.349	1.939	0.124	0.026
Time	0.384[a]	5	370	384655.4	241.97	< .001	0.766
CS type × Time	0.629[a]	15	1110	412.355	1.245	0.231	0.017
**Global center bias in AAC task**							
CS type	0.880[a]	3	222	22022.19	1.939	0.124	0.026

Time	0.311[a]	6	444	525581	235.83	< .001	0.761
CS type × Time	0.438[a]	18	1332	1712.168	6.075	< .001	0.076

Note. Type III Sum of Squares. [a] Mauchly's test of sphericity indicates that the assumption of sphericity is violated ($p < .05$).

Post Hoc comparisons of relative vertical gaze position in the AAC task

Supplementary Table S9

Post Hoc Comparisons - CS type × Time of relative vertical gaze position in the AAC task

		Mean Difference	SE	*t*	Cohen's *d*	95% CI for Cohen's *d* Lower	Upper	p_{bonf}
appCS+, T0	avCS+, T0	3.055×10^{-13}	6.433	4.750×10^{-14}	6.136×10^{-15}	-0.498	0.498	1
	confCS+, T0	-37.03	6.433	-51.35	-63.25	-0.498	0.498	1
	neuCS-, T0	5.773×10^{-15}	6.433	8.975×10^{-16}	1.159×10^{-16}	-0.498	0.498	1
	appCS+, T1	-13.375	4.937	-2.709	-0.269	-0.66	0.123	1
	appCS+, T2	-9.492	4.937	-1.923	-0.191	-0.578	0.197	1
	appCS+, T3	-11.511	4.937	-2.332	-0.231	-0.621	0.158	1
	appCS+, T4	-12.023	4.937	-2.435	-0.241	-0.632	0.149	1
	appCS+, T5	-4.347	4.937	-0.881	-0.087	-0.471	0.296	1
	appCS+, T6	-4.563	4.937	-0.924	-0.092	-0.475	0.292	1
avCS+, T0	confCS+, T0	-67.58	6.433	-98.85	-24.96	-0.498	0.498	1
	neuCS-, T0	-42.98	6.433	-60.6	-75.2	-0.498	0.498	1
	avCS+, T1	-22.053	4.937	-4.467	-0.443	-0.85	-0.035	0.003
	avCS+, T2	-48.454	4.937	-9.815	-0.973	-1.465	-0.482	< .001
	avCS+, T3	-59.315	4.937	-12.015	-1.191	-1.729	-0.654	< .001
	avCS+, T4	-74.237	4.937	-15.037	-1.491	-2.099	-0.883	< .001
	avCS+, T5	-82.536	4.937	-16.718	-1.658	-2.308	-1.008	< .001
	avCS+, T6	-90.734	4.937	-18.379	-1.822	-2.515	-1.129	< .001
confCS+, T0	neuCS-, T0	2.460×10^{-13}	6.433	3.825×10^{-14}	4.941×10^{-15}	-0.498	0.498	1
	confCS+, T1	-17.884	4.937	-3.622	-0.359	-0.758	0.04	0.115
	confCS+, T2	-22.161	4.937	-4.489	-0.445	-0.853	-0.037	0.003
	confCS+, T3	-31.588	4.937	-6.398	-0.634	-1.067	-0.202	< .001
	confCS+, T4	-37.552	4.937	-7.606	-0.754	-1.205	-0.303	< .001
	confCS+, T5	-38.505	4.937	-7.8	-0.773	-1.228	-0.319	< .001
	confCS+, T6	-38.417	4.937	-7.782	-0.772	-1.226	-0.317	< .001
neuCS-, T0	neuCS-, T1	-18.917	4.937	-3.832	-0.38	-0.781	0.021	0.05
	neuCS-, T2	-23.2	4.937	-4.699	-0.466	-0.876	-0.056	0.001
	neuCS-, T3	-29.336	4.937	-5.942	-0.589	-1.015	-0.163	< .001
	neuCS-, T4	-33.892	4.937	-6.865	-0.681	-1.12	-0.241	< .001
	neuCS-, T5	-35.483	4.937	-7.187	-0.713	-1.157	-0.268	< .001
	neuCS-, T6	-35.787	4.937	-7.249	-0.719	-1.164	-0.273	< .001
appCS+, T1	avCS+, T1	-8.677	6.433	-1.349	-0.174	-0.676	0.327	1
	confCS+, T1	-4.509	6.433	-0.701	-0.091	-0.59	0.409	1
	neuCS-, T1	-5.541	6.433	-0.861	-0.111	-0.611	0.388	1
	appCS+, T2	3.883	4.937	0.787	0.078	-0.305	0.461	1
	appCS+, T3	1.864	4.937	0.377	0.037	-0.345	0.42	1
	appCS+, T4	1.352	4.937	0.274	0.027	-0.355	0.41	1
	appCS+, T5	9.028	4.937	1.829	0.181	-0.205	0.568	1
	appCS+, T6	8.812	4.937	1.785	0.177	-0.21	0.564	1
avCS+, T1	confCS+, T1	4.169	6.433	0.648	0.084	-0.415	0.583	1
	neuCS-, T1	3.136	6.433	0.488	0.063	-0.436	0.562	1

	avCS+, T2	-26.402	4.937	-5.348	-0.53	-0.948	-0.112	< .001
	avCS+, T3	-37.263	4.937	-7.548	-0.748	-1.198	-0.298	< .001
	avCS+, T4	-52.184	4.937	-10.57	-1.048	-1.555	-0.541	< .001
	avCS+, T5	-60.483	4.937	-12.251	-1.215	-1.758	-0.672	< .001
	avCS+, T6	-68.681	4.937	-13.912	-1.379	-1.96	-0.798	< .001
confCS+, T1	neuCS-, T1	-1.033	6.433	-0.161	-0.021	-0.519	0.478	1
	confCS+, T2	-4.277	4.937	-0.866	-0.086	-0.469	0.298	1
	confCS+, T3	-13.704	4.937	-2.776	-0.275	-0.668	0.117	1
	confCS+, T4	-19.669	4.937	-3.984	-0.395	-0.798	0.007	0.027
	confCS+, T5	-20.622	4.937	-4.177	-0.414	-0.819	-0.01	0.012
	confCS+, T6	-20.534	4.937	-4.159	-0.412	-0.817	-0.008	0.013
neuCS-, T1	neuCS-, T2	-4.283	4.937	-0.868	-0.086	-0.469	0.297	1
	neuCS-, T3	-10.419	4.937	-2.11	-0.209	-0.597	0.179	1
	neuCS-, T4	-14.976	4.937	-3.033	-0.301	-0.695	0.093	0.932
	neuCS-, T5	-16.566	4.937	-3.356	-0.333	-0.729	0.064	0.308
	neuCS-, T6	-16.871	4.937	-3.417	-0.339	-0.736	0.058	0.246
appCS+, T2	avCS+, T2	-38.962	6.433	-6.057	-0.782	-1.339	-0.226	< .001
	confCS+, T2	-12.669	6.433	-1.969	-0.254	-0.759	0.25	1
	neuCS-, T2	-13.708	6.433	-2.131	-0.275	-0.781	0.231	1
	appCS+, T3	-2.019	4.937	-0.409	-0.041	-0.423	0.342	1
	appCS+, T4	-2.531	4.937	-0.513	-0.051	-0.434	0.332	1
	appCS+, T5	5.145	4.937	1.042	0.103	-0.281	0.487	1
	appCS+, T6	4.929	4.937	0.998	0.099	-0.285	0.483	1
avCS+, T2	confCS+, T2	26.294	6.433	4.088	0.528	0.002	1.054	0.019
	neuCS-, T2	25.254	6.433	3.926	0.507	-0.016	1.031	0.037
	avCS+, T3	-10.861	4.937	-2.2	-0.218	-0.607	0.171	1
	avCS+, T4	-25.782	4.937	-5.222	-0.518	-0.934	-0.102	< .001
	avCS+, T5	-34.081	4.937	-6.903	-0.684	-1.124	-0.245	< .001
	avCS+, T6	-42.28	4.937	-8.564	-0.849	-1.317	-0.381	< .001
confCS+, T2	neuCS-, T2	-1.039	6.433	-0.162	-0.021	-0.519	0.478	1
	confCS+, T3	-9.427	4.937	-1.91	-0.189	-0.577	0.198	1
	confCS+, T4	-15.392	4.937	-3.118	-0.309	-0.704	0.086	0.704
	confCS+, T5	-16.345	4.937	-3.311	-0.328	-0.725	0.068	0.361
	confCS+, T6	-16.257	4.937	-3.293	-0.326	-0.723	0.07	0.385
neuCS-, T2	neuCS-, T3	-6.136	4.937	-1.243	-0.123	-0.508	0.261	1
	neuCS-, T4	-10.692	4.937	-2.166	-0.215	-0.603	0.174	1
	neuCS-, T5	-12.283	4.937	-2.488	-0.247	-0.637	0.144	1
	neuCS-, T6	-12.587	4.937	-2.55	-0.253	-0.644	0.138	1
appCS+, T3	avCS+, T3	-47.804	6.433	-7.432	-0.96	-1.544	-0.376	< .001
	confCS+, T3	-20.076	6.433	-3.121	-0.403	-0.918	0.111	0.723
	neuCS-, T3	-17.824	6.433	-2.771	-0.358	-0.869	0.153	1
	appCS+, T4	-0.511	4.937	-0.104	-0.01	-0.393	0.372	1
	appCS+, T5	7.164	4.937	1.451	0.144	-0.241	0.529	1
	appCS+, T6	6.949	4.937	1.407	0.14	-0.245	0.525	1
avCS+, T3	confCS+, T3	27.727	6.433	4.31	0.557	0.028	1.086	0.007
	neuCS-, T3	29.98	6.433	4.661	0.602	0.068	1.136	0.002
	avCS+, T4	-14.921	4.937	-3.022	-0.3	-0.694	0.094	0.966
	avCS+, T5	-23.221	4.937	-4.703	-0.466	-0.876	-0.056	0.001
	avCS+, T6	-31.419	4.937	-6.364	-0.631	-1.063	-0.199	< .001

confCS+, T3	neuCS-, T3	2.252	6.433	0.35	0.045	-0.453	0.544	1
	confCS+, T4	-5.965	4.937	-1.208	-0.12	-0.504	0.265	1
	confCS+, T5	-6.918	4.937	-1.401	-0.139	-0.524	0.246	1
	confCS+, T6	-6.83	4.937	-1.383	-0.137	-0.522	0.248	1
neuCS-, T3	neuCS-, T4	-4.557	4.937	-0.923	-0.092	-0.475	0.292	1
	neuCS-, T5	-6.147	4.937	-1.245	-0.123	-0.508	0.261	1
	neuCS-, T6	-6.451	4.937	-1.307	-0.13	-0.514	0.255	1
appCS+, T4	avCS+, T4	-62.214	6.433	-9.672	-1.249	-1.886	-0.613	< .001
	confCS+, T4	-25.53	6.433	-3.969	-0.513	-1.037	0.012	0.032
	neuCS-, T4	-21.87	6.433	-3.4	-0.439	-0.957	0.078	0.276
	appCS+, T5	7.676	4.937	1.555	0.154	-0.231	0.54	1
	appCS+, T6	7.46	4.937	1.511	0.15	-0.236	0.535	1
avCS+, T4	confCS+, T4	36.684	6.433	5.703	0.737	0.186	1.287	< .001
	neuCS-, T4	40.344	6.433	6.272	0.81	0.25	1.371	< .001
	avCS+, T5	-8.299	4.937	-1.681	-0.167	-0.553	0.219	1
	avCS+, T6	-16.497	4.937	-3.342	-0.331	-0.728	0.065	0.324
confCS+, T4	neuCS-, T4	3.66	6.433	0.569	0.074	-0.425	0.572	1
	confCS+, T5	-0.953	4.937	-0.193	-0.019	-0.402	0.363	1
	confCS+, T6	-0.865	4.937	-0.175	-0.017	-0.4	0.365	1
neuCS-, T4	neuCS-, T5	-1.591	4.937	-0.322	-0.032	-0.415	0.351	1
	neuCS-, T6	-1.895	4.937	-0.384	-0.038	-0.421	0.345	1
appCS+, T5	avCS+, T5	-78.189	6.433	-12.155	-1.57	-2.275	-0.866	< .001
	confCS+, T5	-34.158	6.433	-5.31	-0.686	-1.23	-0.142	< .001
	neuCS-, T5	-31.136	6.433	-4.84	-0.625	-1.162	-0.089	< .001
	appCS+, T6	-0.216	4.937	-0.044	-0.004	-0.387	0.378	1
avCS+, T5	confCS+, T5	44.03	6.433	6.845	0.884	0.312	1.456	< .001
	neuCS-, T5	47.053	6.433	7.315	0.945	0.363	1.527	< .001
	avCS+, T6	-8.198	4.937	-1.661	-0.165	-0.551	0.221	1
confCS+, T5	neuCS-, T5	3.023	6.433	0.47	0.061	-0.438	0.559	1
	confCS+, T6	0.088	4.937	0.018	0.002	-0.381	0.384	1
neuCS-, T5	neuCS-, T6	-0.304	4.937	-0.062	-0.006	-0.389	0.376	1
appCS+, T6	avCS+, T6	-86.171	6.433	-13.396	-1.731	-2.472	-0.989	< .001
	confCS+, T6	-33.855	6.433	-5.263	-0.68	-1.223	-0.137	< .001
	neuCS-, T6	-31.224	6.433	-4.854	-0.627	-1.164	-0.091	< .001
avCS+, T6	confCS+, T6	52.316	6.433	8.133	1.051	0.451	1.65	< .001
	neuCS-, T6	54.947	6.433	8.542	1.104	0.495	1.712	< .001
confCS+, T6	neuCS-, T6	2.63	6.433	0.409	0.053	-0.446	0.551	1

Note. *P*-value and confidence intervals adjusted for comparing a family of 378 estimates (confidence intervals corrected using the Bonferroni method).

Physiological responses in habituation stage

As expected, in the habituation stage, there was no significant main or interaction effect involving CS type regarding physiological responses (i.e., hear rate, pupil diameter). Statistical details are provided in Supplementary Table S10.

Supplementary Table S10

Repeated Measures ANOVA on physiological responses in habituation stage

Items	Greenhouse-Geisser ε	df of factor	df of residuals	Mean Square	*F*	*p*	η_p^2
**Heart rate**							
CS type	0.987	3	222	38.607	0.68	0.565	0.009
Time	0.327[a]	6	444	16.264	1.213	0.300	0.016
CS type × Time	0.521[a]	18	1332	3.267	0.462	0.906	0.006
**Pupil diameter**							
CS type	0.951	3	219	0.012	0.11	0.954	0.002
Time	0.313[a]	12	876	0.469	16.36	<0.001	0.183
CS type × Time	0.283[a]	36	2628	0.027	1.106	0.354	0.015

Note. Type III Sum of Squares. [a]Mauchly's test of sphericity indicates that the assumption of sphericity is violated ($p < .05$).

Post Hoc comparisons of heart rate changes in the AAC task

Supplementary Table S11

Post Hoc Comparisons - CS type × Time of heart rate changes in the AAC task

		Mean Difference	SE	*t*	Cohen's *d*	95% CI for Cohen's *d* Lower	Upper	p_{bonf}
appCS+, T0	avCS+, T0	5.390×10^{-15}	0.22	2.449×10^{-14}	2.398×10^{-15}	-0.379	0.379	1
	confCS+, T0	-52.19	0.22	-30.9	-31.55	-0.379	0.379	1
	neuCS-, T0	3.279×10^{-16}	0.22	1.490×10^{-15}	1.459×10^{-16}	-0.379	0.379	1
	appCS+, T1	0.944	0.239	3.959	0.42	-0.012	0.853	0.031
	appCS+, T2	1.141	0.239	4.781	0.507	0.066	0.949	< .001
	appCS+, T3	1.494	0.239	6.263	0.665	0.202	1.127	< .001
	appCS+, T4	2.184	0.239	9.154	0.972	0.457	1.486	< .001
	appCS+, T5	2.966	0.239	12.433	1.32	0.732	1.908	< .001
	appCS+, T6	3.243	0.239	13.594	1.443	0.827	2.059	< .001
avCS+, T0	confCS+, T0	-106.09	0.22	-55.39	-55.53	-0.379	0.379	1
	neuCS-, T0	-65.62	0.22	-37	-37.52	-0.379	0.379	1
	avCS+, T1	0.754	0.239	3.162	0.336	-0.089	0.76	0.613
	avCS+, T2	0.747	0.239	3.133	0.333	-0.092	0.757	0.676
	avCS+, T3	1.211	0.239	5.076	0.539	0.093	0.984	< .001
	avCS+, T4	1.735	0.239	7.274	0.772	0.293	1.251	< .001
	avCS+, T5	2.601	0.239	10.904	1.157	0.605	1.709	< .001
	avCS+, T6	3.317	0.239	13.906	1.476	0.852	2.1	< .001
confCS+, T0	neuCS-, T0	4.047×10^{-15}	0.22	1.839×10^{-14}	1.801×10^{-15}	-0.379	0.379	1
	confCS+, T1	0.915	0.239	3.836	0.407	-0.024	0.838	0.051
	confCS+, T2	0.691	0.239	2.897	0.308	-0.115	0.73	1
	confCS+, T3	0.756	0.239	3.169	0.336	-0.088	0.761	0.599
	confCS+, T4	1.24	0.239	5.198	0.552	0.105	0.999	< .001
	confCS+, T5	2.179	0.239	9.134	0.97	0.456	1.484	< .001
	confCS+, T6	3.056	0.239	12.81	1.36	0.763	1.957	< .001
neuCS-, T0	neuCS-, T1	0.872	0.239	3.655	0.388	-0.041	0.817	0.103
	neuCS-, T2	1.185	0.239	4.966	0.527	0.083	0.971	< .001
	neuCS-, T3	1.498	0.239	6.278	0.666	0.204	1.129	< .001
	neuCS-, T4	1.854	0.239	7.771	0.825	0.337	1.313	< .001
	neuCS-, T5	2.264	0.239	9.489	1.007	0.486	1.529	< .001
	neuCS-, T6	2.505	0.239	10.5	1.115	0.572	1.658	< .001
appCS+, T1	avCS+, T1	-0.19	0.22	-0.863	-0.085	-0.465	0.296	1
	confCS+, T1	-0.029	0.22	-0.133	-0.013	-0.392	0.366	1
	neuCS-, T1	-0.072	0.22	-0.329	-0.032	-0.412	0.347	1

	appCS+, T2	0.196	0.239	0.822	0.087	-0.325	0.499	1
	appCS+, T3	0.55	0.239	2.304	0.245	-0.174	0.663	1
	appCS+, T4	1.239	0.239	5.195	0.552	0.105	0.999	< .001
	appCS+, T5	2.022	0.239	8.475	0.9	0.399	1.401	< .001
	appCS+, T6	2.299	0.239	9.635	1.023	0.498	1.547	< .001
avCS+, T1	confCS+, T1	0.161	0.22	0.73	0.071	-0.308	0.451	1
	neuCS-, T1	0.118	0.22	0.535	0.052	-0.327	0.432	1
	avCS+, T2	-0.007	0.239	-0.029	-0.003	-0.414	0.408	1
	avCS+, T3	0.457	0.239	1.914	0.203	-0.213	0.619	1
	avCS+, T4	0.981	0.239	4.112	0.437	0.003	0.87	0.016
	avCS+, T5	1.847	0.239	7.742	0.822	0.335	1.309	< .001
	avCS+, T6	2.563	0.239	10.744	1.14	0.592	1.689	< .001
confCS+, T1	neuCS-, T1	-0.043	0.22	-0.196	-0.019	-0.398	0.36	1
	confCS+, T2	-0.224	0.239	-0.938	-0.1	-0.512	0.313	1
	confCS+, T3	-0.159	0.239	-0.667	-0.071	-0.482	0.341	1
	confCS+, T4	0.325	0.239	1.363	0.145	-0.269	0.558	1
	confCS+, T5	1.264	0.239	5.298	0.562	0.114	1.011	< .001
	confCS+, T6	2.141	0.239	8.974	0.953	0.442	1.463	< .001
neuCS-, T1	neuCS-, T2	0.313	0.239	1.311	0.139	-0.274	0.553	1
	neuCS-, T3	0.626	0.239	2.623	0.278	-0.142	0.699	1
	neuCS-, T4	0.982	0.239	4.116	0.437	0.003	0.871	0.016
	neuCS-, T5	1.392	0.239	5.834	0.619	0.163	1.075	< .001
	neuCS-, T6	1.633	0.239	6.845	0.727	0.255	1.198	< .001
appCS+, T2	avCS+, T2	-0.393	0.22	-1.786	-0.175	-0.558	0.208	1
	confCS+, T2	-0.449	0.22	-2.042	-0.2	-0.584	0.185	1
	neuCS-, T2	0.044	0.22	0.201	0.02	-0.36	0.399	1
	appCS+, T3	0.353	0.239	1.482	0.157	-0.257	0.571	1
	appCS+, T4	1.043	0.239	4.373	0.464	0.027	0.901	0.005
	appCS+, T5	1.826	0.239	7.652	0.812	0.327	1.298	< .001
	appCS+, T6	2.103	0.239	8.813	0.936	0.428	1.443	< .001
avCS+, T2	confCS+, T2	-0.056	0.22	-0.256	-0.025	-0.404	0.354	1
	neuCS-, T2	0.437	0.22	1.987	0.195	-0.19	0.579	1
	avCS+, T3	0.463	0.239	1.943	0.206	-0.21	0.623	1
	avCS+, T4	0.988	0.239	4.141	0.44	0.005	0.874	0.014
	avCS+, T5	1.854	0.239	7.771	0.825	0.337	1.313	< .001
	avCS+, T6	2.57	0.239	10.772	1.144	0.594	1.693	< .001
confCS+, T2	neuCS-, T2	0.494	0.22	2.243	0.22	-0.166	0.605	1
	confCS+, T3	0.065	0.239	0.271	0.029	-0.382	0.44	1
	confCS+, T4	0.549	0.239	2.301	0.244	-0.174	0.663	1
	confCS+, T5	1.488	0.239	6.236	0.662	0.2	1.124	< .001

	confCS+, T6	2.365	0.239	9.912	1.052	0.522	1.582	< .001
neuCS-, T2	neuCS-, T3	0.313	0.239	1.312	0.139	-0.274	0.553	1
	neuCS-, T4	0.669	0.239	2.805	0.298	-0.124	0.72	1
	neuCS-, T5	1.079	0.239	4.523	0.48	0.042	0.919	0.003
	neuCS-, T6	1.32	0.239	5.534	0.587	0.136	1.039	< .001
appCS+, T3	avCS+, T3	-0.283	0.22	-1.286	-0.126	-0.507	0.255	1
	confCS+, T3	-0.738	0.22	-3.354	-0.328	-0.722	0.065	0.321
	neuCS-, T3	0.004	0.22	0.017	0.002	-0.378	0.381	1
	appCS+, T4	0.69	0.239	2.892	0.307	-0.116	0.729	1
	appCS+, T5	1.472	0.239	6.171	0.655	0.194	1.116	< .001
	appCS+, T6	1.749	0.239	7.332	0.778	0.298	1.258	< .001
avCS+, T3	confCS+, T3	-0.455	0.22	-2.068	-0.202	-0.587	0.182	1
	neuCS-, T3	0.287	0.22	1.303	0.128	-0.254	0.509	1
	avCS+, T4	0.524	0.239	2.198	0.233	-0.184	0.651	1
	avCS+, T5	1.39	0.239	5.828	0.619	0.163	1.074	< .001
	avCS+, T6	2.106	0.239	8.83	0.937	0.429	1.445	< .001
confCS+, T3	neuCS-, T3	0.742	0.22	3.371	0.33	-0.063	0.724	0.302
	confCS+, T4	0.484	0.239	2.03	0.215	-0.201	0.632	1
	confCS+, T5	1.423	0.239	5.965	0.633	0.175	1.091	< .001
	confCS+, T6	2.3	0.239	9.641	1.023	0.499	1.548	< .001
neuCS-, T3	neuCS-, T4	0.356	0.239	1.493	0.159	-0.256	0.573	1
	neuCS-, T5	0.766	0.239	3.211	0.341	-0.084	0.766	0.519
	neuCS-, T6	1.007	0.239	4.222	0.448	0.013	0.883	0.01
appCS+, T4	avCS+, T4	-0.448	0.22	-2.038	-0.2	-0.584	0.185	1
	confCS+, T4	-0.944	0.22	-4.288	-0.42	-0.822	-0.018	0.008
	neuCS-, T4	-0.33	0.22	-1.499	-0.147	-0.529	0.235	1
	appCS+, T5	0.782	0.239	3.279	0.348	-0.078	0.774	0.41
	appCS+, T6	1.059	0.239	4.44	0.471	0.034	0.909	0.004
avCS+, T4	confCS+, T4	-0.495	0.22	-2.25	-0.22	-0.606	0.165	1
	neuCS-, T4	0.119	0.22	0.539	0.053	-0.327	0.432	1
	avCS+, T5	0.866	0.239	3.63	0.385	-0.044	0.814	0.114
	avCS+, T6	1.582	0.239	6.632	0.704	0.236	1.172	< .001
confCS+, T4	neuCS-, T4	0.614	0.22	2.789	0.273	-0.116	0.662	1
	confCS+, T5	0.939	0.239	3.935	0.418	-0.014	0.85	0.034
	confCS+, T6	1.816	0.239	7.611	0.808	0.323	1.293	< .001
neuCS-, T4	neuCS-, T5	0.41	0.239	1.718	0.182	-0.233	0.597	1
	neuCS-, T6	0.651	0.239	2.729	0.29	-0.132	0.711	1
appCS+, T5	avCS+, T5	-0.365	0.22	-1.658	-0.162	-0.545	0.22	1
	confCS+, T5	-0.787	0.22	-3.577	-0.35	-0.746	0.045	0.142
	neuCS-, T5	-0.702	0.22	-3.191	-0.313	-0.705	0.08	0.564

	appCS+, T6	0.277	0.239	1.161	0.123	-0.29	0.536	1
avCS+, T5	confCS+, T5	-0.422	0.22	-1.919	-0.188	-0.572	0.196	1
	neuCS-, T5	-0.337	0.22	-1.534	-0.15	-0.532	0.232	1
	avCS+, T6	0.716	0.239	3.002	0.319	-0.105	0.742	1
confCS+, T5	neuCS-, T5	0.085	0.22	0.386	0.038	-0.342	0.417	1
	confCS+, T6	0.877	0.239	3.676	0.39	-0.039	0.82	0.095
neuCS-, T5	neuCS-, T6	0.241	0.239	1.011	0.107	-0.305	0.52	1
appCS+, T6	avCS+, T6	0.074	0.22	0.338	0.033	-0.346	0.412	1
	confCS+, T6	-0.187	0.22	-0.85	-0.083	-0.463	0.297	1
	neuCS-, T6	-0.738	0.22	-3.354	-0.328	-0.722	0.065	0.321
avCS+, T6	confCS+, T6	-0.261	0.22	-1.188	-0.116	-0.497	0.265	1
	neuCS-, T6	-0.812	0.22	-3.692	-0.361	-0.758	0.035	0.092
confCS+, T6	neuCS-, T6	-0.551	0.22	-2.503	-0.245	-0.632	0.142	1

Note. *P*-value and confidence intervals adjusted for comparing a family of 378 estimates (confidence intervals corrected using the Bonferroni method).

Post Hoc comparisons of pupil diameter changes in the acquisition training

Supplementary Table S12

Post Hoc Comparisons - CS type × Time of pupil diameter changes in the acquisition training

						95% CI for Cohen's *d*		
		Mean Difference	SE	*t*	Cohen's *d*	Lower	Upper	p_{bonf}
appCS+, T0	avCS+, T0	-58.33	0.014	-43.33	0	-0.607	0.607	1
	confCS+, T0	-58.67	0.014	-43.57	0	-0.607	0.607	1
	neuCS-, T0	-100.31	0.014	-72.43	0	-0.607	0.607	1
	appCS+, T0.5	0.01	0.011	0.986	0.108	-3.343	3.56	1
	appCS+, T1	0.062	0.011	5.854	0.643	-2.632	3.917	< .001
	appCS+, T1.5	0.052	0.011	4.908	0.539	-2.77	3.848	< .001
	appCS+, T2	0.039	0.011	3.712	0.407	-2.945	3.76	0.199
	appCS+, T2.5	0.03	0.011	2.868	0.315	-3.068	3.698	1
	appCS+, T3	0.015	0.011	1.419	0.156	-3.28	3.592	1
	appCS+, T3.5	0.013	0.011	1.23	0.135	-3.308	3.578	1
	appCS+, T4	0.012	0.011	1.084	0.119	-3.329	3.567	1
	appCS+, T4.5	0.014	0.011	1.333	0.146	-3.293	3.585	1
	appCS+, T5	0.013	0.011	1.263	0.139	-3.303	3.58	1
avCS+, T0	confCS+, T0	-52.69	0.014	-40.04	0	-0.607	0.607	1
	neuCS-, T0	-57.98	0.014	-43.09	0	-0.607	0.607	1
	avCS+, T0.5	0.007	0.011	0.7	0.077	-3.385	3.539	1
	avCS+, T1	0.043	0.011	4.02	0.441	-2.9	3.783	0.057
	avCS+, T1.5	-0.001	0.011	-0.137	-0.015	-3.498	3.468	1
	avCS+, T2	-0.016	0.011	-1.499	-0.165	-3.598	3.268	1
	avCS+, T2.5	-0.026	0.011	-2.454	-0.269	-3.668	3.129	1
	avCS+, T3	-0.039	0.011	-3.673	-0.403	-3.757	2.951	0.231
	avCS+, T3.5	-0.058	0.011	-5.422	-0.595	-3.886	2.695	< .001
	avCS+, T4	-0.075	0.011	-7.054	-0.774	-4.005	2.457	< .001
	avCS+, T4.5	-0.084	0.011	-7.966	-0.874	-4.072	2.323	< .001
	avCS+, T5	-0.103	0.011	-9.727	-1.068	-4.202	2.066	< .001
confCS+, T0	neuCS-, T0	-57.63	0.014	-42.85	0	-0.607	0.607	1
	confCS+, T0.5	0.015	0.011	1.381	0.152	-3.286	3.589	1
	confCS+, T1	0.061	0.011	5.779	0.634	-2.643	3.912	< .001
	confCS+, T1.5	0.021	0.011	1.935	0.212	-3.205	3.63	1
	confCS+, T2	8.374×10^{-5}	0.011	0.008	8.668×10^{-4}	-3.486	3.488	1
	confCS+, T2.5	-0.01	0.011	-0.977	-0.107	-3.559	3.345	1
	confCS+, T3	-0.029	0.011	-2.76	-0.303	-3.69	3.084	1
	confCS+, T3.5	-0.044	0.011	-4.118	-0.452	-3.79	2.886	0.037
	confCS+, T4	-0.05	0.011	-4.703	-0.516	-3.833	2.8	0.003
	confCS+, T4.5	-0.059	0.011	-5.546	-0.609	-3.895	2.677	< .001
	confCS+, T5	-0.066	0.011	-6.181	-0.679	-3.941	2.584	< .001
neuCS-, T0	neuCS-, T0.5	0.004	0.011	0.333	0.037	-3.439	3.512	1
	neuCS-, T1	0.063	0.011	5.911	0.649	-2.624	3.921	< .001

	neuCS-, T1.5	0.062	0.011	5.827	0.64	-2.636	3.915	< .001
	neuCS-, T2	0.053	0.011	4.957	0.544	-2.763	3.851	< .001
	neuCS-, T2.5	0.04	0.011	3.756	0.412	-2.939	3.763	0.167
	neuCS-, T3	0.028	0.011	2.619	0.288	-3.105	3.68	1
	neuCS-, T3.5	0.024	0.011	2.246	0.247	-3.159	3.652	1
	neuCS-, T4	0.018	0.011	1.692	0.186	-3.24	3.612	1
	neuCS-, T4.5	0.014	0.011	1.361	0.149	-3.289	3.587	1
	neuCS-, T5	0.007	0.011	0.64	0.07	-3.394	3.534	1
appCS+, T0.5	avCS+, T0.5	-0.003	0.014	-0.21	-0.031	-0.639	0.576	1
	confCS+, T0.5	0.004	0.014	0.291	0.043	-0.564	0.651	1
	neuCS-, T0.5	-0.007	0.014	-0.48	-0.072	-0.679	0.536	1
	appCS+, T1	0.052	0.011	4.869	0.534	0.054	1.015	0.001
	appCS+, T1.5	0.042	0.011	3.922	0.431	-0.038	0.899	0.085
	appCS+, T2	0.029	0.011	2.726	0.299	-0.158	0.757	1
	appCS+, T2.5	0.02	0.011	1.882	0.207	-0.245	0.658	1
	appCS+, T3	0.005	0.011	0.433	0.048	-0.399	0.494	1
	appCS+, T3.5	0.003	0.011	0.244	0.027	-0.42	0.473	1
	appCS+, T4	0.001	0.011	0.099	0.011	-0.435	0.457	1
	appCS+, T4.5	0.004	0.011	0.347	0.038	-0.408	0.485	1
	appCS+, T5	0.003	0.011	0.277	0.03	-0.416	0.477	1
avCS+, T0.5	confCS+, T0.5	0.007	0.014	0.5	0.075	-0.533	0.682	1
	neuCS-, T0.5	-0.004	0.014	-0.27	-0.04	-0.648	0.567	1
	avCS+, T1	0.035	0.011	3.32	0.364	-0.098	0.827	0.865
	avCS+, T1.5	-0.009	0.011	-0.837	-0.092	-0.539	0.355	1
	avCS+, T2	-0.023	0.011	-2.2	-0.241	-0.689	0.206	1
	avCS+, T2.5	-0.033	0.011	-3.154	-0.346	-0.797	0.105	1
	avCS+, T3	-0.046	0.011	-4.374	-0.48	-0.94	-0.021	0.012
	avCS+, T3.5	-0.065	0.011	-6.123	-0.672	-1.151	-0.193	< .001
	avCS+, T4	-0.082	0.011	-7.754	-0.851	-1.355	-0.348	< .001
	avCS+, T4.5	-0.092	0.011	-8.666	-0.951	-1.471	-0.432	< .001
	avCS+, T5	-0.111	0.011	-10.427	-1.145	-1.7	-0.589	< .001
confCS+, T0.5	neuCS-, T0.5	-0.011	0.014	-0.771	-0.115	-0.723	0.493	1
	confCS+, T1	0.047	0.011	4.398	0.483	0.008	0.957	0.011
	confCS+, T1.5	0.006	0.011	0.554	0.061	-0.386	0.508	1
	confCS+, T2	-0.015	0.011	-1.373	-0.151	-0.6	0.298	1
	confCS+, T2.5	-0.025	0.011	-2.358	-0.259	-0.705	0.188	1
	confCS+, T3	-0.044	0.011	-4.142	-0.455	-0.904	-0.005	0.034
	confCS+, T3.5	-0.058	0.011	-5.5	-0.604	-1.061	-0.146	< .001
	confCS+, T4	-0.065	0.011	-6.084	-0.668	-1.131	-0.205	< .001
	confCS+, T4.5	-0.073	0.011	-6.927	-0.76	-1.232	-0.289	< .001
	confCS+, T5	-0.08	0.011	-7.562	-0.83	-1.31	-0.35	< .001
neuCS-, T0.5	neuCS-, T1	0.059	0.011	5.579	0.612	0.121	1.103	< .001
	neuCS-, T1.5	0.058	0.011	5.495	0.603	0.113	1.093	< .001
	neuCS-, T2	0.049	0.011	4.625	0.508	0.03	0.985	0.004
	neuCS-, T2.5	0.036	0.011	3.423	0.376	-0.088	0.839	0.595

	neuCS-, T3	0.024	0.011	2.286	0.251	-0.203	0.705	1
	neuCS-, T3.5	0.02	0.011	1.913	0.21	-0.242	0.662	1
	neuCS-, T4	0.014	0.011	1.359	0.149	-0.3	0.598	1
	neuCS-, T4.5	0.011	0.011	1.028	0.113	-0.335	0.561	1
	neuCS-, T5	0.003	0.011	0.307	0.034	-0.413	0.48	1
appCS+, T1	avCS+, T1	-0.019	0.014	-1.348	-0.201	-0.812	0.41	1
	confCS+, T1	-83.6	0.014	-0.055	-0.008	-0.615	0.599	1
	neuCS-, T1	6.043×10^{-4}	0.014	0.042	0.006	-0.601	0.613	1
	appCS+, T1.5	-0.01	0.011	-0.947	-0.104	-0.552	0.344	1
	appCS+, T2	-0.023	0.011	-2.142	-0.235	-0.688	0.218	1
	appCS+, T2.5	-0.032	0.011	-2.986	-0.328	-0.787	0.132	1
	appCS+, T3	-0.047	0.011	-4.436	-0.487	-0.962	-0.012	0.009
	appCS+, T3.5	-0.049	0.011	-4.625	-0.508	-0.985	-0.03	0.004
	appCS+, T4	-0.051	0.011	-4.77	-0.524	-1.003	-0.044	0.002
	appCS+, T4.5	-0.048	0.011	-4.521	-0.496	-0.972	-0.02	0.006
	appCS+, T5	-0.049	0.011	-4.591	-0.504	-0.981	-0.027	0.004
avCS+, T1	confCS+, T1	0.019	0.014	1.293	0.193	-0.417	0.804	1
	neuCS-, T1	0.02	0.014	1.39	0.208	-0.404	0.819	1
	avCS+, T1.5	-0.044	0.011	-4.157	-0.456	-0.925	0.012	0.032
	avCS+, T2	-0.059	0.011	-5.519	-0.606	-1.062	-0.15	< .001
	avCS+, T2.5	-0.069	0.011	-6.474	-0.711	-1.161	-0.261	< .001
	avCS+, T3	-0.082	0.011	-7.693	-0.845	-1.291	-0.398	< .001
	avCS+, T3.5	-0.1	0.011	-9.442	-1.037	-1.486	-0.587	< .001
	avCS+, T4	-0.117	0.011	-11.074	-1.216	-1.676	-0.756	< .001
	avCS+, T4.5	-0.127	0.011	-11.986	-1.316	-1.785	-0.847	< .001
	avCS+, T5	-0.146	0.011	-13.747	-1.509	-2.002	-1.016	< .001
confCS+, T1	neuCS-, T1	0.001	0.014	0.097	0.014	-0.593	0.622	1
	appCS+, T1.5	-0.009	0.015	-0.606	-0.096	-0.738	0.547	1
	confCS+, T1.5	-0.041	0.011	-3.844	-0.422	-0.89	0.046	0.117
	confCS+, T2	-0.061	0.011	-5.771	-0.634	-1.128	-0.14	< .001
	confCS+, T2.5	-0.072	0.011	-6.756	-0.742	-1.222	-0.262	< .001
	confCS+, T3	-0.091	0.011	-8.54	-0.937	-1.397	-0.478	< .001
	confCS+, T3.5	-0.105	0.011	-9.898	-1.087	-1.537	-0.636	< .001
	confCS+, T4	-0.111	0.011	-10.482	-1.151	-1.599	-0.703	< .001
	confCS+, T4.5	-0.12	0.011	-11.325	-1.243	-1.69	-0.797	< .001
	confCS+, T5	-0.127	0.011	-11.96	-1.313	-1.76	-0.866	< .001
neuCS-, T1	neuCS-, T1.5	-92.95	0.011	-0.084	-0.009	-0.456	0.437	1
	neuCS-, T2	-0.01	0.011	-0.954	-0.105	-0.552	0.343	1
	neuCS-, T2.5	-0.023	0.011	-2.156	-0.237	-0.69	0.217	1
	neuCS-, T3	-0.035	0.011	-3.292	-0.361	-0.824	0.101	0.953
	neuCS-, T3.5	-0.039	0.011	-3.666	-0.402	-0.869	0.064	0.238
	neuCS-, T4	-0.045	0.011	-4.219	-0.463	-0.936	0.009	0.024
	neuCS-, T4.5	-0.048	0.011	-4.55	-0.5	-0.976	-0.023	0.005
	neuCS-, T5	-0.056	0.011	-5.271	-0.579	-1.065	-0.092	< .001
appCS+, T1.5	avCS+, T1.5	-0.054	0.014	-3.708	-0.554	-1.186	0.078	0.217

	confCS+, T1.5	-0.032	0.014	-2.185	-0.326	-0.943	0.291	1
	neuCS-, T1.5	0.01	0.014	0.676	0.101	-0.507	0.709	1
	appCS+, T2	-0.013	0.011	-1.196	-0.131	-0.58	0.317	1
	appCS+, T2.5	-0.022	0.011	-2.04	-0.224	-0.676	0.229	1
	appCS+, T3	-0.037	0.011	-3.489	-0.383	-0.847	0.081	0.467
	appCS+, T3.5	-0.039	0.011	-3.678	-0.404	-0.87	0.063	0.227
	appCS+, T4	-0.041	0.011	-3.823	-0.42	-0.888	0.048	0.128
	appCS+, T4.5	-0.038	0.011	-3.574	-0.392	-0.858	0.073	0.338
	appCS+, T5	-0.039	0.011	-3.645	-0.4	-0.866	0.066	0.258
avCS+, T1.5	confCS+, T1.5	0.022	0.014	1.523	0.227	-0.383	0.838	1
	neuCS-, T1.5	0.063	0.014	4.384	0.655	0.013	1.297	0.013
	appCS+, T2	0.041	0.015	2.676	0.422	-0.233	1.078	1
	avCS+, T2	-0.014	0.011	-1.362	-0.15	-0.599	0.3	1
	avCS+, T2.5	-0.025	0.011	-2.317	-0.254	-0.709	0.2	1
	avCS+, T3	-0.038	0.011	-3.537	-0.388	-0.853	0.077	0.39
	avCS+, T3.5	-0.056	0.011	-5.285	-0.58	-1.067	-0.094	< .001
	avCS+, T4	-0.073	0.011	-6.917	-0.759	-1.273	-0.246	< .001
	avCS+, T4.5	-0.083	0.011	-7.829	-0.859	-1.39	-0.329	< .001
	avCS+, T5	-0.102	0.011	-9.59	-1.053	-1.621	-0.484	< .001
confCS+, T1.5	neuCS-, T1.5	0.041	0.014	2.861	0.427	-0.196	1.051	1
	appCS+, T2	0.019	0.015	1.235	0.195	-0.45	0.84	1
	confCS+, T2	-0.02	0.011	-1.928	-0.212	-0.663	0.24	1
	confCS+, T2.5	-0.031	0.011	-2.913	-0.32	-0.767	0.128	1
	confCS+, T3	-0.05	0.011	-4.696	-0.516	-0.963	-0.068	0.003
	confCS+, T3.5	-0.064	0.011	-6.054	-0.665	-1.118	-0.211	< .001
	confCS+, T4	-0.07	0.011	-6.638	-0.729	-1.186	-0.271	< .001
	confCS+, T4.5	-0.079	0.011	-7.481	-0.821	-1.287	-0.356	< .001
	confCS+, T5	-0.086	0.011	-8.117	-0.891	-1.364	-0.418	< .001
neuCS-, T1.5	neuCS-, T2	-0.009	0.011	-0.87	-0.096	-0.543	0.352	1
	neuCS-, T2.5	-0.022	0.011	-2.072	-0.227	-0.68	0.225	1
	neuCS-, T3	-0.034	0.011	-3.208	-0.352	-0.814	0.109	1
	neuCS-, T3.5	-0.038	0.011	-3.582	-0.393	-0.858	0.072	0.329
	neuCS-, T4	-0.044	0.011	-4.135	-0.454	-0.925	0.017	0.035
	neuCS-, T4.5	-0.047	0.011	-4.466	-0.49	-0.966	-0.015	0.008
	neuCS-, T5	-0.055	0.011	-5.187	-0.569	-1.055	-0.084	< .001
appCS+, T2	avCS+, T2	-0.055	0.014	-3.83	-0.572	-1.185	0.041	0.135
	confCS+, T2	-0.039	0.014	-2.723	-0.407	-1.029	0.216	1
	neuCS-, T2	0.013	0.014	0.915	0.137	-0.472	0.746	1
	appCS+, T2.5	-0.009	0.011	-0.844	-0.093	-0.54	0.355	1
	appCS+, T3	-0.024	0.011	-2.293	-0.252	-0.706	0.202	1
	appCS+, T3.5	-0.026	0.011	-2.482	-0.272	-0.728	0.183	1
	appCS+, T4	-0.028	0.011	-2.628	-0.288	-0.745	0.168	1
	appCS+, T4.5	-0.025	0.011	-2.379	-0.261	-0.716	0.194	1
	appCS+, T5	-0.026	0.011	-2.449	-0.269	-0.724	0.186	1
avCS+, T2	confCS+, T2	0.016	0.014	1.108	0.165	-0.444	0.775	1

	neuCS-, T2	0.068	0.014	4.746	0.709	0.088	1.329	0.002
	avCS+, T2.5	-0.01	0.011	-0.955	-0.105	-0.552	0.343	1
	avCS+, T3	-0.023	0.011	-2.174	-0.239	-0.692	0.215	1
	avCS+, T3.5	-0.042	0.011	-3.923	-0.431	-0.9	0.038	0.085
	avCS+, T4	-0.059	0.011	-5.554	-0.61	-1.1	-0.119	< .001
	avCS+, T4.5	-0.069	0.011	-6.466	-0.71	-1.215	-0.204	< .001
	avCS+, T5	-0.087	0.011	-8.227	-0.903	-1.442	-0.364	< .001
confCS+, T2	neuCS-, T2	0.052	0.014	3.638	0.543	-0.09	1.177	0.284
	confCS+, T2.5	-0.01	0.011	-0.985	-0.108	-0.556	0.34	1
	confCS+, T3	-0.029	0.011	-2.768	-0.304	-0.761	0.154	1
	confCS+, T3.5	-0.044	0.011	-4.126	-0.453	-0.924	0.018	0.036
	confCS+, T4	-0.05	0.011	-4.711	-0.517	-0.996	-0.039	0.002
	confCS+, T4.5	-0.059	0.011	-5.554	-0.61	-1.1	-0.119	< .001
	confCS+, T5	-0.066	0.011	-6.189	-0.679	-1.18	-0.179	< .001
neuCS-, T2	neuCS-, T2.5	-0.013	0.011	-1.202	-0.132	-0.58	0.317	1
	neuCS-, T3	-0.025	0.011	-2.338	-0.257	-0.711	0.198	1
	neuCS-, T3.5	-0.029	0.011	-2.712	-0.298	-0.755	0.16	1
	neuCS-, T4	-0.035	0.011	-3.265	-0.358	-0.821	0.104	1
	neuCS-, T4.5	-0.038	0.011	-3.596	-0.395	-0.86	0.071	0.311
	neuCS-, T5	-0.046	0.011	-4.317	-0.474	-0.947	-40.34	0.016
appCS+, T2.5	avCS+, T2.5	-0.056	0.014	-3.912	-0.584	-1.192	0.023	0.097
	confCS+, T2.5	-0.041	0.014	-2.826	-0.422	-1.033	0.189	1
	neuCS-, T2.5	0.009	0.014	0.653	0.097	-0.511	0.706	1
	appCS+, T3	-0.015	0.011	-1.449	-0.159	-0.609	0.29	1
	appCS+, T3.5	-0.017	0.011	-1.638	-0.18	-0.63	0.27	1
	appCS+, T4	-0.019	0.011	-1.784	-0.196	-0.647	0.255	1
	appCS+, T4.5	-0.016	0.011	-1.535	-0.168	-0.618	0.281	1
	appCS+, T5	-0.017	0.011	-1.605	-0.176	-0.626	0.274	1
avCS+, T2.5	confCS+, T2.5	0.016	0.014	1.086	0.162	-0.447	0.772	1
	neuCS-, T2.5	0.066	0.014	4.564	0.682	0.073	1.291	0.006
	avCS+, T3	-0.013	0.011	-1.219	-0.134	-0.582	0.315	1
	avCS+, T3.5	-0.031	0.011	-2.968	-0.326	-0.785	0.134	1
	avCS+, T4	-0.049	0.011	-4.6	-0.505	-0.982	-0.028	0.004
	avCS+, T4.5	-0.058	0.011	-5.512	-0.605	-1.095	-0.115	< .001
	avCS+, T5	-0.077	0.011	-7.273	-0.798	-1.318	-0.278	< .001
confCS+, T2.5	neuCS-, T2.5	0.05	0.014	3.479	0.52	-0.096	1.135	0.513
	confCS+, T3	-0.019	0.011	-1.783	-0.196	-0.647	0.255	1
	confCS+, T3.5	-0.033	0.011	-3.141	-0.345	-0.806	0.116	1
	confCS+, T4	-0.04	0.011	-3.726	-0.409	-0.876	0.058	0.188
	confCS+, T4.5	-0.048	0.011	-4.569	-0.502	-0.978	-0.025	0.005
	confCS+, T5	-0.055	0.011	-5.204	-0.571	-1.057	-0.086	< .001
neuCS-, T2.5	neuCS-, T3	-0.012	0.011	-1.137	-0.125	-0.573	0.323	1
	neuCS-, T3.5	-0.016	0.011	-1.51	-0.166	-0.615	0.284	1
	neuCS-, T4	-0.022	0.011	-2.064	-0.227	-0.679	0.226	1
	neuCS-, T4.5	-0.025	0.011	-2.395	-0.263	-0.718	0.192	1

	neuCS-, T5	-0.033	0.011	-3.116	-0.342	-0.803	0.119	1
appCS+, T3	avCS+, T3	-0.054	0.014	-3.743	-0.559	-1.172	0.054	0.19
	confCS+, T3	-0.044	0.014	-3.072	-0.459	-1.068	0.15	1
	neuCS-, T3	0.013	0.014	0.882	0.132	-0.477	0.741	1
	appCS+, T3.5	-0.002	0.011	-0.189	-0.021	-0.467	0.426	1
	appCS+, T4	-0.004	0.011	-0.334	-0.037	-0.483	0.41	1
	appCS+, T4.5	-94.86	0.011	-0.086	-0.009	-0.456	0.437	1
	appCS+, T5	-0.002	0.011	-0.156	-0.017	-0.463	0.429	1
avCS+, T3	confCS+, T3	0.01	0.014	0.671	0.1	-0.508	0.708	1
	neuCS-, T3	0.067	0.014	4.625	0.691	0.082	1.299	0.004
	avCS+, T3.5	-0.019	0.011	-1.749	-0.192	-0.643	0.259	1
	avCS+, T4	-0.036	0.011	-3.38	-0.371	-0.834	0.092	0.695
	avCS+, T4.5	-0.046	0.011	-4.292	-0.471	-0.944	0.002	0.017
	avCS+, T5	-0.064	0.011	-6.053	-0.665	-1.163	-0.166	< .001
confCS+, T3	neuCS-, T3	0.057	0.014	3.954	0.591	-0.017	1.198	0.082
	confCS+, T3.5	-0.014	0.011	-1.358	-0.149	-0.598	0.3	1
	confCS+, T4	-0.021	0.011	-1.943	-0.213	-0.665	0.239	1
	confCS+, T4.5	-0.03	0.011	-2.785	-0.306	-0.764	0.152	1
	confCS+, T5	-0.036	0.011	-3.421	-0.376	-0.839	0.088	0.6
neuCS-, T3	neuCS-, T3.5	-0.004	0.011	-0.373	-0.041	-0.487	0.406	1
	neuCS-, T4	-0.01	0.011	-0.927	-0.102	-0.549	0.346	1
	neuCS-, T4.5	-0.013	0.011	-1.258	-0.138	-0.587	0.311	1
	neuCS-, T5	-0.021	0.011	-1.979	-0.217	-0.669	0.235	1
appCS+, T3.5	avCS+, T3.5	-0.071	0.014	-4.889	-0.73	-1.357	-0.104	0.001
	confCS+, T3.5	-0.057	0.014	-3.931	-0.587	-1.203	0.029	0.09
	neuCS-, T3.5	0.011	0.014	0.747	0.112	-0.497	0.72	1
	appCS+, T4	-0.002	0.011	-0.145	-0.016	-0.462	0.43	1
	appCS+, T4.5	0.001	0.011	0.103	0.011	-0.435	0.458	1
	appCS+, T5	3.506×10^{-4}	0.011	0.033	0.004	-0.443	0.45	1
avCS+, T3.5	confCS+, T3.5	0.014	0.014	0.958	0.143	-0.466	0.752	1
	neuCS-, T3.5	0.081	0.014	5.636	0.842	0.224	1.46	< .001
	avCS+, T4	-0.017	0.011	-1.631	-0.179	-0.629	0.271	1
	avCS+, T4.5	-0.027	0.011	-2.543	-0.279	-0.735	0.177	1
	avCS+, T5	-0.046	0.011	-4.304	-0.473	-0.946	8.707×10^{-4}	0.016
confCS+, T3.5	neuCS-, T3.5	0.067	0.014	4.678	0.699	0.088	1.31	0.003
	confCS+, T4	-0.006	0.011	-0.585	-0.064	-0.511	0.383	1
	confCS+, T4.5	-0.015	0.011	-1.427	-0.157	-0.606	0.293	1
	confCS+, T5	-0.022	0.011	-2.063	-0.226	-0.679	0.226	1
neuCS-, T3.5	neuCS-, T4	-0.006	0.011	-0.554	-0.061	-0.508	0.386	1
	neuCS-, T4.5	-0.009	0.011	-0.885	-0.097	-0.545	0.35	1
	neuCS-, T5	-0.017	0.011	-1.606	-0.176	-0.626	0.274	1
appCS+, T4	avCS+, T4	-0.086	0.014	-5.982	-0.893	-1.539	-0.248	< .001
	confCS+, T4	-0.061	0.014	-4.254	-0.635	-1.257	-0.014	0.023
	neuCS-, T4	0.006	0.014	0.447	0.067	-0.541	0.674	1

	appCS+, T4.5	0.003	0.011	0.249	0.027	-0.419	0.474	1
	appCS+, T5	0.002	0.011	0.179	0.02	-0.427	0.466	1
avCS+, T4	confCS+, T4	0.025	0.014	1.728	0.258	-0.355	0.871	1
	neuCS-, T4	0.093	0.014	6.428	0.96	0.322	1.598	< .001
	avCS+, T4.5	-0.01	0.011	-0.912	-0.1	-0.548	0.347	1
	avCS+, T5	-0.028	0.011	-2.673	-0.293	-0.75	0.164	1
confCS+, T4	neuCS-, T4	0.068	0.014	4.701	0.702	0.085	1.319	0.003
	confCS+, T4.5	-0.009	0.011	-0.843	-0.093	-0.54	0.355	1
	confCS+, T5	-0.016	0.011	-1.478	-0.162	-0.612	0.287	1
neuCS-, T4	neuCS-, T4.5	-0.004	0.011	-0.331	-0.036	-0.483	0.41	1
	neuCS-, T5	-0.011	0.011	-1.052	-0.115	-0.563	0.333	1
appCS+, T4.5	avCS+, T4.5	-0.099	0.014	-6.835	-1.021	-1.675	-0.367	< .001
	confCS+, T4.5	-0.073	0.014	-5.056	-0.755	-1.382	-0.129	< .001
	neuCS-, T4.5	2.965×10^{-4}	0.014	0.021	0.003	-0.604	0.61	1
	appCS+, T5	-78.51	0.011	-0.07	-0.008	-0.454	0.439	1
avCS+, T4.5	confCS+, T4.5	0.026	0.014	1.779	0.266	-0.348	0.879	1
	neuCS-, T4.5	0.099	0.014	6.855	1.024	0.37	1.678	< .001
confCS+, T4.5	neuCS-, T4.5	0.073	0.014	5.077	0.758	0.132	1.384	< .001
	confCS+, T5	-0.007	0.011	-0.635	-0.07	-0.517	0.377	1
neuCS-, T4.5	neuCS-, T5	-0.008	0.011	-0.721	-0.079	-0.526	0.368	1
appCS+, T5	avCS+, T5	-0.117	0.014	-8.078	-1.206	-1.888	-0.524	< .001
	confCS+, T5	-0.079	0.014	-5.472	-0.817	-1.451	-0.184	< .001
	neuCS-, T5	-0.007	0.014	-0.458	-0.068	-0.676	0.539	1
avCS+, T5	confCS+, T5	0.038	0.014	2.606	0.389	-0.232	1.01	1
	neuCS-, T5	0.11	0.014	7.62	1.138	0.445	1.831	< .001
confCS+, T5	neuCS-, T5	0.072	0.014	5.014	0.749	0.109	1.389	< .001

Note. *P*-value and confidence intervals adjusted for comparing a family of 946 estimates (confidence intervals corrected using the Bonferroni method)

Post Hoc comparisons of pupil diameter changes in the AAC task

Supplementary Table S13

Post Hoc Comparisons - CS type × Time of pupil diameter changes in the AAC task

		Mean Difference	SE	*t*	Cohen's *d*	95% CI for Cohen's *d* Lower	Upper	p_{bonf}
appCS+, T0	avCS+, T0	-56.07	0.007	-69.54	-74.95	-0.45	0.45	1
	confCS+, T0	5.187×10^{-16}	0.007	7.189×10^{-14}	7.760×10^{-15}	-0.45	0.45	1
	neuCS-, T0	-47.23	0.007	-57.28	-61.71	-0.45	0.45	1
	appCS+, T0.5	0.009	0.007	1.314	0.14	-0.31	0.585	1
	appCS+, T1	0.051	0.007	7.211	0.766	0.251	1.28	< .001
	appCS+, T1.5	0.031	0.007	4.352	0.462	-0.01	0.932	0.019
	appCS+, T2	0.039	0.007	5.517	0.586	0.1	1.072	< .001
	appCS+, T2.5	0.048	0.007	6.784	0.72	0.213	1.227	< .001
	appCS+, T3	0.054	0.007	7.6	0.807	0.285	1.329	< .001
	appCS+, T3.5	0.054	0.007	7.564	0.803	0.282	1.325	< .001
	appCS+, T4	0.055	0.007	7.682	0.816	0.292	1.339	< .001
	appCS+, T4.5	0.053	0.007	7.441	0.79	0.271	1.309	< .001
	appCS+, T5	0.052	0.007	7.321	0.777	0.261	1.294	< .001
	appCS+, T5.5	0.05	0.007	7.069	0.751	0.238	1.263	< .001
	appCS+, T6	0.05	0.007	7.04	0.748	0.236	1.259	< .001
avCS+, T0	confCS+, T0	9.194×10^{-16}	0.007	1.274×10^{-13}	1.375×10^{-14}	-0.45	0.45	1
	neuCS-, T0	8.847×10^{-17}	0.007	1.226×10^{-14}	1.324×10^{-15}	-0.45	0.45	1
	avCS+, T0.5	0.008	0.007	1.161	0.123	-0.32	0.568	1
	avCS+, T1	0.049	0.007	6.898	0.732	0.223	1.241	< .001
	avCS+, T1.5	0.02	0.007	2.748	0.292	-0.16	0.746	1
	avCS+, T2	0.017	0.007	2.448	0.26	-0.19	0.712	1
	avCS+, T2.5	0.024	0.007	3.378	0.359	-0.1	0.818	0.989
	avCS+, T3	0.024	0.007	3.433	0.365	-0.1	0.825	0.808
	avCS+, T3.5	0.023	0.007	3.178	0.337	-0.12	0.795	1
	avCS+, T4	0.018	0.007	2.535	0.269	-0.18	0.722	1
	avCS+, T4.5	0.011	0.007	1.57	0.167	-0.28	0.613	1
	avCS+, T5	0.006	0.007	0.869	0.092	-0.35	0.536	1
	avCS+, T5.5	-0.003	0.007	-0.362	-0.038	-0.48	0.405	1
	avCS+, T6	-0.006	0.007	-0.841	-0.089	-0.53	0.355	1
confCS+, T0	neuCS-, T0	-99.09	0.007	-24.52	-26.43	-0.45	0.45	1
	confCS+, T0.5	0.006	0.007	0.851	0.09	-0.35	0.534	1
	confCS+, T1	0.043	0.007	6.005	0.638	0.144	1.131	< .001

	confCS+, T1.5	0.007	0.007	1.006	0.107	-0.34	0.551	1
	confCS+, T2	0.009	0.007	1.256	0.133	-0.31	0.579	1
	confCS+, T2.5	0.018	0.007	2.554	0.271	-0.18	0.724	1
	confCS+, T3	0.014	0.007	1.996	0.212	-0.24	0.661	1
	confCS+, T3.5	0.014	0.007	1.943	0.206	-0.24	0.655	1
	confCS+, T4	0.012	0.007	1.719	0.182	-0.27	0.63	1
	confCS+, T4.5	0.011	0.007	1.514	0.161	-0.29	0.607	1
	confCS+, T5	0.005	0.007	0.646	0.069	-0.38	0.512	1
	confCS+, T5.5	0.002	0.007	0.242	0.026	-0.42	0.469	1
	confCS+, T6	0.001	0.007	0.181	0.019	-0.42	0.462	1
neuCS-, T0	neuCS-, T0.5	0.01	0.007	1.344	0.143	-0.3	0.588	1
	neuCS-, T1	0.048	0.007	6.713	0.713	0.207	1.219	< .001
	neuCS-, T1.5	0.02	0.007	2.821	0.3	-0.16	0.754	1
	neuCS-, T2	0.025	0.007	3.473	0.369	-0.09	0.829	0.698
	neuCS-, T2.5	0.039	0.007	5.426	0.576	0.091	1.061	< .001
	neuCS-, T3	0.041	0.007	5.711	0.606	0.117	1.096	< .001
	neuCS-, T3.5	0.043	0.007	6.007	0.638	0.144	1.132	< .001
	neuCS-, T4	0.042	0.007	5.877	0.624	0.132	1.116	< .001
	neuCS-, T4.5	0.039	0.007	5.476	0.581	0.096	1.067	< .001
	neuCS-, T5	0.038	0.007	5.347	0.568	0.084	1.051	< .001
	neuCS-, T5.5	0.039	0.007	5.512	0.585	0.099	1.071	< .001
	neuCS-, T6	0.039	0.007	5.542	0.588	0.102	1.075	< .001
appCS+, T0.5	avCS+, T0.5	-0.001	0.007	-0.151	-0.016	-0.47	0.434	1
	confCS+, T0.5	-0.003	0.007	-0.455	-0.049	-0.5	0.401	1
	neuCS-, T0.5	2.130×10^{-4}	0.007	0.03	0.003	-0.45	0.453	1
	appCS+, T1	0.042	0.007	5.896	0.626	0.134	1.118	< .001
	appCS+, T1.5	0.022	0.007	3.037	0.323	-0.13	0.779	1
	appCS+, T2	0.03	0.007	4.203	0.446	-0.02	0.915	0.037
	appCS+, T2.5	0.039	0.007	5.47	0.581	0.095	1.066	< .001
	appCS+, T3	0.045	0.007	6.286	0.667	0.169	1.166	< .001
	appCS+, T3.5	0.044	0.007	6.249	0.664	0.166	1.161	< .001
	appCS+, T4	0.045	0.007	6.367	0.676	0.176	1.176	< .001
	appCS+, T4.5	0.043	0.007	6.127	0.651	0.155	1.146	< .001
	appCS+, T5	0.043	0.007	6.007	0.638	0.144	1.132	< .001
	appCS+, T5.5	0.041	0.007	5.755	0.611	0.121	1.101	< .001
	appCS+, T6	0.041	0.007	5.726	0.608	0.119	1.097	< .001
avCS+, T0.5	confCS+, T0.5	-0.002	0.007	-0.305	-0.033	-0.48	0.417	1
	neuCS-, T0.5	0.001	0.007	0.18	0.019	-0.43	0.47	1
	avCS+, T1	0.041	0.007	5.736	0.609	0.12	1.099	< .001

	avCS+, T1.5	0.011	0.007	1.587	0.168	-0.28	0.615	1
	avCS+, T2	0.009	0.007	1.286	0.137	-0.31	0.582	1
	avCS+, T2.5	0.016	0.007	2.216	0.235	-0.22	0.685	1
	avCS+, T3	0.016	0.007	2.272	0.241	-0.21	0.692	1
	avCS+, T3.5	0.014	0.007	2.017	0.214	-0.24	0.663	1
	avCS+, T4	0.01	0.007	1.374	0.146	-0.3	0.592	1
	avCS+, T4.5	0.003	0.007	0.409	0.043	-0.4	0.486	1
	avCS+, T5	-0.002	0.007	-0.292	-0.031	-0.47	0.412	1
	avCS+, T5.5	-0.011	0.007	-1.523	-0.162	-0.61	0.282	1
	avCS+, T6	-0.014	0.007	-2.002	-0.213	-0.66	0.23	1
confCS+, T0.5	neuCS-, T0.5	0.003	0.007	0.485	0.052	-0.4	0.503	1
	confCS+, T1	0.037	0.007	5.153	0.547	0.066	1.028	< .001
	confCS+, T1.5	0.001	0.007	0.154	0.016	-0.43	0.459	1
	confCS+, T2	0.003	0.007	0.404	0.043	-0.4	0.486	1
	confCS+, T2.5	0.012	0.007	1.702	0.181	-0.27	0.628	1
	confCS+, T3	0.008	0.007	1.145	0.122	-0.32	0.566	1
	confCS+, T3.5	0.008	0.007	1.091	0.116	-0.33	0.56	1
	confCS+, T4	0.006	0.007	0.867	0.092	-0.35	0.536	1
	confCS+, T4.5	0.005	0.007	0.663	0.07	-0.37	0.514	1
	confCS+, T5	-0.001	0.007	-0.205	-0.022	-0.47	0.421	1
	confCS+, T5.5	-0.004	0.007	-0.609	-0.065	-0.51	0.379	1
	confCS+, T6	-0.005	0.007	-0.671	-0.071	-0.52	0.372	1
neuCS-, T0.5	neuCS-, T1	0.038	0.007	5.369	0.57	0.086	1.054	< .001
	neuCS-, T1.5	0.01	0.007	1.477	0.157	-0.29	0.603	1
	neuCS-, T2	0.015	0.007	2.128	0.226	-0.22	0.676	1
	neuCS-, T2.5	0.029	0.007	4.082	0.433	-0.03	0.901	0.062
	neuCS-, T3	0.031	0.007	4.367	0.464	-0.01	0.934	0.018
	neuCS-, T3.5	0.033	0.007	4.663	0.495	0.021	0.969	0.004
	neuCS-, T4	0.032	0.007	4.532	0.481	0.009	0.954	0.008
	neuCS-, T4.5	0.029	0.007	4.132	0.439	-0.03	0.906	0.05
	neuCS-, T5	0.028	0.007	4.003	0.425	-0.04	0.891	0.086
	neuCS-, T5.5	0.03	0.007	4.168	0.443	-0.03	0.911	0.043
	neuCS-, T6	0.03	0.007	4.198	0.446	-0.02	0.914	0.037
appCS+, T1	avCS+, T1	-0.002	0.007	-0.308	-0.033	-0.48	0.417	1
	confCS+, T1	-0.009	0.007	-1.186	-0.128	-0.58	0.324	1
	neuCS-, T1	-0.004	0.007	-0.489	-0.053	-0.5	0.398	1
	appCS+, T1.5	-0.02	0.007	-2.859	-0.304	-0.76	0.151	1
	appCS+, T2	-0.012	0.007	-1.693	-0.18	-0.63	0.267	1
	appCS+, T2.5	-0.003	0.007	-0.426	-0.045	-0.49	0.398	1

	appCS+, T3	0.003	0.007	0.389	0.041	-0.4	0.484	1
	appCS+, T3.5	0.003	0.007	0.353	0.037	-0.41	0.48	1
	appCS+, T4	0.003	0.007	0.471	0.05	-0.39	0.493	1
	appCS+, T4.5	0.002	0.007	0.23	0.024	-0.42	0.467	1
	appCS+, T5	7.871×10^{-4}	0.007	0.111	0.012	-0.43	0.455	1
	appCS+, T5.5	-0.001	0.007	-0.141	-0.015	-0.46	0.428	1
	appCS+, T6	-0.001	0.007	-0.171	-0.018	-0.46	0.425	1
avCS+, T1	confCS+, T1	-0.006	0.007	-0.878	-0.095	-0.55	0.356	1
	neuCS-, T1	-0.001	0.007	-0.181	-0.02	-0.47	0.431	1
	avCS+, T1.5	-0.029	0.007	-4.15	-0.441	-0.91	0.027	0.046
	avCS+, T2	-0.032	0.007	-4.45	-0.473	-0.94	-0	0.012
	avCS+, T2.5	-0.025	0.007	-3.52	-0.374	-0.84	0.087	0.586
	avCS+, T3	-0.025	0.007	-3.464	-0.368	-0.83	0.093	0.72
	avCS+, T3.5	-0.026	0.007	-3.719	-0.395	-0.86	0.068	0.272
	avCS+, T4	-0.031	0.007	-4.362	-0.463	-0.93	0.007	0.018
	avCS+, T4.5	-0.038	0.007	-5.327	-0.566	-1.05	-0.08	< .001
	avCS+, T5	-0.043	0.007	-6.029	-0.64	-1.13	-0.15	< .001
	avCS+, T5.5	-0.052	0.007	-7.259	-0.771	-1.27	-0.27	< .001
	avCS+, T6	-0.055	0.007	-7.738	-0.822	-1.32	-0.33	< .001
confCS+, T1	neuCS-, T1	0.005	0.007	0.697	0.075	-0.38	0.526	1
	confCS+, T1.5	-0.035	0.007	-4.999	-0.531	-1.01	-0.05	< .001
	confCS+, T2	-0.034	0.007	-4.749	-0.504	-0.98	-0.03	0.003
	confCS+, T2.5	-0.024	0.007	-3.451	-0.366	-0.83	0.094	0.757
	confCS+, T3	-0.028	0.007	-4.008	-0.426	-0.89	0.041	0.084
	confCS+, T3.5	-0.029	0.007	-4.062	-0.431	-0.9	0.036	0.067
	confCS+, T4	-0.03	0.007	-4.286	-0.455	-0.93	0.014	0.025
	confCS+, T4.5	-0.032	0.007	-4.49	-0.477	-0.95	-0.01	0.01
	confCS+, T5	-0.038	0.007	-5.358	-0.569	-1.05	-0.09	< .001
	confCS+, T5.5	-0.041	0.007	-5.762	-0.612	-1.1	-0.12	< .001
	confCS+, T6	-0.041	0.007	-5.824	-0.618	-1.11	-0.13	< .001
neuCS-, T1	neuCS-, T1.5	-0.028	0.007	-3.892	-0.413	-0.88	0.052	0.136
	neuCS-, T2	-0.023	0.007	-3.24	-0.344	-0.8	0.114	1
	neuCS-, T2.5	-0.009	0.007	-1.287	-0.137	-0.58	0.309	1
	neuCS-, T3	-0.007	0.007	-1.002	-0.106	-0.55	0.338	1
	neuCS-, T3.5	-0.005	0.007	-0.706	-0.075	-0.52	0.369	1
	neuCS-, T4	-0.006	0.007	-0.836	-0.089	-0.53	0.355	1
	neuCS-, T4.5	-0.009	0.007	-1.237	-0.131	-0.58	0.314	1
	neuCS-, T5	-0.01	0.007	-1.366	-0.145	-0.59	0.301	1
	neuCS-, T5.5	-0.009	0.007	-1.201	-0.128	-0.57	0.317	1

	neuCS-, T6	-0.008	0.007	-1.171	-0.124	-0.57	0.321	1
appCS+, T1.5	avCS+, T1.5	-0.011	0.007	-1.578	-0.17	-0.62	0.284	1
	confCS+, T1.5	-0.024	0.007	-3.291	-0.355	-0.82	0.111	1
	neuCS-, T1.5	-0.011	0.007	-1.505	-0.162	-0.62	0.291	1
	appCS+, T2	0.008	0.007	1.166	0.124	-0.32	0.569	1
	appCS+, T2.5	0.017	0.007	2.433	0.258	-0.19	0.71	1
	appCS+, T3	0.023	0.007	3.248	0.345	-0.11	0.803	1
	appCS+, T3.5	0.023	0.007	3.212	0.341	-0.12	0.799	1
	appCS+, T4	0.024	0.007	3.33	0.354	-0.11	0.813	1
	appCS+, T4.5	0.022	0.007	3.089	0.328	-0.13	0.785	1
	appCS+, T5	0.021	0.007	2.97	0.315	-0.14	0.771	1
	appCS+, T5.5	0.019	0.007	2.718	0.289	-0.17	0.742	1
	appCS+, T6	0.019	0.007	2.688	0.285	-0.17	0.739	1
avCS+, T1.5	confCS+, T1.5	-0.012	0.007	-1.714	-0.185	-0.64	0.27	1
	neuCS-, T1.5	5.219×10^{-4}	0.007	0.072	0.008	-0.44	0.458	1
	avCS+, T2	-0.002	0.007	-0.3	-0.032	-0.48	0.411	1
	avCS+, T2.5	0.004	0.007	0.63	0.067	-0.38	0.51	1
	avCS+, T3	0.005	0.007	0.685	0.073	-0.37	0.516	1
	avCS+, T3.5	0.003	0.007	0.43	0.046	-0.4	0.489	1
	avCS+, T4	-0.002	0.007	-0.213	-0.023	-0.47	0.42	1
	avCS+, T4.5	-0.008	0.007	-1.178	-0.125	-0.57	0.32	1
	avCS+, T5	-0.013	0.007	-1.879	-0.2	-0.65	0.249	1
	avCS+, T5.5	-0.022	0.007	-3.11	-0.33	-0.78	0.121	1
	avCS+, T6	-0.025	0.007	-3.588	-0.381	-0.83	0.067	0.452
confCS+, T1.5	neuCS-, T1.5	0.013	0.007	1.786	0.193	-0.26	0.648	1
	confCS+, T2	0.002	0.007	0.25	0.027	-0.42	0.469	1
	confCS+, T2.5	0.011	0.007	1.548	0.164	-0.28	0.611	1
	confCS+, T3	0.007	0.007	0.991	0.105	-0.34	0.549	1
	confCS+, T3.5	0.007	0.007	0.937	0.099	-0.35	0.544	1
	confCS+, T4	0.005	0.007	0.713	0.076	-0.37	0.519	1
	confCS+, T4.5	0.004	0.007	0.508	0.054	-0.39	0.497	1
	confCS+, T5	-0.003	0.007	-0.359	-0.038	-0.48	0.405	1
	confCS+, T5.5	-0.005	0.007	-0.763	-0.081	-0.53	0.363	1
	confCS+, T6	-0.006	0.007	-0.825	-0.088	-0.53	0.356	1
neuCS-, T1.5	neuCS-, T2	0.005	0.007	0.651	0.069	-0.37	0.513	1
	neuCS-, T2.5	0.018	0.007	2.605	0.277	-0.18	0.729	1
	neuCS-, T3	0.021	0.007	2.89	0.307	-0.15	0.762	1
	neuCS-, T3.5	0.023	0.007	3.186	0.338	-0.12	0.796	1
	neuCS-, T4	0.022	0.007	3.055	0.324	-0.13	0.781	1

	neuCS-, T4.5	0.019	0.007	2.655	0.282	-0.17	0.735	1
	neuCS-, T5	0.018	0.007	2.526	0.268	-0.18	0.72	1
	neuCS-, T5.5	0.019	0.007	2.691	0.286	-0.17	0.739	1
	neuCS-, T6	0.019	0.007	2.721	0.289	-0.17	0.743	1
appCS+, T2	avCS+, T2	-0.022	0.007	-3.02	-0.326	-0.79	0.138	1
	confCS+, T2	-0.03	0.007	-4.192	-0.452	-0.93	0.024	0.042
	neuCS-, T2	-0.015	0.007	-2.011	-0.217	-0.67	0.239	1
	appCS+, T2.5	0.009	0.007	1.267	0.135	-0.31	0.58	1
	appCS+, T3	0.015	0.007	2.083	0.221	-0.23	0.67	1
	appCS+, T3.5	0.015	0.007	2.046	0.217	-0.23	0.666	1
	appCS+, T4	0.015	0.007	2.164	0.23	-0.22	0.68	1
	appCS+, T4.5	0.014	0.007	1.924	0.204	-0.24	0.653	1
	appCS+, T5	0.013	0.007	1.804	0.192	-0.26	0.639	1
	appCS+, T5.5	0.011	0.007	1.552	0.165	-0.28	0.611	1
	appCS+, T6	0.011	0.007	1.523	0.162	-0.29	0.608	1
avCS+, T2	confCS+, T2	-0.008	0.007	-1.172	-0.127	-0.58	0.326	1
	neuCS-, T2	0.007	0.007	1.009	0.109	-0.34	0.561	1
	avCS+, T2.5	0.007	0.007	0.93	0.099	-0.35	0.543	1
	avCS+, T3	0.007	0.007	0.986	0.105	-0.34	0.549	1
	avCS+, T3.5	0.005	0.007	0.731	0.078	-0.37	0.521	1
	avCS+, T4	6.227×10^{-4}	0.007	0.088	0.009	-0.43	0.452	1
	avCS+, T4.5	-0.006	0.007	-0.877	-0.093	-0.54	0.351	1
	avCS+, T5	-0.011	0.007	-1.579	-0.168	-0.61	0.279	1
	avCS+, T5.5	-0.02	0.007	-2.809	-0.298	-0.75	0.151	1
	avCS+, T6	-0.023	0.007	-3.288	-0.349	-0.8	0.098	1
confCS+, T2	neuCS-, T2	0.016	0.007	2.181	0.235	-0.22	0.693	1
	confCS+, T2.5	0.009	0.007	1.298	0.138	-0.31	0.583	1
	confCS+, T3	0.005	0.007	0.741	0.079	-0.37	0.522	1
	confCS+, T3.5	0.005	0.007	0.687	0.073	-0.37	0.516	1
	confCS+, T4	0.003	0.007	0.463	0.049	-0.39	0.492	1
	confCS+, T4.5	0.002	0.007	0.258	0.027	-0.42	0.47	1
	confCS+, T5	-0.004	0.007	-0.609	-0.065	-0.51	0.379	1
	confCS+, T5.5	-0.007	0.007	-1.013	-0.108	-0.55	0.337	1
	confCS+, T6	-0.008	0.007	-1.075	-0.114	-0.56	0.33	1
neuCS-, T2	neuCS-, T2.5	0.014	0.007	1.953	0.207	-0.24	0.656	1
	neuCS-, T3	0.016	0.007	2.239	0.238	-0.21	0.688	1
	neuCS-, T3.5	0.018	0.007	2.534	0.269	-0.18	0.721	1
	neuCS-, T4	0.017	0.007	2.404	0.255	-0.2	0.707	1
	neuCS-, T4.5	0.014	0.007	2.003	0.213	-0.24	0.662	1

	neuCS-, T5	0.013	0.007	1.875	0.199	-0.25	0.647	1
	neuCS-, T5.5	0.014	0.007	2.04	0.217	-0.23	0.666	1
	neuCS-, T6	0.015	0.007	2.069	0.22	-0.23	0.669	1
appCS+, T2.5	avCS+, T2.5	-0.024	0.007	-3.351	-0.362	-0.83	0.105	1
	confCS+, T2.5	-0.03	0.007	-4.161	-0.449	-0.93	0.027	0.048
	neuCS-, T2.5	-0.01	0.007	-1.336	-0.144	-0.6	0.309	1
	appCS+, T3	0.006	0.007	0.816	0.087	-0.36	0.53	1
	appCS+, T3.5	0.006	0.007	0.779	0.083	-0.36	0.526	1
	appCS+, T4	0.006	0.007	0.898	0.095	-0.35	0.539	1
	appCS+, T4.5	0.005	0.007	0.657	0.07	-0.37	0.513	1
	appCS+, T5	0.004	0.007	0.537	0.057	-0.39	0.5	1
	appCS+, T5.5	0.002	0.007	0.285	0.03	-0.41	0.473	1
	appCS+, T6	0.002	0.007	0.256	0.027	-0.42	0.47	1
avCS+, T2.5	confCS+, T2.5	-0.006	0.007	-0.81	-0.087	-0.54	0.364	1
	neuCS-, T2.5	0.015	0.007	2.015	0.218	-0.24	0.674	1
	avCS+, T3	3.946×10^{-4}	0.007	0.056	0.006	-0.44	0.449	1
	avCS+, T3.5	-0.001	0.007	-0.199	-0.021	-0.46	0.422	1
	avCS+, T4	-0.006	0.007	-0.842	-0.089	-0.53	0.354	1
	avCS+, T4.5	-0.013	0.007	-1.807	-0.192	-0.64	0.256	1
	avCS+, T5	-0.018	0.007	-2.509	-0.266	-0.72	0.186	1
	avCS+, T5.5	-0.027	0.007	-3.74	-0.397	-0.85	0.059	0.252
	avCS+, T6	-0.03	0.007	-4.218	-0.448	-0.9	0.004	0.034
confCS+, T2.5	neuCS-, T2.5	0.02	0.007	2.825	0.305	-0.16	0.767	1
	confCS+, T3	-0.004	0.007	-0.557	-0.059	-0.5	0.384	1
	confCS+, T3.5	-0.004	0.007	-0.611	-0.065	-0.51	0.378	1
	confCS+, T4	-0.006	0.007	-0.835	-0.089	-0.53	0.355	1
	confCS+, T4.5	-0.007	0.007	-1.04	-0.11	-0.56	0.334	1
	confCS+, T5	-0.014	0.007	-1.907	-0.203	-0.65	0.246	1
	confCS+, T5.5	-0.016	0.007	-2.311	-0.245	-0.7	0.205	1
	confCS+, T6	-0.017	0.007	-2.373	-0.252	-0.7	0.199	1
neuCS-, T2.5	neuCS-, T3	0.002	0.007	0.285	0.03	-0.41	0.473	1
	neuCS-, T3.5	0.004	0.007	0.581	0.062	-0.38	0.505	1
	neuCS-, T4	0.003	0.007	0.451	0.048	-0.4	0.491	1
	neuCS-, T4.5	3.555×10^{-4}	0.007	0.05	0.005	-0.44	0.448	1
	neuCS-, T5	-59.96	0.007	-0.079	-0.008	-0.45	0.434	1
	neuCS-, T5.5	6.119×10^{-4}	0.007	0.086	0.009	-0.43	0.452	1
	neuCS-, T6	8.238×10^{-4}	0.007	0.116	0.012	-0.43	0.455	1
appCS+, T3	avCS+, T3	-0.03	0.007	-4.099	-0.442	-0.92	0.033	0.062
	confCS+, T3	-0.04	0.007	-5.512	-0.595	-1.09	-0.1	< .001

	neuCS-, T3	-0.013	0.007	-1.858	-0.201	-0.66	0.255	1
	appCS+, T3.5	-29.9	0.007	-0.036	-0.004	-0.45	0.439	1
	appCS+, T4	5.795×10^{-4}	0.007	0.082	0.009	-0.43	0.451	1
	appCS+, T4.5	-0.001	0.007	-0.159	-0.017	-0.46	0.426	1
	appCS+, T5	-0.002	0.007	-0.279	-0.03	-0.47	0.413	1
	appCS+, T5.5	-0.004	0.007	-0.531	-0.056	-0.5	0.387	1
	appCS+, T6	-0.004	0.007	-0.56	-0.059	-0.5	0.384	1
avCS+, T3	confCS+, T3	-0.01	0.007	-1.413	-0.153	-0.61	0.301	1
	neuCS-, T3	0.016	0.007	2.241	0.242	-0.22	0.7	1
	avCS+, T3.5	-0.002	0.007	-0.255	-0.027	-0.47	0.416	1
	avCS+, T4	-0.006	0.007	-0.898	-0.095	-0.54	0.349	1
	avCS+, T4.5	-0.013	0.007	-1.863	-0.198	-0.65	0.25	1
	avCS+, T5	-0.018	0.007	-2.564	-0.272	-0.73	0.18	1
	avCS+, T5.5	-0.027	0.007	-3.795	-0.403	-0.86	0.054	0.202
	avCS+, T6	-0.03	0.007	-4.274	-0.454	-0.91	-0	0.027
confCS+, T3	neuCS-, T3	0.026	0.007	3.655	0.394	-0.08	0.864	0.37
	confCS+, T3.5	-42.25	0.007	-0.054	-0.006	-0.45	0.437	1
	confCS+, T4	-0.002	0.007	-0.278	-0.03	-0.47	0.413	1
	confCS+, T4.5	-0.003	0.007	-0.482	-0.051	-0.49	0.392	1
	confCS+, T5	-0.01	0.007	-1.35	-0.143	-0.59	0.302	1
	confCS+, T5.5	-0.012	0.007	-1.754	-0.186	-0.63	0.261	1
	confCS+, T6	-0.013	0.007	-1.816	-0.193	-0.64	0.255	1
neuCS-, T3	neuCS-, T3.5	0.002	0.007	0.295	0.031	-0.41	0.474	1
	neuCS-, T4	0.001	0.007	0.165	0.018	-0.43	0.46	1
	neuCS-, T4.5	-0.002	0.007	-0.235	-0.025	-0.47	0.418	1
	neuCS-, T5	-0.003	0.007	-0.364	-0.039	-0.48	0.404	1
	neuCS-, T5.5	-0.001	0.007	-0.199	-0.021	-0.46	0.422	1
	neuCS-, T6	-0.001	0.007	-0.169	-0.018	-0.46	0.425	1
appCS+, T3.5	avCS+, T3.5	-0.031	0.007	-4.314	-0.466	-0.94	0.012	0.025
	confCS+, T3.5	-0.04	0.007	-5.53	-0.597	-1.09	-0.1	< .001
	neuCS-, T3.5	-0.011	0.007	-1.531	-0.165	-0.62	0.288	1
	appCS+, T4	8.385×10^{-4}	0.007	0.118	0.013	-0.43	0.455	1
	appCS+, T4.5	-91.11	0.007	-0.123	-0.013	-0.46	0.43	1
	appCS+, T5	-0.002	0.007	-0.242	-0.026	-0.47	0.417	1
	appCS+, T5.5	-0.004	0.007	-0.494	-0.053	-0.5	0.391	1
	appCS+, T6	-0.004	0.007	-0.524	-0.056	-0.5	0.388	1
avCS+, T3.5	confCS+, T3.5	-0.009	0.007	-1.216	-0.131	-0.58	0.321	1
	neuCS-, T3.5	0.02	0.007	2.783	0.3	-0.16	0.762	1
	avCS+, T4	-0.005	0.007	-0.643	-0.068	-0.51	0.375	1

	avCS+, T4.5	-0.011	0.007	-1.608	-0.171	-0.62	0.276	1
	avCS+, T5	-0.016	0.007	-2.309	-0.245	-0.7	0.206	1
	avCS+, T5.5	-0.025	0.007	-3.54	-0.376	-0.83	0.079	0.543
	avCS+, T6	-0.029	0.007	-4.019	-0.427	-0.88	0.024	0.081
confCS+, T3.5	neuCS-, T3.5	0.029	0.007	3.998	0.432	-0.04	0.905	0.095
	confCS+, T4	-0.002	0.007	-0.224	-0.024	-0.47	0.419	1
	confCS+, T4.5	-0.003	0.007	-0.429	-0.046	-0.49	0.398	1
	confCS+, T5	-0.009	0.007	-1.296	-0.138	-0.58	0.308	1
	confCS+, T5.5	-0.012	0.007	-1.7	-0.181	-0.63	0.267	1
	confCS+, T6	-0.013	0.007	-1.762	-0.187	-0.64	0.26	1
neuCS-, T3.5	neuCS-, T4	-96.47	0.007	-0.13	-0.014	-0.46	0.429	1
	neuCS-, T4.5	-0.004	0.007	-0.531	-0.056	-0.5	0.387	1
	neuCS-, T5	-0.005	0.007	-0.66	-0.07	-0.51	0.373	1
	neuCS-, T5.5	-0.004	0.007	-0.495	-0.053	-0.5	0.391	1
	neuCS-, T6	-0.003	0.007	-0.465	-0.049	-0.49	0.394	1
appCS+, T4	avCS+, T4	-0.037	0.007	-5.063	-0.546	-1.03	-0.06	< .001
	confCS+, T4	-0.042	0.007	-5.866	-0.633	-1.13	-0.13	< .001
	neuCS-, T4	-0.013	0.007	-1.776	-0.192	-0.65	0.263	1
	appCS+, T4.5	-0.002	0.007	-0.241	-0.026	-0.47	0.417	1
	appCS+, T5	-0.003	0.007	-0.36	-0.038	-0.48	0.405	1
	appCS+, T5.5	-0.004	0.007	-0.613	-0.065	-0.51	0.378	1
	appCS+, T6	-0.005	0.007	-0.642	-0.068	-0.51	0.375	1
avCS+, T4	confCS+, T4	-0.006	0.007	-0.803	-0.087	-0.54	0.364	1
	neuCS-, T4	0.024	0.007	3.287	0.355	-0.11	0.821	1
	avCS+, T4.5	-0.007	0.007	-0.965	-0.102	-0.55	0.342	1
	avCS+, T5	-0.012	0.007	-1.666	-0.177	-0.62	0.27	1
	avCS+, T5.5	-0.021	0.007	-2.897	-0.308	-0.76	0.142	1
	avCS+, T6	-0.024	0.007	-3.376	-0.358	-0.81	0.089	0.995
confCS+, T4	neuCS-, T4	0.03	0.007	4.09	0.442	-0.03	0.916	0.064
	confCS+, T4.5	-0.001	0.007	-0.205	-0.022	-0.47	0.421	1
	confCS+, T5	-0.008	0.007	-1.072	-0.114	-0.56	0.331	1
	confCS+, T5.5	-0.01	0.007	-1.476	-0.157	-0.6	0.289	1
	confCS+, T6	-0.011	0.007	-1.538	-0.163	-0.61	0.283	1
neuCS-, T4	neuCS-, T4.5	-0.003	0.007	-0.401	-0.043	-0.49	0.401	1
	neuCS-, T5	-0.004	0.007	-0.529	-0.056	-0.5	0.387	1
	neuCS-, T5.5	-0.003	0.007	-0.364	-0.039	-0.48	0.404	1
	neuCS-, T6	-0.002	0.007	-0.335	-0.036	-0.48	0.407	1
appCS+, T4.5	avCS+, T4.5	-0.042	0.007	-5.775	-0.623	-1.12	-0.13	< .001
	confCS+, T4.5	-0.042	0.007	-5.83	-0.629	-1.13	-0.13	< .001

	neuCS-, T4.5	-0.014	0.007	-1.933	-0.209	-0.66	0.247	1
	appCS+, T5	-88.72	0.007	-0.119	-0.013	-0.46	0.43	1
	appCS+, T5.5	-0.003	0.007	-0.372	-0.039	-0.48	0.404	1
	appCS+, T6	-0.003	0.007	-0.401	-0.043	-0.49	0.4	1
avCS+, T4.5	confCS+, T4.5	-43.83	0.007	-0.055	-0.006	-0.46	0.444	1
	neuCS-, T4.5	0.028	0.007	3.842	0.415	-0.06	0.887	0.178
	avCS+, T5	-0.005	0.007	-0.701	-0.074	-0.52	0.369	1
	avCS+, T5.5	-0.014	0.007	-1.932	-0.205	-0.65	0.24	1
	avCS+, T6	-0.017	0.007	-2.411	-0.256	-0.7	0.188	1
confCS+, T4.5	neuCS-, T4.5	0.028	0.007	3.898	0.421	-0.05	0.893	0.142
	confCS+, T5	-0.006	0.007	-0.868	-0.092	-0.54	0.352	1
	confCS+, T5.5	-0.009	0.007	-1.272	-0.135	-0.58	0.31	1
	confCS+, T6	-0.009	0.007	-1.333	-0.142	-0.59	0.304	1
neuCS-, T4.5	neuCS-, T5	-95.51	0.007	-0.129	-0.014	-0.46	0.429	1
	neuCS-, T5.5	2.564×10^{-4}	0.007	0.036	0.004	-0.44	0.447	1
	neuCS-, T6	4.683×10^{-4}	0.007	0.066	0.007	-0.44	0.45	1
appCS+, T5	avCS+, T5	-0.046	0.007	-6.348	-0.685	-1.19	-0.18	< .001
	confCS+, T5	-0.047	0.007	-6.566	-0.709	-1.22	-0.2	< .001
	neuCS-, T5	-0.014	0.007	-1.942	-0.21	-0.67	0.246	1
	appCS+, T5.5	-0.002	0.007	-0.252	-0.027	-0.47	0.416	1
	appCS+, T6	-0.002	0.007	-0.282	-0.03	-0.47	0.413	1
avCS+, T5	confCS+, T5	-0.002	0.007	-0.219	-0.024	-0.47	0.427	1
	neuCS-, T5	0.032	0.007	4.405	0.476	-0	0.954	0.016
	avCS+, T5.5	-0.009	0.007	-1.231	-0.131	-0.57	0.312	1
	avCS+, T6	-0.012	0.007	-1.709	-0.182	-0.62	0.261	1
confCS+, T5	neuCS-, T5	0.033	0.007	4.624	0.499	0.018	0.981	0.006
	confCS+, T5.5	-0.003	0.007	-0.404	-0.043	-0.49	0.4	1
	confCS+, T6	-0.003	0.007	-0.466	-0.049	-0.49	0.394	1
neuCS-, T5	neuCS-, T5.5	0.001	0.007	0.165	0.018	-0.43	0.46	1
	neuCS-, T6	0.001	0.007	0.195	0.021	-0.42	0.464	1
appCS+, T5.5	avCS+, T5.5	-0.053	0.007	-7.31	-0.789	-1.3	-0.28	< .001
	confCS+, T5.5	-0.048	0.007	-6.716	-0.725	-1.24	-0.21	< .001
	neuCS-, T5.5	-0.011	0.007	-1.531	-0.165	-0.62	0.288	1
	appCS+, T6	-24.78	0.007	-0.029	-0.003	-0.45	0.44	1
avCS+, T5.5	confCS+, T5.5	0.004	0.007	0.595	0.064	-0.39	0.514	1
	neuCS-, T5.5	0.042	0.007	5.779	0.624	0.136	1.111	< .001
	avCS+, T6	-0.003	0.007	-0.479	-0.051	-0.49	0.392	1
confCS+, T5.5	neuCS-, T5.5	0.037	0.007	5.184	0.56	0.07	1.049	< .001
	confCS+, T6	-47.8	0.007	-0.062	-0.007	-0.45	0.436	1

neuCS-, T5.5	neuCS-, T6	2.119×10^{-4}	0.007	0.03	0.003	-0.44	0.446	1
appCS+, T6	avCS+, T6	-0.056	0.007	-7.752	-0.837	-1.34	-0.33	< .001
	confCS+, T6	-0.049	0.007	-6.747	-0.728	-1.24	-0.21	< .001
	neuCS-, T6	-0.011	0.007	-1.473	-0.159	-0.61	0.294	1
avCS+, T6	confCS+, T6	0.007	0.007	1.005	0.108	-0.34	0.559	1
	neuCS-, T6	0.045	0.007	6.279	0.678	0.196	1.159	< .001
confCS+, T6	neuCS-, T6	0.038	0.007	5.274	0.569	0.079	1.06	< .001

Note. *P*-value and confidence intervals adjusted for comparing a family of 1326 estimates (confidence intervals corrected using the Bonferroni method).

Repeated Measures ANOVA on response times of forced approach and avoidance for subgroup of low and high avoiders

Supplementary Table S14

Repeated Measures ANOVA on response times of forced approach and avoidance for subgroup of low and high avoiders

Items	Greenhouse-Geisser ε	df of factor	df of residuals	Mean Square	*F*	*p*	η_p^2
**Forced approach in low avoiders (*N* = 43)**							
CS type	0.602[a]	3	126	1.043×10+6	18.16	< .001	0.302
**Forced avoidance in low avoiders (*N* = 43)**							
CS type	0.783[a]	3	126	441116.82	14.01	< .001	0.250
**Forced approach in high avoiders (*N* = 32)**							
CS type	0.678[a]	3	93	359572.85	12.2	< .001	0.282
**Forced avoidance in high avoiders (N = 32)**							
CS type	0.827	3	93	428669.03	15.08	< .001	0.327

Note. Type III Sum of Squares. CS type: appCS+, avCS+, confCS+, and neuCS-. [a] Mauchly's test of sphericity indicates that the assumption of sphericity is violated ($p < .05$).

Post Hoc comparisons of response times of forced approach and avoidance for subgroup of low and high avoiders

Supplementary Table S15

Post Hoc Comparisons: CS type for low and high avoiders

		Mean Difference	SE	df	*t*	Cohen's *d*	95% CI for Cohen's *d*		p_{bonf}
							Lower	Upper	
**RTs for forced approach in low avoiders (*N* = 43)**									
appCS+	avCS+	-351.968	70.209	42	-5.013	-1.178	-1.919	-0.436	< .001
	confCS+	-57.234	32.253	42	-1.775	-0.191	-0.496	0.113	0.499
	neuCS-	-92.827	37.366	42	-2.484	-0.311	-0.669	0.048	0.102
avCS+	confCS+	294.734	62.978	42	4.68	0.986	0.331	1.641	< .001
	neuCS-	259.142	57.708	42	4.491	0.867	0.272	1.462	< .001
confCS+	neuCS-	-35.592	36.928	42	-0.964	-0.119	-0.463	0.225	1
**RTs for forced avoidance in low avoiders (*N* = 43)**									
appCS+	avCS+	245.402	50.125	42	4.896	0.919	0.33	1.508	< .001
	confCS+	97.966	38.88	42	2.52	0.367	-0.051	0.785	0.094
	neuCS-	95.96	35.755	42	2.684	0.359	-0.027	0.745	0.062
avCS+	confCS+	-147.436	38.517	42	-3.828	-0.552	-0.985	-0.119	0.003
	neuCS-	-149.443	32.688	42	-4.572	-0.559	-0.938	-0.181	< .001
confCS+	neuCS-	-2.006	30.522	42	-0.066	-0.008	-0.324	0.309	1
**RTs for forced approach in high avoiders (*N* = 32)**									
appCS+	avCS+	-222.362	57.672	31	-3.856	-0.821	-1.489	-0.153	0.003
	confCS+	-205.322	39.553	31	-5.191	-0.758	-1.251	-0.265	< .001
	neuCS-	-78.486	29.557	31	-2.655	-0.29	-0.614	0.035	0.074
avCS+	confCS+	17.039	40.326	31	0.423	0.063	-0.357	0.483	1
	neuCS-	143.875	45.842	31	3.138	0.531	0.018	1.045	0.022
confCS+	neuCS-	126.836	39.484	31	3.212	0.468	0.025	0.912	0.018
**RTs for forced avoidance in high avoiders (*N* = 32)**									
appCS+	avCS+	217.471	44.69	31	4.866	0.951	0.304	1.598	< .001
	confCS+	220.496	45.929	31	4.801	0.964	0.301	1.627	< .001
	neuCS-	41.338	38.226	31	1.081	0.181	-0.295	0.656	1
avCS+	confCS+	3.025	31.599	31	0.096	0.013	-0.376	0.403	1
	neuCS-	-176.134	43.33	31	-4.065	-0.77	-1.371	-0.169	0.002
confCS+	neuCS-	-179.158	47.09	31	-3.805	-0.783	-1.428	-0.139	0.004

Note. *P*-value and confidence intervals adjusted for comparing a family of 6 estimates for each ANOVA using the Bonferroni method.
